# Active protein ubiquitination regulates xylem vessel functionality

**DOI:** 10.1093/plcell/koae221

**Published:** 2024-08-02

**Authors:** Pawittra Phookaew, Ya Ma, Takaomi Suzuki, Sara Christina Stolze, Anne Harzen, Ryosuke Sano, Hirofumi Nakagami, Taku Demura, Misato Ohtani

**Affiliations:** Graduate School of Science and Technology, Division of Biological Science, Nara Institute of Science and Technology, Ikoma 630-0192, Japan; Department of Integrated Biosciences, Graduate School of Frontier Sciences, The University of Tokyo, Kashiwa 277-8562, Japan; Graduate School of Science and Technology, Division of Biological Science, Nara Institute of Science and Technology, Ikoma 630-0192, Japan; Protein Mass Spectrometry, Max Planck Institute for Plant Breeding Research, Cologne 50829, Germany; Protein Mass Spectrometry, Max Planck Institute for Plant Breeding Research, Cologne 50829, Germany; Graduate School of Science and Technology, Division of Biological Science, Nara Institute of Science and Technology, Ikoma 630-0192, Japan; Protein Mass Spectrometry, Max Planck Institute for Plant Breeding Research, Cologne 50829, Germany; Graduate School of Science and Technology, Division of Biological Science, Nara Institute of Science and Technology, Ikoma 630-0192, Japan; Center for Sustainable Resource Science, RIKEN, Yokohama 230-0045, Japan; Graduate School of Science and Technology, Division of Biological Science, Nara Institute of Science and Technology, Ikoma 630-0192, Japan; Department of Integrated Biosciences, Graduate School of Frontier Sciences, The University of Tokyo, Kashiwa 277-8562, Japan; Center for Sustainable Resource Science, RIKEN, Yokohama 230-0045, Japan

## Abstract

Xylem vessels function in the long-distance conduction of water in land plants. The NAC transcription factor VASCULAR-RELATED NAC-DOMAIN7 (VND7) is a master regulator of xylem vessel cell differentiation in Arabidopsis (*Arabidopsis thaliana*). We previously isolated *suppressor of ectopic xylem vessel cell differentiation induced by VND7* (*seiv*) mutants. Here, we report that the responsible genes for *seiv3*, *seiv4*, *seiv6*, and *seiv9* are protein ubiquitination-related genes encoding PLANT U-BOX46 (PUB46), an uncharacterized F-BOX protein (FBX), PUB36, and UBIQUITIN-SPECIFIC PROTEASE1 (UBP1), respectively. We also found decreased expression of genes downstream of VND7 and abnormal xylem transport activity in the *seiv* mutants. Upon VND7 induction, ubiquitination levels from 492 and 180 protein groups were upregulated and downregulated, respectively. VND7 induction resulted in the ubiquitination of proteins for cell wall biosynthesis and protein transport, whereas such active protein ubiquitination did not occur in the *seiv* mutants. We detected the ubiquitination of three lysine residues in VND7: K94, K105, and K260. Substituting K94 with arginine significantly decreased the transactivation activity of VND7, suggesting that the ubiquitination of K94 is crucial for regulating VND7 activity. Our findings highlight the crucial roles of target protein ubiquitination in regulating xylem vessel activity.

## Teaser

Suppressor mutant analysis and ubiquitome analysis elucidated the crucial roles of protein ubiquitination in the regulation of xylem vessel functionality in *Arabidopsis.*

## Introduction

Land plants have evolved a specialized vascular system in which xylem vessels play a major role in conducting water, nutrients, and small-molecular-weight molecules for long-distance signaling (Lucas et al. 2013). Xylem vessel cell differentiation is characterized by secondary cell wall (SCW) thickening and programed cell death (PCD) (Samuels et al. 2006; Lucas et al. 2013; Zhang et al. 2014). SCW deposition involves the biosynthesis of lignin, cellulose, and hemicellulose (Meents et al. 2018). Lignin, a phenolic polymer, is an important biomolecule for enhancing cell wall stiffness and facilitating plant resistance to biotic and abiotic stress (Fukuda 2004). During PCD, all intracellular components are eliminated in the cell, including the nucleus and cytoplasm, resulting in a hollow-tube structure that enables efficient water uptake (Fukuda 2004; Turner et al. 2007).

Multiple efforts have been made to elucidate the regulatory mechanisms of xylem vessel differentiation. One breakthrough was the identification of the master switches for xylem vessel cell differentiation, i.e. the plant-specific NAC (NAM/ATAF1,2/CUC2) transcription factors VASCULAR-RELATED NAC-DOMAINs (VNDs) (Kubo et al. 2005). VNDs are widely conserved among land plants, and VND genes are preferentially expressed in developing vascular tissues of vascular plants (Kubo et al. 2005; Yamaguchi et al. 2008; Nakano et al. 2015; Akiyoshi et al. 2020). Overexpressing *VND7* strongly promoted ectopic xylem vessel formation in Arabidopsis (*Arabidopsis thaliana*) via the transcriptional induction of its downstream genes (Yamaguchi et al. 2010a), including genes for cellulose synthesis (*IRREGULAR XYLEM5* [*IRX5*/*CesA4*], *IRX3*/*CesA7* and *IRX1*/*CesA8*), lignin biosynthesis enzymes (*CAFFEOYL COENZYME A ESTER O-METHYLTRANSFERASE7* [*CCoAOMT7*] and *IRX12*/*LACCASE4*), and cysteine protease for PCD (*XYLEM CYSTEINE PEPTIDASE1* [*XCP1*]). Conversely, repressing the transcriptional activity of VND7 severely affected xylem vessel formation (Yamaguchi et al. 2010b). These findings highlight the importance of VNDs in xylem vessel cell differentiation.

To explore regulatory factors underlying xylem vessel cell differentiation, we previously performed ethyl methanesulfonate (EMS)-mediated mutagenesis of transgenic Arabidopsis plants harboring the *VND7-VP16-GR* induction system, in which VND7 can be post-translationally activated via glucocorticoid treatment to induce the transdifferentiation of xylem vessel elements. From the mutagenized *VND7-VP16-GR* lines, we isolated suppressor mutants showing defects in ectopic xylem vessel cell differentiation and named them *suppressor of ectopic xylem vessel cell differentiation induced by VND7* (*seiv*). In the *seiv1* mutant, a recessive mutation in *S-NITROSOGLUTATHIONE REDUCTASE 1* (*GSNOR1*) resulted in the misregulation of protein *S*-nitrosylation (Kawabe et al. 2018). The suppression of ectopic xylem vessel formation in *seiv1* was attributed to the inhibitory effect of *S*-nitrosylation on the transactivation activity of VND7 (Kawabe et al. 2018). This finding provides insight into the protein post-translational modification (PTM)-mediated regulation of xylem vessel cell differentiation. Eight dominant *seiv* mutants (*seiv2* to *seiv9*) also showed repressed transdifferentiation of xylem vessel cells, especially in aboveground tissues (Pawittra et al. 2020). Consistently, the induction of genes downstream of VND7 was considerably inhibited in the shoots of these mutants (Pawittra et al. 2020).

In this study, we further investigated the molecular mechanisms of xylem vessel cell differentiation by examining the *seiv* mutants. Whole-genome resequencing analysis identified the responsible genes for *seiv3*, *seiv4*, *seiv6*, and *seiv9* as the genes encoding Plant U-box type E3 ubiquitin ligase (PUB) protein PUB46, UBIQUITIN-SPECIFIC PROTEASE 1 (UBP1), an uncharacterized F-box protein (FBX), and PUB36, respectively: these genes are all related to protein ubiquitination. Ubiquitinome analysis revealed a specific set of ubiquitination events during VND7-dependent xylem vessel cell differentiation; however, such ubiquitination dynamics were disturbed in the *seiv* mutants. Furthermore, we identified three lysine residues (K94, K105, and K260) of VND7 as the ubiquitination sites during xylem vessel cell differentiation; K94 plays an essential role in regulating the transactivation activity of VND7. Our findings provide insights into the role of protein ubiquitination in regulating xylem vessel cell differentiation.

## Results

### Identification of single-nucleotide polymorphisms (SNPs) in *seiv* mutants by whole-genome sequencing

**Figure 1.**
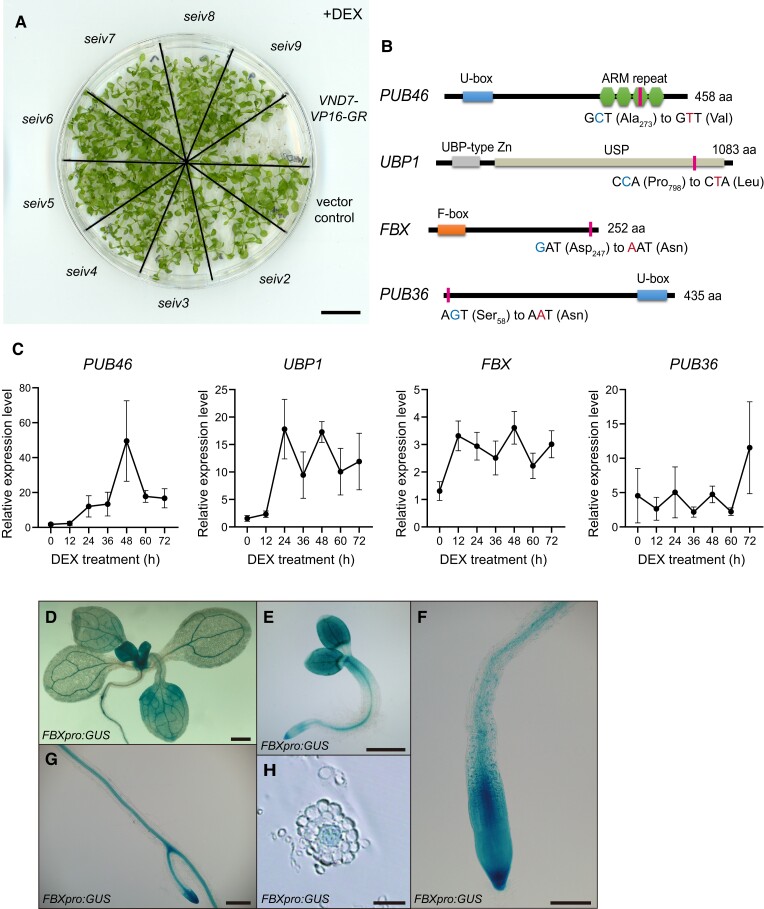
Ubiquitination-associated genes are responsible for the suppressed DEX-induced ectopic xylem vessel cell differentiation of the *seiv* mutants. **A)** Seven-day-old seedlings were treated with dexamethasone (DEX) for 4 d. Under DEX treatment, the *VND7-VP16-GR* seedlings became pale and died, while the *seiv* mutants survived. **B)** Validation of the candidate genes by Sanger sequencing confirms point mutations in *PUB46*, *UBP1*, *FBX*, and *PUB36*. The predicted amino acid substitution positions within the corresponding proteins are indicated by vertical lines. **C)** Expression analysis of *SEIV* genes upon dexamethasone (DEX) treatment. *VND7-VP16-GR* seedlings were treated with 10 *μ*M DEX and sampled every 12 h for RT-qPCR analysis. The expression levels of *PUB46*, *UBP1*, *FBX*, and *PUB36* were normalized to the internal control gene *ACTIN2*. Data are presented as the mean ± SD (*n* = 3). **D to H)** Expression patterns of the *FBX* promoter. Eleven-day-old **(D)** and 2-day-old **(E)** seedlings carrying *FBXpro:GUS*; magnified images of a lateral root **(G)** and root tip **(F)** from an 11-day-old *FBXpro:GUS* seedling. Transverse section of a root region of a 7-day-old seedling (H). Bars = 500 *μ*m (**E**), 200 *μ*m **(D, G)**, 100 *μ*m (**F**), and 50 *μ*m (**H**). At least 3 independent lines were assayed per construct.

In the *VND7-VP16-GR* inducible system, VND7 can be activated by treatment with the glucocorticoid dexamethasone (DEX), resulting in ectopic xylem vessel cell differentiation (Yamaguchi et al. 2010a). To isolate regulatory factors underlying VND7-dependent xylem vessel formation, we mutagenized Arabidopsis plants carrying the *VND7-VP16-GR* cassette via EMS treatment and looked for suppressor mutants (*seiv* mutants) (Kawabe et al. 2018; Ohtani and Demura 2019 ; Pawittra et al. 2020). A previous study on the recessive *seiv1* mutant uncovered an inhibitory effect of *S*-nitrosylation on the transcriptional activity of VND7, leading to a defect in ectopic xylem vessel cell differentiation (Kawabe et al. 2018). The dominant mutants *seiv2* to *seiv9* also exhibited similar suppression phenotypes (Fig. 1A; Pawittra et al. 2020).

To identify the underlying mutations responsible for the suppressed ectopic xylem vessel cell differentiation of the *seiv* mutants, we performed whole-genome sequencing and obtained lists of candidate genes. We ultimately identified a point mutation in AT5G18320 in *seiv3*, AT2G32780 in *seiv4*, AT5G39250 in *seiv6*, and AT3G61390 in *seiv9*, encoding PLANT U-BOX46 (PUB46), UBIQUITIN-SPECIFIC PROTEASE 1 (UBP1), an uncharacterized F-box protein (FBX), and PUB36, respectively (Fig. 1B). To verify that these point mutations cause suppressed DEX-induced ectopic xylem vessel cell differentiation in the *seiv* mutants, we introduced genomic fragments of *PUB46*, *UBP1*, *FBX*, and *PUB36* containing sequences 2 kb upstream and 0.5 kb downstream of the coding sequence with the corresponding point mutations into the wild-type *VND7-VP16-GR* background. The *VND7-VP16-GR* plants containing mutated *PUB46*, *UBP1*, *FBX*, and *PUB36* genes presented suppression phenotypes similar to those of the *seiv3*, *seiv4*, *seiv6*, and *seiv9* mutants, respectively: DEX treatment failed to induce ectopic xylem vessel cell differentiation (Supplementary Fig. S1). These results demonstrate that *PUB46*, *UBP1*, *FBX*, and *PUB36* are responsible for the phenotypes of *seiv3*, *seiv4*, *seiv6*, and *seiv9*, respectively.

We identified a C to T transition in the *seiv3* genome, leading to the substitution of Ala_273_ with Val in the third armadillo (ARM) repeat domain of PUB46. ARM repeats are short 42-amino acid motifs that mediate protein–protein interactions (Mudgil et al. 2004). Thus, the amino acid substitution (A273 V) in PUB46 conferred by the *seiv3* mutation may disturb the function of PUB46 or alter its substrates. In the *seiv4* genome, a single nucleotide substitution results in the substitution of Pro_798_ with Leu in the ubiquitin-specific protease catalytic domain of UBP1, which negatively affects its catalytic activity (Yan et al. 2000; Komander et al. 2009). The *seiv6* mutation changes Asp_247_ to Asn in FBX. The C-terminal region of FBX containing Asp_247_ might participate in protein–protein interactions and substrate recognition by FBX (Kuroda et al. 2002; Ogura et al. 2008), indicating that the function of FBX might be altered in the *seiv6* mutant. We also noticed that a point mutation (G to A) in *seiv9* causes the replacement of Ser_58_ by Asn in PUB36. Unfortunately, the impact of the *seiv9* mutation could not be determined because there is no clear description of the region containing Ser_58_ in PUB36. Since these *seiv* mutations are all dominant mutations (Pawittra et al. 2020), the dominant-negative effects of these ubiquitin-related genes suppress the VND7-based induction of xylem vessel cell differentiation.

### *SEIV* expression is closely associated with VND7-dependent xylem vessel formation

To gain additional insight into the roles of SEIV proteins in xylem vessel cell differentiation, we treated *VND7-VP16-GR* seedlings with 10 *µ*M DEX to induce xylem vessel cell differentiation and collected samples every 12 h until 72 h of treatment. We extracted total RNA from the samples and subjected it to RT-qPCR analysis to examine the expression of ubiquitination-related *SEIV* genes, including *PUB46* for *SEIV3*, *UBP1* for *SEIV4*, *FBX* for *SEIV6*, and *PUB36* for *SEIV9*. All *SEIV* genes were upregulated after DEX treatment, with peak expression at different time points (Fig. 1C; Supplementary Fig. S2G). *FBX* and *UBP1* were upregulated by 12 and 24 h of DEX treatment, respectively, i.e. during the early stage of xylem vessel differentiation, and their expression levels were maintained at relatively high levels. By contrast, *PUB46* and *PUB36* were upregulated at the later stages of xylem vessel cell differentiation, i.e. 48 and 72 h of treatment, respectively (Fig. 1C; Supplementary Fig. S2G). These results suggest that ubiquitination-related SEIV proteins play specific roles at certain stages of xylem vessel cell differentiation.

A previous study examined the tissue-specific expression of *PUB46* in vascular tissue in leaves, roots, and the stem–root transition zone (Adler et al. 2017). We investigated the spatial expression of *UBP1*, *FBX*, and *PUB36* by examining GUS expression under the control of their putative promoter sequences. Specifically, the plasmids containing GUS reporter driven by the upstream regions of *PUB46*, *UBP1*, *FBX*, and *PUB36* were transformed into the wild-type Arabidopsis. In transgenic plants carrying the *FBXpro:GUS* reporter, GUS activity was observed in vascular regions 2 d after germination (Fig. 1E). In seedlings grown for 4, 7, and 11 d, strong GUS signals were detected in both aboveground and underground vascular tissues, specifically the veins in leaves and xylem vessels in roots (Fig. 1, D, G and H). In addition, we detected strong GUS signals in *FBXpro:GUS* plants in shoot apical regions containing young leaves and in root apical regions, especially the root cap (Fig. 1F). By contrast, in transgenic plants carrying the *PUB36pro:GUS* and *UBP1pro:GUS* reporter genes, little GUS staining was observed in 1- to 3-d-old seedlings, but strong signals were detected in the underground tissues of 7-d-old seedlings. In transverse sections of roots, *PUB36* and *UBP1* promoter activity was high in the epidermis and cortex, as well as in the quiescent center of the root apex (Supplementary Fig. S2, A to F). Together, the differential expression patterns of *SEIV* genes would suggest the differential roles of each SEIV protein for xylem vessel formation.

### The *seiv* mutations affect xylem vessel formation

The dominant mutants *seiv3, seiv4*, *seiv6*, and *seiv9* showed defects in VND7-dependent ectopic xylem vessel cell differentiation (Fig. 1A). After DEX treatment, the leaves of the *seiv* mutants remained green, whereas the parental line *VND7-VP16-GR* became bleached owing to excessive ectopic xylem vessel cell differentiation. Specifically, DEX-induced ectopic xylem vessel cell formation was strongly repressed in aboveground tissues of the mutant, i.e. the cotyledons and hypocotyl. Remarkably, ectopic SCW formation could not be detected in the shoot apical regions of any of the *seiv* mutants (Pawittra et al. 2020). In the root tissue of *seiv* mutants, although the induction of SCW-related genes was slightly suppressed (Pawittra et al. 2020), ectopic xylem vessel cell differentiation still could be observed in response to DEX treatment (Supplementary Fig. S3). These observations indicate that the dominant *seiv* mutations preferentially affect VND7-induced xylem vessel cell differentiation in aboveground tissues.

**Figure 2.**
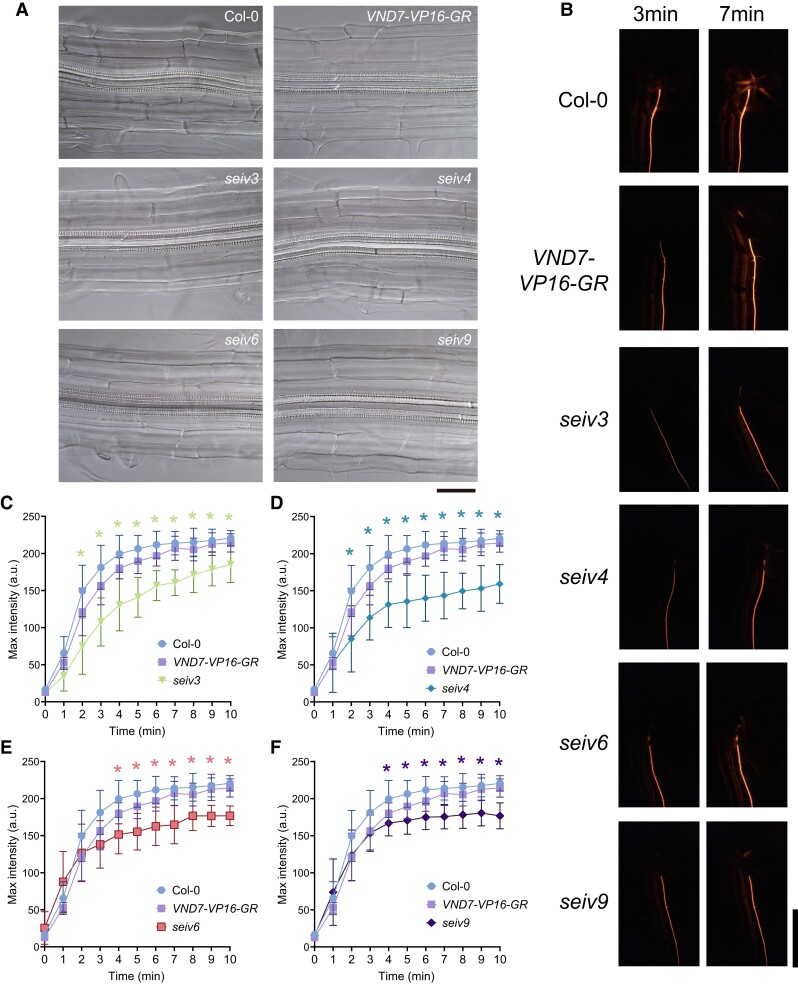
Effects of the *seiv* mutations on endogenous xylem vessel formation in primary roots. **A)** Microscopy of the root xylem tissues of 7-day-old Col-0, *VND7-VP16-GR*, *seiv3, seiv4*, *seiv6*, and *seiv9* seedlings. Bar = 50 *µ*m. **B)** The fluorescent dye rhodamine was used to stain the excised tips of primary roots of 14-day-old Col-0, wild-type *VND7-VP16-GR*, and *seiv* seedlings. The fluorescent signal was observed every minute and recorded. Bar = 1 cm. **C to F)** Quantitative data of fluorescence intensity (arbitrary units) in Col-0, wild-type *VND7-VP16-GR*, and *seiv* tissues. Data are presented as the mean ± SE. *n* = 9 (Col-0), 5 (*VND7-VP16-GR*), 9 (*seiv3*), 8 (*seiv4*), 8 (*seiv6*), 8 (*seiv9*). Asterisks indicate significant difference between *seiv* and wild-type *VND7-VP16-GR* (LSD test, *P* < 0.01).

To examine the impacts of the *seiv* mutations on endogenous xylem vessel cell differentiation, we examined the xylem vessel cell differentiation in the *seiv* mutants in the absence of DEX treatment by microscopy observation. We did not detect any clear visible differences between Col-0, wild-type *VND7-VP16-GR*, and the *seiv* mutants in the cotyledons, hypocotyls, leave veins, and primary roots (Supplementary Figs. S4 to S6; Fig. 2A); microscopic analysis did not show any difference in the number or size of xylem vessels or in SCW deposition between the control plants and *seiv* mutants (Fig. 2A).

While no substantial morphological difference was observed in xylem vessel formation and SCW thickenings between *seiv* mutants and control groups in the absence of DEX treatment, we cannot deny the possibility that the physicochemical properties of xylem cell walls in *seiv* mutant were changed. Particularly, some alternations in cell wall composition and structure are hard to figure out via microscopy observation. In the previous study (Endo et al. 2019), several transgenic lines with distinct modifications of xylem cell walls but no visible defects in vascular tissues presented reduced or accumulated transport efficiency, which demonstrated a tight association between the structures of xylem cell walls and xylem transport patterns. Thus, we also performed this xylem transport assay to check whether the cell wall properties in *seiv* mutants are altered or not. Considering the growth variations in the aboveground tissues, and observing xylem vessels in primary roots is more operable and comparable, we checked the fluorescence transport ability in primary roots in each line. We applied the water-soluble fluorescent dye rhodamine to excised root tips of Col-0, wild-type *VND7-VP16-GR*, and the *seiv* mutants and observed the transport of rhodamine to the aboveground parts of the plants by recording the fluorescent signals (Fig. 2B, Supplementary Fig. S7). In Col-0, the fluorescent signals reached the aboveground parts of the plants within 1 min and began to spread to the leaf veins within 5 min (Fig. 2B, Supplementary Fig. S7). No significant difference in the movement or intensity of fluorescent signals was observed between wild-type *VND7-VP16-GR* and Col-0 plants (Fig. 2B, Supplementary Fig. S7; Supplementary Data Set 1), indicating that the *VND7-VP16-GR* system did not affect xylem transport under normal conditions.

By contrast, the dynamics of fluorescent signal transport were affected in the *seiv* mutants. Two types of abnormalities were observed. First, the speed at which the dye was transported in the root region within 3 min, i.e. before the signals reached a plateau, was lower in *seiv3* and *seiv4* than in Col-0 or wild-type *VND7-VP16-GR* (Fig. 2, C and D; Supplementary Data Set 1). Second, the maximum signal intensity after the signals reached a plateau was lower in *seiv3, seiv4*, *seiv6*, and *seiv9* than in Col-0 or wild-type *VND7-VP16-GR* (Fig. 2, C to F; Supplementary Data Set 1). The former is related to the transport velocity through xylem vessels, and the latter to the maximum transport volume of xylem vessels. Moreover, the fluorescence signals in aboveground tissues were not clearly visible in the *seiv* mutants (Supplementary Fig. S7; Supplementary Data Set 1). This observation is consistent with the finding that the severe inhibitory effects of the *seiv* mutations on ectopic xylem vessel differentiation are found in aboveground tissues but not belowground tissues (Pawittra et al. 2020). These results indicate that the transport capacities of xylem vessels are impaired in the *seiv* mutants, even though the xylem vessels appear to be morphologically normal, and that SEIV proteins are important for regulating xylem vessel functionality.

### The *seiv* mutations affect DEX-induced transcriptomic changes

**Figure 3.**
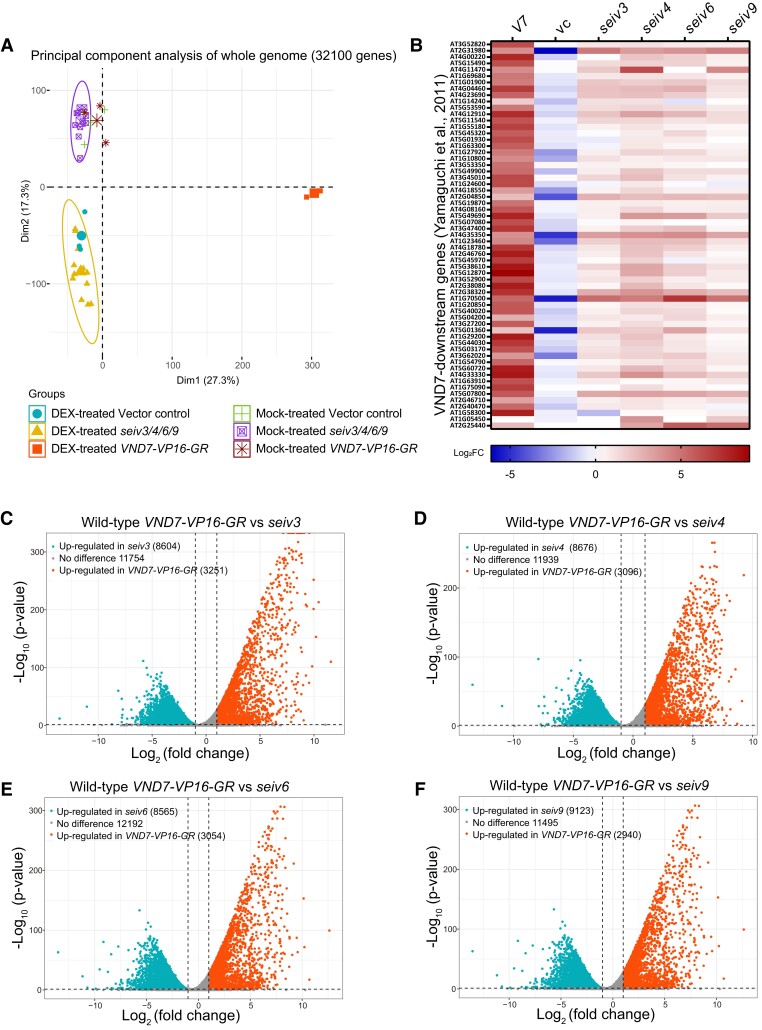
Transcriptional changes in the *seiv* mutants. Seven-day-old seedlings were treated with DEX and collected after 6 h of treatment. Total RNA was extracted from the samples and subjected to RNA-seq analysis. Three replicates were analyzed per treatment condition. **A)** Principal component analysis (PCA) of whole transcriptome data. **B)** Expression levels of VND7-downstream genes in the *seiv* mutants. The VND7-downstream genes were selected from the RNA-Seq data from wild-type *VND7-VP16-GR* (*V7*), the vector control (vc), and *seiv3*, *seiv4*, *seiv6*, and *seiv9* mutants treated with DEX based on Yamaguchi et al. (2011) and used to construct the heatmap. **(C to F)** Comparison of the transcriptome profiles of *seiv3***(A)**, *seiv4***(B)**, *seiv6***(C)**, and *seiv9***(D)** to that of wild-type *VND7-VP16-GR* with DEX treatment. Volcano plots of the fold change and *P*-value of each gene between wild-type *VND7-VP16-GR* and *seiv*. The dotted lines on the right and left indicate thresholds at which gene expression was determined to be significantly upregulated in wild-type *VND7-VP16-GR* or *seiv* mutants, respectively.

To obtain a global view of the transcriptional changes in the *seiv* mutants, we treated 7-d-old vector control, wild-type *VND7-VP16-GR*, and *seiv* mutant seedlings with DEX or DMSO (mock) for 6 h and performed RNA-seq analysis. Principal component analysis (PCA) of gene expression levels genome-wide revealed clear separation of DEX- and mock-treated samples, depicting the distinct transcriptomic changes caused by the *seiv* mutations (Fig. 3A). The first two principal components explained 44.6% of the total variance, with the first component explaining 27.3% and the second component explaining 17.3% (Dim1 and Dim2 in Fig. 3A). The biplot showed that both the DEX-treated and mock-treated *seiv* mutant groups were not strongly associated with the first principal component. By contrast, the DEX-treated *VND7-VP16-GR* lines were strongly associated with the positive direction of the first principal component (Dim1 in Fig. 3A), and GO term analysis revealed a significant enrichment of cell wall biosynthesis-related events in the first component (Supplementary Data Set 2), suggesting that the first component is highly related to VND7-dependent xylem vessel differentiation.

Thus, we compared the gene expression levels between wild-type *VND7-VP16-GR* and *seiv* plants under DEX treatment and identified differentially expressed genes (DEGs) based on the criteria log_2_FC ≥1 and q-value <0.05. More than 8,500 genes were significantly upregulated in the *seiv* mutants and approximately 3,000 were significantly upregulated in wild-type *VND7-VP16-GR* (Fig. 3, C to F). Gene Ontology (GO) enrichment analysis revealed that the significantly enriched GO terms in the upregulated genes of wild-type *VND7-VP16-GR*—“Golgi vesicle transport”, “plant-type cell wall biogenesis”, “plant-type secondary cell wall biogenesis”, “endoplasmic reticulum to Golgi vesicle-mediated transport”, and “cell wall macromolecule metabolic process” (Supplementary Data Sets 3 and 4)—were strongly downregulated in *seiv3*, *seiv4*, *seiv6*, and *seiv9* (Supplementary Fig. S8; Supplementary Data Sets 5 to 12), which is consistent with the suppressed DEX-induced ectopic xylem vessel cell differentiation in the *seiv* mutants. Moreover, we measured the expression levels of 61 genes encoding proteins that function downstream of VND7 reported in Yamaguchi et al. (2010a, 2011) in the *seiv* mutants. These genes were upregulated by DEX treatment in the *seiv* mutants, but their expression levels were considerably reduced (Fig. 3B). GO term analysis displayed different patterns in the upregulated and downregulated genes in *seiv* mutants (Supplementary Figs. S8 and S9), indicating *seiv* mutations perform distinct impacts while they exhibited similar suppression phenomenon in ectopic xylem vessel cell differentiation.

Besides, we also checked the transcriptome changes in *seiv* mutants under mock treatment. GO terms associated with “plant-type secondary cell wall biogenesis”, “regulation of cell wall organization or biogenesis”, and other SCW-related events were also enriched in the downregulated genes among *seiv* mutants (Supplementary Fig. S10). VND7-downstream genes showed reduced expression levels in the absence of DEX treatment (Supplementary Fig. S11). These findings strongly suggest that *seiv* mutation would impair VND7-dependent endogenous xylem vessel formation, corresponding to the altered transport capacities of xylem vessels in the *seiv* mutants (Fig. 2, C to F). Overall, our RNA-seq data highlights the unique effects of the *seiv* mutations on transcriptomic changes in response to ectopic and endogenous xylem vessel formation.

When we picked up the upregulated genes in wild-type *VND7-VP16-GR* and performed PCA with these genes, the clear separation of DEX-treated wild-type *VND7-VP16-GR* samples from other samples were commonly observed (Fig. 3A; Supplementary Fig. S12A). However, the PCA plots of second component (Dim2) and other components (Dim3/4/5) indicated that the *seiv* mutants showed different distributions from each other (Supplementary Fig. S12, C to E), which indicated potential differential impacts of *seiv* mutations on transcriptome dynamics upon the DEX treatment. Indeed, the GO enrichment analysis for the genes that contributed to second to fifth components showed the variation of gene functions of each component, suggesting potentially differentiated impacts of *seiv* mutations to xylem vessel cell differentiation (Supplementary Data Sets 13 to 16).

### Protein ubiquitination dynamics in response to VND7 induction

**Figure 4.**
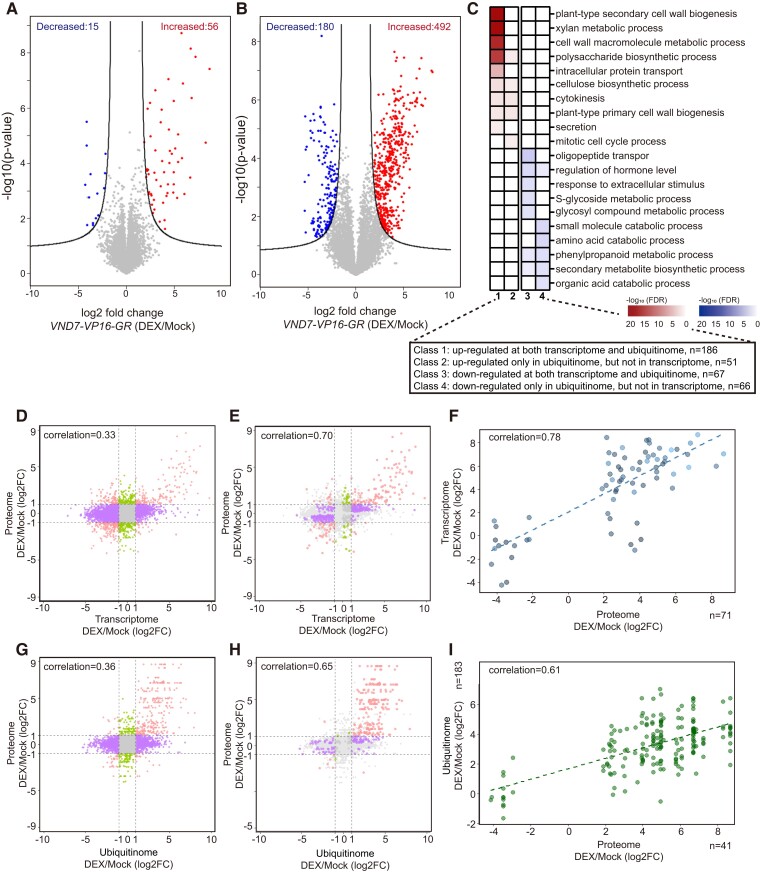
Proteome and ubiquitinome profiling of xylem vessel cell differentiation. **A)** Proteome profiles during xylem vessel cell differentiation. The volcano plot highlights changes in protein abundance following VND7 induction identified in wild-type *VND7-VP16-GR*. Individual peptides were plotted based on log_2_FC values and statistical significance (−log_10_*P*-value). Peptides with significantly increased or decreased abundance are highlighted (FDR of 0.05 and an S0 of 1.0). **B)** Ubiquitinome dynamics during xylem vessel cell differentiation. The volcano plot highlights changes in ubiquitination following VND7 induction identified in wild-type *VND7-VP16-GR*. Individual ubiquitinated sites were plotted based on log2FC and statistical significance (−log_10_*P*-value). Ubiquitinated sites with significantly increased or decreased abundance are highlighted (FDR of 0.05 and an S0 of 1.0). **C)** Distribution of differentially regulated ubiquitinated proteins of wild-type *VND7-VP16-GR* during xylem vessel cell differentiation. Ubiquitinated proteins whose ubiquitination level was altered by VND7 induction in wild-type *VND7-VP16-GR* were categorized by gene expression level based on the transcriptome data. Class 1: upregulated in both the transcriptome and ubiquitinome, *n* = 186; Class 2: upregulated in the ubiquitinome, but not the transcriptome, *n* = 51; Class 3: downregulated in both the transcriptome and ubiquitinome, *n* = 67; Class 4: downregulated in the ubiquitinome, but not the transcriptome, *n* = 66. The significance of the selected GO terms in four classes are shown in the heatmap. **D to F)** Correlation between the transcriptome and proteome data. Scatter plot of 9-quadrant association analysis of mRNA and proteins based on log_2_FC (DEX/Mock); log_2_FC ≥ 1 (**D**); log_2_FC ≥ 1, *P*-value < 0.05 **(E)**. Fold changes of significant differentially expressed proteins (*n* = 71) and their corresponding genes relative to mock treatment were subjected to analysis **(F)**. **G to I)** Correlation between the ubiquitinome and proteome data. Scatter plot of 9-quadrant association analysis of ubiquitinated sites and proteins from log_2_FC (DEX/Mock); log_2_FC ≥ 1 **(G)**; log_2_FC ≥ 1, *P*-value < 0.05 **(H)**. Fold changes of significant differentially expressed proteins (*n* = 41) and their corresponding ubiquitinated sites (*n* = 183) relative to mock treatment were subjected to analysis **(I)**.

We profiled the dynamics of both the proteome and ubiquitinome of the vector control, wild-type *VND7-VP16-GR*, *seiv6*, and *seiv9*. We extracted proteins from seedlings that were mock- or DEX-treated for 6 h and subjected them to LC-MS/MS analysis. We detected and quantified 6980 proteins in all genotypes, 56 of which were significantly accumulated and 15 of which were reduced in wild-type *VND7-VP16-GR* upon DEX treatment, respectively (Fig. 4A, Supplementary Data Sets 17 and 18). GO analysis revealed that the abundance of cell-wall-related proteins significantly increased following VND7 induction (Supplementary Data Set 19), including proteins related to SCW biosynthesis (CESA4, CESA8, IRX12/LAC4, and FLA11) and PCD (XCP2 and SCPL20) (Gardiner et al. 2003; Taylor et al. 2003; Avci et al. 2008; MacMillan et al. 2010; Berthet et al. 2011; Schuetz et al. 2014; Olvera-Carrillo et al. 2015), as shown in previous proteome studies (Noguchi et al. 2018; Arae et al. 2022).

**Figure 5.**
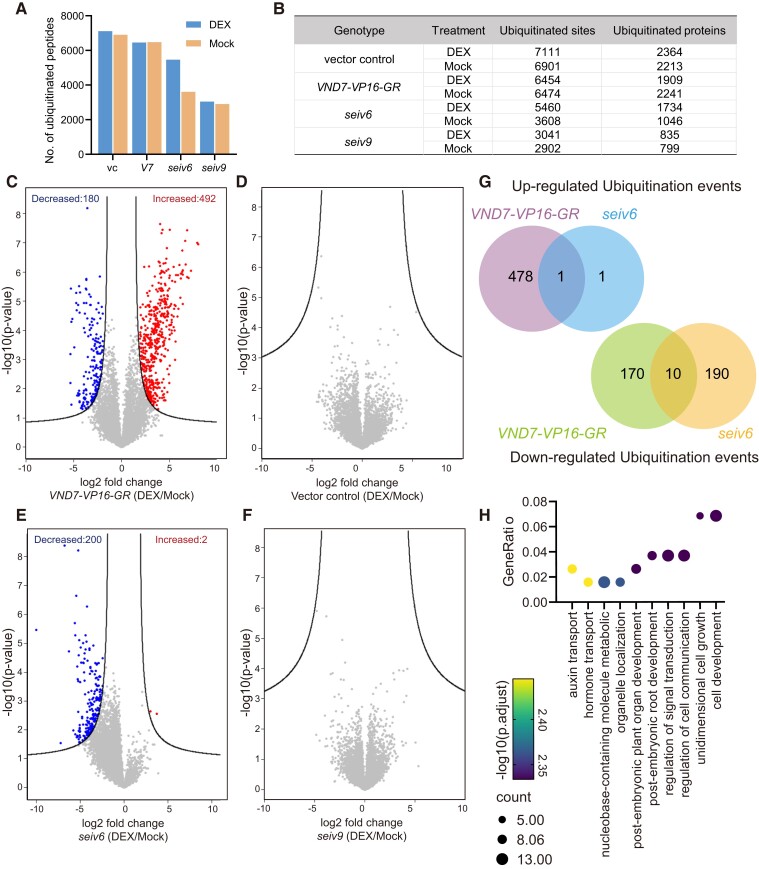
Ubiquitinome dynamics are disturbed in *seiv6* and *seiv9*. **A)** Distribution of ubiquitinated sites in the vector control, wild-type *VND7-VP16-GR*, *seiv6*, and *seiv9* following treatment with DEX or DMSO (Mock). **B)** Abundance of ubiquitinated sites and ubiquitinated proteins identified in each sample. **C to F)** Volcano plots highlighting changes in ubiquitination following VND7 induction identified in wild-type *VND7-VP16-GR*, the vector control, *seiv6*, and *seiv9*. Individual ubiquitinated sites were plotted by log2FC values and statistical significance (−log_10_*P*-value). Ubiquitinated sites with significantly increased or decreased accumulation are highlighted (FDR of 0.05 and an S0 of 1.0). **G)** Venn diagrams showing the common and specific upregulated and downregulated ubiquitination events in response to VND7 induction, respectively. **H)** Over-represented complete GO terms (Biological process) for significantly downregulated ubiquitination events in *seiv6* upon DEX treatment.

We performed affinity enrichment of ubiquitinated tryptic peptides with a specific K-ε-GG antibody, which recognizes a remnant diGly tag on a formerly ubiquitinated lysine residue (Udeshi et al. 2013), using the vector control, wild-type *VND7-VP16-GR*, *seiv6*, and *seiv9*. We identified 9,809 ubiquitination sites in 3,476 protein groups from all samples, more than 64% of which had not been reported previously in Arabidopsis (Fig. 5, A and B; Kim et al. 2013; Aguilar-Hernández et al. 2017). In total, 6,474 ubiquitinated sites in 2,241 proteins were detected in wild-type *VND7-VP16-GR* after DEX treatment, suggesting that ubiquitination plays an important role in xylem vessel cell differentiation, which is consistent with previous findings (Stephenson et al. 1996; Woffenden et al. 1998; Endo et al. 2001; Jin et al. 2006; Ibañes et al. 2009). Comparative analysis between mock- and DEX-treated *VND7-VP16-GR* revealed 492 sites with significantly increased levels of ubiquitination and 180 with significantly decreased levels of ubiquitination after VND7 induction (Fig. 4B; log2FC ≥1; *P*-value <0.05), highlighting the specific ubiquitination that occurs during xylem vessel cell differentiation.

Based on these omics data, we examined the correlations between transcript, ubiquitination, and protein profiles in response to VND7 induction. Significant differentially expressed proteins and transcripts are mainly distributed in quadrant 1 in the graphs in Fig. 4, D and E, indicating a tight association between transcript and protein levels, especially those whose expression levels were significantly elevated upon VND7 induction (Fig. 4, D and E). We observed a similar distribution pattern in correlation analysis of the ubiquitinome and proteome (Fig. 4, G and H). Correlation analysis showed a strong positive correlation (*r*^2^ = 0.78) between the transcriptome and proteome data (Fig. 4F) and a moderate positive correlation (*r*^2^ = 0.61) between the ubiquitinome and proteome data (Fig. 4I) for the 71 differentially expressed proteins (Supplementary Data Sets 17 and 18). Accordingly, integrated omics analysis indicated that the changes in protein ubiquitination are associated with changes in transcript levels and (consequently) protein levels.

To further investigate the importance of protein ubiquitination in xylem vessel cell differentiation, we focused on proteins whose ubiquitination levels were significantly altered upon VND7 induction. Accordingly, we isolated differentially ubiquitinated proteins and categorized them based on the transcriptome and ubiquitinome data as follows: Class 1, proteins that were upregulated in both the transcriptome and ubiquitinome; Class 2, proteins that were upregulated only in the ubiquitinome; Class 3, proteins that were downregulated in both the transcriptome and ubiquitinome; and Class 4, proteins that were downregulated only in the ubiquitinome.

GO enrichment analysis revealed that cell-wall-related proteins in Class 1 considerably accumulated upon VND7 induction (Fig. 4C; Supplementary Data Set 20), including SCW-type CESAs (CESA4, CESA7, CESA8; Gardiner et al. 2003; Taylor et al. 2003), xylan biosynthesis enzymes (IRX6, IRX8, IRX9; Brown et al. 2005; Persson et al. 2007; Wu et al. 2010), xylan acetylation enzymes (REDUCED WALL ACETYLATE1 [RWA1], RWA3, RWA4; Lee et al. 2011; Manabe et al. 2013), and LAC4, which contributes to lignin biosynthesis (Berthet et al. 2011; Schuetz et al. 2014). Considering the transcriptional changes following VND7 induction described previously (Yamaguchi et al. 2011), the increased ubiquitination of proteins in Class 1 could be attributed to transcriptional upregulation that is dependent on VND7. Class 2 includes CESA1, CESA2, and CESA10, which are involved in PCW cellulose biosynthesis (Persson et al. 2005, 2007; Desprez et al. 2007), and proteins related to protein transport and localization, i.e. the v-SNARE family protein VESICLE-ASSOCIATED MEMBRANE PROTEIN713 (VAMP713; Ebine et al. 2014) and Qa-SNARE protein SYNTAXIN OF PLANTS123 (SYP123; Fujiwara et al. 2014). These proteins are likely to be specific targets of ubiquitination during xylem vessel cell differentiation (Fig. 4C; Supplementary Data Set 21). In addition, the proteins belonging to Class 3 and 4 share common GO terms, such as “regulation of hormone levels”, “secondary metabolite biosynthetic process”, and “phenylpropanoid metabolic process” (Fig. 4C; Supplementary Data Sets 22 and 23). These results imply that the ubiquitination of proteins for these molecular functions decreases concurrently with and/or independently of the downregulation of the corresponding genes.

In summary, our omics data analysis reveals important insights into the dynamics of protein and ubiquitinome profiles during xylem vessel cell differentiation. We found a tight correlation between transcript, ubiquitinome, and proteome profiles, particularly in proteins upregulated upon VND7 induction. Moreover, our results highlight the crucial role of ubiquitination in regulating cell wall-related processes and protein transport during this differentiation process.

### Ubiquitinome dynamics are perturbed in *seiv6* and *seiv9*

Unexpectedly, in comparative analysis of the protein and ubiquitinome profiles derived from *seiv6* and *seiv9*, we only identified 3,680 ubiquitinated sites in 1,024 proteins in *seiv6* and 2,902 ubiquitinated sites in 799 proteins in *seiv9* (Fig. 5, A and B). The reduced ubiquitination in *seiv6* and *seiv9* strongly supports the hypothesis that FBX^D247N^ and PUB36^S58N^ disturb the regulation of protein ubiquitination in plants.

Interestingly, this ubiquitination dynamic was disturbed in *seiv6* and *seiv9*: DEX-induced accumulation of ubiquitination almost disappeared in *seiv6* and *seiv9* (Fig. 5, E, F, and G), and surprisingly, there was an abnormal abundance of sites with reduced ubiquitination in *seiv6* upon DEX treatment (Fig. 5, E, G and H; Supplementary Fig. S13; Supplementary Data Sets 24 and 25). These findings demonstrate that a specific set of ubiquitination events that normally occur during xylem vessel cell differentiation failed to be induced by DEX treatment in the *seiv* mutants. We also discovered considerable differences in the number of ubiquitinated sites in *seiv9* even in the absence of DEX treatment (Fig. 5, A and B). The reduced ubiquitination in *seiv9* regardless of VND7 induction suggests that the *seiv9* mutation strongly affects the ubiquitination profile under normal growth conditions. Thus, the *seiv9* mutant cannot respond to DEX treatment, as shown by the low number of differentially ubiquitinated proteins after VND7 induction in *seiv9* (Fig. 5B).

### VND7 can be ubiquitinated at three residues

**Figure 6.**
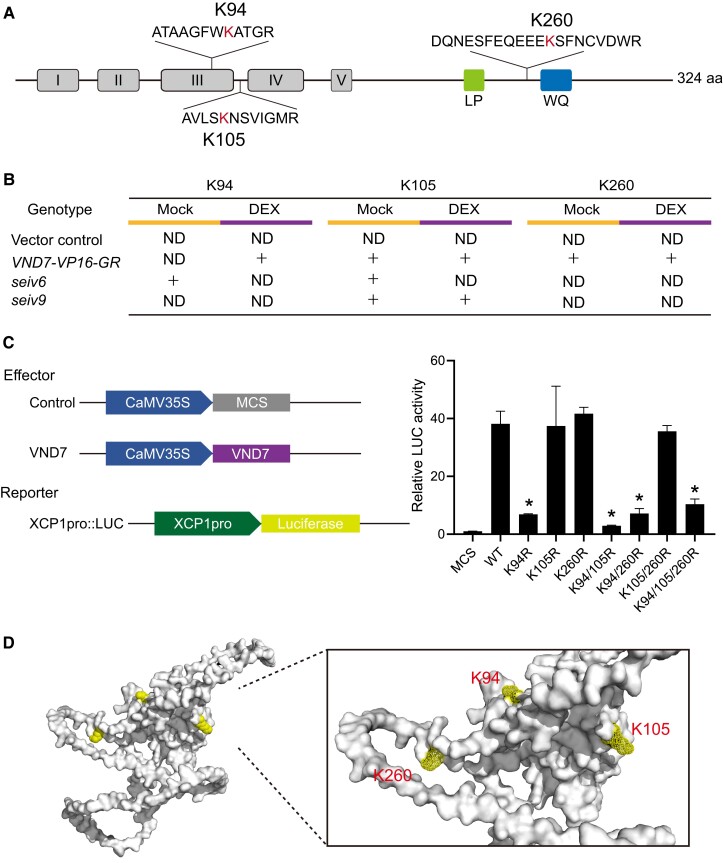
The importance of ubiquitination for the transactivation activity of VND7. **A)** Diagram of VND7 showing the identified ubiquitination sites relative to the NAC domains and the LP and WQ box. The MS-identified peptide sequence bearing each ubiquitin footprint with the modified lysine is shown. **B)** Detection of ubiquitinated lysine residues in the vector control, wild-type *VND7-VP16-GR*, *seiv6*, and *seiv9* under DEX- and mock-treated conditions. “ND” indicates no detection of ubiquitination events, and “+” indicates the detection of ubiquitinated events. **C)** Schematic diagrams of the effector and reporter constructs used for the transient gene expression assays (left). The effector construct consists of DNA sequences encoding the full-length wild-type VND7 (WT) or mutated VND7 (K94R, K105R, K260R, K94R/K105R, K94R/K260R, K105R/K260R, K94R/K105R/K260R) under the control of the CaMV 35S promoter. The multi cloning site (MCS) sequence fused with the CaMV 35S promoter was used as a transfection control. The reporter construct consists of a luciferase (LUC) gene driven by the *XCP1* promoter fragment X1E1 (the promoter region at −148 to −96 bp; Yamaguchi et al. 2011). Transient reporter assays to elucidate the effects of mutations at Lys94, Lys105, and Lys260 of VND7 (right). All LUC activities were normalized to Rluc activity. Data are mean values ± SE (*n* = 3). The results are shown relative to the control effector (MCS = 1.0). Asterisks indicate significant differences (Student's *t*-test; *P*-value < 0.01) from wild-type VND7 (WT). **D)** Structure of VND7, predicted by AlphaFold (https://alphafold.ebi.ac.UK). The ubiquitinated lysine residues detected upon VND7 induction are highlighted.

Finally, we obtained evidence that VND7 can undergo ubiquitination, as expected based on the possible proteasome-mediated degradation of VND7 described in Yamaguchi et al. (2008). VND7 possesses 15 lysine residues, and K94, K105, and K260 were identified as the ubiquitin conjugation sites in our ubiquitinome data (Fig. 6A, Supplementary Fig. S14). In *VND7-VP16-GR* plants, these three lysine residues were ubiquitinated, whereas none of them were ubiquitinated in the vector control (Fig. 6B), suggesting that the ubiquitination of these residues plays a role in xylem vessel cell differentiation. Notably, K105 and K260 could be ubiquitinated in both mock- and DEX-treated *VND7-VP16-GR*, whereas ubiquitination of the K94 residue, which is located in the conserved NAC domain of VND7, was only detected after DEX treatment (Fig. 6B). These ubiquitination patterns were disturbed in *seiv6* and *seiv9*, as the ubiquitination of K260 disappeared in both *seiv6* and *seiv9*, K105 failed to be ubiquitinated in *seiv6* after DEX treatment, and VND7-induced ubiquitination of K94 was abolished in both *seiv6* and *seiv9* (Fig. 6B). Accordingly, we reasoned that the defects in VND7-dependent xylem vessel cell differentiation in *seiv6* and *seiv9* might be attributed to the misregulation of VND7 ubiquitination.

Therefore, we examined the impacts of the ubiquitination of K94, K105, and K260 on the transcriptional activation activity of VND7 by performing a transient reporter assay (Fig. 6C). For the reporter, we used a chimeric gene comprising the firefly luciferase gene fused to the X1E1 element in the *XCP1* promoter (−148 to −96 bp promoter region), which was verified to be directly recognized by VND7 (Yamaguchi et al. 2011; Tamura et al. 2019) (Fig. 6C). For the effectors, we used the full-length coding region of wild-type VND7 or mutagenized VND7 with single, double, and triple amino acid substitutions of K94, K105, and/or K260 with Arg (referred to as K94R, K105R, K260R, K94R/K105R, K94R/K260R, K105R/K260R, and K94R/K105R/K260R) driven by the *CaMV 35S* promoter (Fig. 6C). We introduced the constructs into protoplasts derived from Arabidopsis T87 suspension-culture cells along with a vector harboring *35S:RLUC* for normalization.

The strong luciferase activity in the presence of VND7 was significantly reduced when K94 was substituted with Arg; this mutation was expected to prevent ubiquitination (Fig. 6C; Supplementary Fig. S15). VND7^K94R/K105R^, VND7^K94R/K260R^, and VND7^K94R/K105R/K260R^ also showed considerably reduced transactivation activity compared to VND7, whereas the substitution of Lys to Arg at K105 and K260 did not affect luciferase activity (Fig. 6C). These results indicate that the ubiquitination of K94 plays a critical role in regulating the transactivation activity of VND7.

Furthermore, the above findings raise the question of whether VND7 serves as a substrate for SEIV proteins. To investigate this possibility, we conducted a yeast 2-hybrid (Y2H) assay to assess the interaction between VND7 and SEIV proteins. However, our results revealed no direct interaction between them (Supplementary Fig. S16), suggesting that SEIV proteins do not regulate VND7 via ubiquitination or deubiquitylation.

## Discussion

### The responsible genes for *seiv3*, *seiv4*, *seiv6*, and *seiv9* are related to protein ubiquitination

In this study, we determined that the responsible genes for *seiv3*, *seiv4*, *seiv6*, and *seiv9* are the ubiquitination-related genes *PUB46*, *UBP1*, *FBX*, and *PUB36*, respectively (Fig. 1B). PUB46, FBX, and PUB36 function as E3 ligases for the attachment of ubiquitin, while UBP1 is capable of cleaving ubiquitin. *PUB46* and *PUB36* encode U-BOX-containing E3 ligases belonging to the PLANT U-BOX (PUB) family. In addition to the conserved U-box domain, PUB46 possesses C-terminal Armadillo (ARM) repeats that mediate protein–protein interactions. PUBs harboring ARMs have drawn attention for their association with stress responses in plants (Trenner et al. 2022); for example, Arabidopsis mutants of PUB22, PUB23, and PUB24 display enhanced immune responses and enhanced tolerance against pathogens (Trujillo et al. 2008). *AtPUB46* and its paralogous gene *AtPUB48* are implicated in the plant response to water stress (Adler et al. 2017). Unlike PUB46, PUB36 only possesses a C-terminal U-box domain, which binds to E2 ligases. Thus, the impact of the S58N mutation on the function of PUB36 is unclear. In addition to PUBs, the SCF (SKP-cullin-F-box protein) complex is one of major E3 ligases (Hershko and Ciechanover 1998; Thompson et al. 2021). F-box proteins interact with SKP1 via their F-box regions and with target proteins via their C-terminal substrate-binding domains, which determine substrate specificity (Craig and Tyers 1999; Lechner et al. 2006). Thus, the D247N mutation in the C terminus of FBX likely affects protein–protein interactions or substrate recognition. UBP1 is a deubiquitinating enzyme (DUB) that releases ubiquitin from its substrates. UBPs are cysteine proteases characterized by two conserved catalytic motifs (Cys- and His-box) and universal stress protein domains (Yan et al. 2000; Komander et al. 2009) The universal stress protein domain can be disrupted by a large polypeptide insertion, affecting its enzymatic ability (Reyes-Turcu et al. 2008; Ye et al. 2009). Thus, we reasoned that the P798L amino acid substitution in the catalytic core might negatively affect the function of UBP1.

Therefore, we suggest that point mutations in *seiv3*, *seiv4*, *seiv6*, and *seiv9* affect the functions of PUB46, UBP1, FBX and PUB36, respectively. Our ubiquitinome data indicate that ubiquitination dynamics were indeed disturbed in *seiv6* and *seiv9*; significantly fewer ubiquitination events were detected in both *seiv6* and *seiv9*, suggesting that FBX and PUB36 modulate ubiquitination dynamics. Additionally, considering that the *seiv6* and *seiv9* mutants are dominant mutants (Pawittra et al. 2020), it is reasonable to expect that FBX^D247N^ and PUB36^S58N^ have gain-of-function effects on proteins, such as their activity, stability, interactions, and subcellular localizations (Meinke 2013). Therefore, even though the effects of knocking out *PUB36* or *FBX* would be masked by redundancy in this large multigene family, it would still be possible to perform functional analysis based on these dominant-negative effects.

### Protein ubiquitination is a crucial regulatory molecular layer of xylem vessel cell differentiation

The *seiv* mutants show defects in ectopic xylem vessel cell differentiation upon DEX treatment, especially in aboveground tissues (Pawittra et al. 2020; Fig. 1A). Although we detected no obvious defects in morphology or growth in the *seiv* mutants under normal growth conditions, the xylem transport assay revealed abnormal transport dynamics in the *seiv* mutants (Fig. 2, B and C). Together with transcriptome data analysis indicating that genes for xylem vessel formation were abnormally expressed in *seiv* seedlings (Supplementary Figs. S10 and S11), it can be suggested that alternations in cell wall composition and structure would be caused by *seiv* mutations. The ubiquitinome data demonstrate that protein ubiquitination was abnormal in the *seiv* mutant plants, even under mock treatment (Fig. 5). Thus, we suggest that the misregulation of protein ubiquitination mediated by the *seiv* mutations disturbs the proper progression of xylem vessel cell differentiation, leading to defects in xylem vessel functionality. Together with previous findings on a group of genes encoding RING-H2 E3 ligases, involved in wood formation in *Populus trichocarpa* (Tong et al. 2019), and the RING-H2 E3 ligase ATL54, involved in SCW formation (Noda et al. 2013), the crucial role of protein ubiquitination in xylem vessel cell differentiation is now established. Currently it is difficult to examine effects of *seiv* mutations on physicochemical aspects of xylem cell walls due to the limited tool available at cellular resolution. In addition, we could not obtain additional genetic materials on any *SEIV* gene loci. As the *seiv* mutants obtained in our screening are dominant, thus the genetic complementation experiments are impossible to be performed for the *seiv* phenotypes defective for xylem transport activity (Fig. 2, B and C). These points should be tested with new techniques and/or new mutant materials, for further analysis of protein ubiquitination in xylem vessel cell differentiation.

Analysis using the tracheary element induction system in *Zinnia elegans* indicated that the 26S proteasome, which recognizes K48-linked ubiquitination for protein degradation (van Nocker and Vierstra 1993; Smalle and Vierstra 2004), preferentially accumulates in developing xylem vessel cells. Treatment with the proteasome inhibitors MG132 and lactacystin significantly delayed cell differentiation (Woffenden et al. 1998; Endo et al. 2001). Thus, one important function of protein ubiquitination is to target a protein for degradation, which shapes the cellular proteome for the progress of xylem vessel cell differentiation.

What are the targets of protein ubiquitination during xylem vessel cell differentiation? Our ubiquitinome analysis successfully detected a group of ubiquitination events specifically activated in response to VND7 induction, such as SCW biosynthetic enzymes (CESAs, IRXs, and LAC4), proteases related to PCD (XCP1 and XCP2), and factors related to protein transport and localization (ADAPTOR PROTEIN COMPLEX 4E, AP4E; ADAPTOR PROTEIN-1 MU-ADAPTIN 2, AP1M2) (Class 1 in Fig. 4C; Supplementary Data Set 20). In our comparative analysis, PCW-type CESAs, such as CESA1 CESA2, and CESA10, were identified as candidate targets for ubiquitination after VND7 induction (Class 2 in Fig. 4C; Supplementary Data Set 21). During xylem vessel cell differentiation, CESA proteins are shifted from PCW-type to SCW-type on the plasma membrane, leading to a decrease in PCW-type CESA abundance (Watanabe et al. 2018). In addition, the SCW-specific CesA7 can be phosphorylated at two serine residues, and a relationship with protein degradation via the 26S proteasome pathway has been proposed (Taylor 2007). In the current study, although the transcripts of PCW-type *CESA* genes did not change in response to DEX treatment, the ubiquitination levels of PCW-type CESA proteins increased (Supplementary Data Set 21). These results strongly suggest that PCW-type CESAs are active targets of protein ubiquitination and degradation during xylem vessel cell differentiation. We also identified the corresponding ubiquitination sites in CESA proteins (Supplementary Fig. S17). How the ubiquitination of these sites contributes to the regulation of CESA activity and/or turnover is beyond the scope of the present study; notably, similar results of PCW-type CESA ubiquitination were observed in ubiquitination analysis performed by Hannah et al. with the *VND7-VP16-GR* system, and they found significant decreases in peptide abundance of PCW-CESAs from 0 to 24 h of DEX induction. These results strongly support the involvement of ubiquitination in CESA turnover (Hannah et al.). Thus, we believe that the information on ubiquitinated proteins would contribute to our molecular understanding of SCW formation and facilitate improvements in woody biomass production.

### VND7 function is regulated by ubiquitination

The suppression of ectopic xylem vessel cell differentiation in *seiv* mutants also suggests the importance of protein ubiquitination for the activation of VND7 function itself. Indeed, we determined that VND7 can be a direct target of protein ubiquitination: the K94, K105, and K260 residues of VND7 are ubiquitinated in vivo (Fig. 6). The transactivation activity of VND7 was reduced by the K94R mutation, which blocks protein ubiquitination. These results emphasize the importance of the ubiquitination of K94 located in the conserved NAC subdomain III, which is important for DNA binding and protein–protein interactions (Fig. 6; Olsen et al. 2005; Yamaguchi et al. 2008). Thus, the ubiquitination of K94 likely plays a major role in regulating VND7 structure for DNA binding and/or dimerization, thereby regulating its transactivation activity.

VND7 is a highly unstable protein that is thought to be degraded via the 26S proteasome, as MG132 treatment significantly increased its abundance (Yamaguchi et al. 2008). We did not detect a change in VND7 protein levels in our proteome data, possibly because our sampling time (after 6 h of induction) was too early to observe its degradation. Interestingly, K260 is located close to C264, which is a target site of *S*-nitrosylation (Kawabe et al. 2018; Ohtani and Demura 2019). *S*-nitrosylation at the C264 residue of VND7 is thought to negatively regulate its activity (Kawabe et al. 2018; Ohtani and Demura 2019). Therefore, perhaps the ubiquitination at K260 affects the function of VND7 via its interaction with C264 *S*-nitrosylation. Moreover, different types of ubiquitin chains have diverse effects on protein behavior. Our Y2H assay revealed no direct interaction between VND7 and SEIV proteins identified here (Supplementary Fig. S16). Thus, the effects of ubiquitination on VND7 activity and the substrates of SEIV proteins need to be determined in future work.

Over the past few decades, much effort has been made to uncover the transcriptional regulatory network of xylem vessel formation and SCW biosynthesis. In this study, we successfully elucidated the crucial roles of protein ubiquitination in the regulation of xylem vessel functionality. Our data revealed (at least) two aspects of the importance of protein ubiquitination for xylem vessel cell differentiation: (1) ubiquitination of the K94 residue regulates the transactivation activity of VND7 to fully induce the expression of genes related to xylem vessel cells, and (2) the ubiquitination of proteins targeted for degradation shapes the proteome to facilitate xylem vessel cell differentiation. Post-translational modifications, including protein ubiquitination, function in plant responses to environmental stress (Hershko and Ciechanover 1998; Vierstra 2009; Marino et al. 2012; Zhou et al. 2017). Further analysis of the ubiquitination-mediated regulation of xylem vessel cell differentiation could help uncover the hidden molecular hubs that integrate environmental and developmental signals into xylem vessel functionality.

## Materials and methods

### Plant materials and growth conditions

The Arabidopsis (*Arabidopsis thaliana*) plants carrying *35S:VND7-VP16-GR* and *35S:VP16-GR* (vector control) were described in Yamaguchi et al. (2010a). The *suppressor of ectopic xylem vessel cell differentiation induced by VND7* (*seiv*) mutants were previously isolated from an EMS-mutagenized *VND7-VP16-GR* pool (Kawabe et al. 2018; Ohtani and Demura 2019; Pawittra et al. 2020). Four dominant *seiv* mutants (*seiv3*, *seiv4*, *seiv6*, and *seiv9*) were used in this study. For the generation of *SEIVpro:GUS* lines, we used the wild-type plants of Columbia (Col) strain, and the homozygous lines for *SEIVpro:GUS* constructs were used for the analysis. Seeds were surface-sterilized with 70% (v/v) ethanol with 0.1% Triton-X (w/v) and sown on Murashige and Skoog (MS) medium containing 1% (w/v) sucrose and 0.05% (w/v) 2-Morpholinoethanesulfonic acid (MES), solidified with 0.25% (w/v) gellan gum (pH 5.7). The plates were maintained at 4 °C in the dark for 2 to 3 d and incubated at 22 °C in a plant growth chamber under a 16-h light/8-h dark photoperiod. For seed harvesting, 2-week-old seedlings grown on plates were transferred to soil (1:1 mixture of red ball soil and vermiculite) and cultured at 22 °C under a 16-h light/8-h dark photoperiod, using the fluorescent lamp (LifelookN HG, FL20SEX-N-HG, NEC) with the intensity of 45∼85 *µ*mol m^−2^ s^−1^. In addition, we determined several light quality indicators, including Illuminance (Lux), PFD (Photon Flux Density), and PPFD (Photosynthetic Photon Flux Density), with LIGHT ANALYZER (LA 105; www.nihonika.co.jp). These indicators are shown in Supplementary Table S1.

### Plasmid construction

To verify the point mutations responsible for the suppressed DEX-induced ectopic xylem vessel cell differentiation in the *seiv* mutants, the genomic sequences of *PUB46*, *UBP1*, *FBX*, and *PUB36* containing the ∼2 kb upstream and ∼500 bp downstream regions were amplified from genomic DNA derived from *seiv3*, *seiv4*, *seiv6*, and *seiv9*, respectively. The PCR products were cloned into the pENTR/D-TOPO entry vector (Invitrogen) and transferred into the Gateway destination vector pBG (Kubo et al. 2005) using LR Clonase (Invitrogen).

To clarify the spatial expression patterns of the *SEIV* genes, chimeric reporter genes encoding β-glucuronidase (GUS) under the control of the *SEIV* promoters were utilized. The upstream regions (∼2 kb) of *PUB46*, *UBP1*, *FBX*, and *PUB36* were amplified using genomic DNA as templates. The PCR fragments were cloned into the pENTR/D-TOPO entry vector (Invitrogen) and integrated into the Gateway destination vector pBGGUS (Kubo et al. 2005) using LR Clonase (Invitrogen).

For site-directed mutagenesis, lysine to arginine (K-R) substitution mutations were introduced into a full-length VND7 sequence using mutagenic primers, and multiple K-R mutants were subsequently generated by PCR. The resulting fragments and X1E1 sequence (−148 to −96 region of the Arabidopsis *XCP1* promoter) (Yamaguchi et al. 2011) were cloned into pENTR/D-TOPO and integrated into pA35G and pAGL (Endo et al. 2015), respectively, using LR Clonase (Invitrogen).

For the yeast 2-hybrid assay, the CDS of *VND7*, *PUB46*, *UBP1*, *FBX*, and *PUB36* were amplified from Col-0 cDNA. The PCR products were cloned into the pENTR/D-TOPO entry vector (Invitrogen) and transferred into the Gateway destination vector pAD-GAL4-GWRFC and pBD-GAL4-GWRFC (Yamaguchi et al. 2008) using LR Clonase (Invitrogen). The primer information is provided in Supplementary Data Set 27.

### Plant transformation

The resulting plasmids were introduced into *Agrobacterium tumefaciens* strain GV3101 (pMP90) by electroporation. Four-week-old Arabidopsis *VND7-VP16-GR* plants and ecotype Col-0 were transformed with the pBG and pBGGUS constructs using the floral dip method (Clough and Bent 1998). The resulting seeds were screened based on antibiotic resistance to select transgenic plants, and the positive lines were used to generate T_3_ homozygous lines. We obtained at least 10 independent transgenic lines for each construct, and checked the *GUS* expression patterns. Finally we picked up the T_3_ transgenic lines with typical expression patterns for each construct, and showed their expression patterns in Fig. 1 and Supplementary Fig. S2.

### Dexamethasone (DEX) treatment of seedlings

For DEX treatment, 7-day-old seedlings grown on MS medium were immersed in 10 mL of sterile water containing 10 *µ*M dexamethasone (DEX; SIGMA) or dimethyl sulfoxide (DMSO) (mock control). The plates were then placed in a plant growth chamber, and seedlings were sampled at specific time points as indicated.

### Microscopy

To observe endogenous xylem vessel formation, 7-day-old wild-type *VND7-VP16-GR* and *seiv* seedlings were fixed in 90% (v/v) acetone for 1 week at −30 °C. To observe ectopic xylem vessel formation, 7-day-old wild-type *VND7-VP16-GR* and *seiv* seedlings treated with 10 *µ*M DEX for 3 d were fixed in 90% (v/v) acetone for 1 week at −30 °C. The samples were sequentially immersed in 90, 70, 50, and 30% (v/v) acetone for 5 min per step and hydrated by immersion in sterile water. The samples were mounted in clearing solution [chloral hydrate: water: glycerol 8:1:2 (w/v/v)]. Images were taken under a light microscope (BX53; Olympus).

### Histochemical assay of GUS activity

The *promoter:GUS* reporter lines were grown for the indicated period of time and fixed in 90% (v/v) acetone at −30 °C for 2 h. After rinsing with 50 mm phosphate buffer (pH 7.4), the samples were incubated overnight in X-Gluc solution (1 mm 5-bromo-4-chloro-3-indolyl-beta-D-glucuronic acid, cyclohexylammonium salt; 1 mm potassium ferrocyanide; 1 mm potassium ferricyanide; 50 mm phosphate buffer, pH 7.4) at 37 °C. Following incubation, the samples were rinsed in 50 mm phosphate buffer (pH 7.4) and fixed in FAA [5% (w/v) formaldehyde, 5% (v/v) acetic acid, 20% (v/v) EtOH] at 4 °C overnight. Before observation, the samples were dehydrated in 50% (v/v) EtOH for 2 min, followed by 100% (v/v) EtOH for 10 min. To generate sections, the dehydrated GUS-stained samples were embedded in Technovit 7100 resin (Heraeus Kulzer, Wehrheim, Germany), cut into 8-µm-thick sections, and observed.

### Reverse transcription-quantitative PCR (RT-qPCR) analysis

Total RNA was extracted from the samples using an RNeasy Plus Mini Kit (QIAGEN) according to the manufacturer's instructions. The concentration and quality of the RNA were determined using a NanoDrop spectrophotometer (Thermo Fisher Scientific). First-strand cDNA was synthesized from 1 *µ*g DNase-treated RNA by reverse transcription using oligo(dT)18 primers (Invitrogen) and SuperScript IV (Invitrogen). Quantitative PCR was carried out using a Light Cycler 480 system (Roche) with TB Green Premix EX Taq II (TaKaRa). The *ACTIN2* gene was used as the internal control to normalize gene expression levels. The relative expression levels were calculated by the 2^−ΔΔ^ C_T_ method. The primer sequences are listed in Supplementary Data Set 27.

### Xylem transport assay

The xylem transport assay was performed as described in Endo et al. (2019) with slight modifications. Fourteen-day-old Col-0, wild-type *VND7-VP16-GR*, and *seiv* seedlings grown on 1/2 MS medium solidified with 0.8% (w/v) gellan gum were used for the xylem transport assay. Before the assay, the lid of the plate was kept slightly open for 20 h to control the relative humidity. The tips of primary roots were removed, and 100 *µ*L of 1 mm rhodamine solution was applied to the excised root tip. Fluorescence was observed every minute in the dark under an MSV269 (Leica) fluorescence microscope with maximum intensity and“ET DSR” filter. Fluorescent signals were photographed with an iPhone 7 (A1779) using the i-NTER SHOT app with the settings of temperature = 3000 K, tint = 0, and Brightness = 704. The signals were quantified using Image J software (https://imagej.net/software/fiji/). We performed the least significant difference (LSD) test to check the statistical significant difference among lines (Supplementary Data Set 1).

### RNA-seq analysis

Seven-day-old wild-type *VND7-VP16-GR*, *seiv*, and vector control seedlings were treated with DEX or DMSO (mock) for 6 h. Samples were collected, immediately frozen in liquid nitrogen, and stored until RNA extraction. Total RNA was extracted from the samples using an RNeasy Plant Mini Kit (Qiagen) according to the manufacturer's instructions. These procedures were independently repeated for 3 times. The quality and quantity of purified RNA were examined using an Agilent 2100 Bioanalyzer (Agilent). Two µg of total RNA was used for RNA library construction, which was subsequently subjected to high-throughput sequencing using the BGISEQ-500 system (BGI Japan). For RNA-seq data analysis, short reads were mapped against the Arabidopsis genome (TAIR10) using HISAT2 (v2.1.0; https://ccb.jhu.edu/software/hisat2). Gene expression was calculated using StringTie (v1.3.4d; https://ccb.jhu.edu/software/stringtie). Relative expression level was calculated as TPM (transcripts per million). Differentially expressed genes (DEGs) were detected using the R package TCC (v1.22.1), which internally utilized the R package edgeR (v3.24.2). Genes with *q*-value of <0.05 and |log2FC| >1 were identified as DEGs.

### Library preparation for whole-genome resequencing

The *seiv* mutants were backcrossed twice with wild-type *VND7-VP16-GR*, and then selfed to generate homozygous *seiv* seedlings. For each *seiv* mutant, genomic DNA libraries were prepared from 40 independent homozygous lines, and a total of 400 10-d-old seedlings were collected and subjected to nuclei isolation using a CelLytic PN Isolation/Extraction Kit following the semi-pure preparation protocol (Sigma-Aldrich). The genomic DNA was sheared using a Covaris S2 ultrasonicator (Covaris) to produce 100-bp fragments. Libraries for sequencing were generated from fragmented genomic DNA using a NEBNext DNA Library Prep Reagent Set for Illumina (New England Biolabs), including DNA-end repair, dA-tailing of blunt-ended DNA, and adaptor ligation with an additional adaptor oligo kit (for *seiv3*, *seiv4*, and *seiv9*, NEBNext Singleplex Oligos for Illumina; for *seiv6*, NEBNext Multiplex Oligos for Illumina; both from New England Biolabs). The ligated products were amplified using KAPA Library Amplification Kits (Kapa Biosystems) with primers (NEBNext Universal PCR Primer for Illumina and NEBNext Index 1 primer). Fragments of 200 to 450 bp were purified after gel electrophoresis in a 2% (w/v) agarose gel containing SYBR Safe DNA Gel Stain (Thermo Fisher Scientific). An Illumina Genome Analyzer IIx (GAIIx; Illumina) was used for *seiv3*, *seiv4*, and *seiv9*, whereas Illumina HiSeq 4000 (Illumina) for *seiv6*.

### Single-nucleotide polymorphism (SNP) detection

The raw output data were processed using the bcl2fastq Conversion program (Illumina) to obtain sequencing data with quality values (fastq files), which were subsequently mapped to the Arabidopsis Col-0 reference genome (TAIR10 release) using bowtie2 software (v2.2.9; Langmead and Salzberg 2012 ) with default parameters. Collective calculation for single-nucleotide polymorphism (SNP) calling was performed with the GATK (Genome Analysis Toolkit; Mckenna et al. 2010) HaplotypeCaller subprogram (v3.6) with default settings. The “effect” or “impact” information of each SNP was then appended using the SnpEff program (v4.2; Cingolani et al. 2012) based on the genome annotation (Ensembl Plant Release 31, March 2016). To identify unique SNPs for each *seiv* mutant, AC (allele count in genotypes for each ALT allele) information was used for filtering. Finally, SNPs found in the reference genotype *VND7-VP16-GR* were subtracted from the list of SNPs to generate final lists of SNPs unique to each *seiv* mutant.

### Protein extraction

Ten-day-old vector control, wild-type *VND7-VP16-GR*, *seiv6*, and *seiv9* seedlings were treated with sterile water containing 10 *μ*M DEX and DMSO (mock control) for 6 h. The samples were quickly frozen in liquid nitrogen, ground to a fine powder, and resuspended in urea lysis buffer (8 m urea in 100 mm Tris-HCl, pH 8.5; 5 mm DTT). The mixture was incubated at room temperature for 30 min with vortexing. A clear supernatant was obtained after three rounds of centrifugation. Protein levels were determined by Pierce 660 nm Protein Assay (Thermo Fischer Scientific) using bovine serum albumin as a control.

### Sample preparation and tryptic digestion

The protein solution was alkylated with chloroacetamide (CAA) (550 mm stock, 14 mm final concentration) for 30 min at room temperature in the dark, after which an aliquot corresponding to 20 mg total protein was subjected to filter-assisted digestion. In brief, proteins were loaded onto spin filters (Millipore, 30 kD cutoff) by centrifugation for 40 min (4k × *g*) at RT to retain 1.5 mL on the filter. Samples were washed with 10 mL urea buffer (8; in 100 mm Tris, pH 8.5) and concentrated to 1 mL by centrifugation. After dilution (8 mL 100 mm Tris, pH 8.5) samples were digested with 200 *µ*g trypsine (1:100) o/n at 37 °C. Peptides were collected by centrifugation (30 min, 4k × *g*), filters were washed with 1 mL Tris and combined filtrates were acidified with TFA (500 *µ*L). Samples were desalted using Sep-Pack cartridges (360 mg, Waters): columns were conditioned using MeOH (5 mL), buffer B [80% (v/v) acetonitrile, 0.1% (v/v) TFA] (5 mL) and buffer A [0.1% (v/v) TFA] (1 × 5 mL, 1 × 10 mL). Samples were loaded by gravity flow, washed with buffer A (3 × 5 mL) and eluted with buffer B (2 × 2 mL, 1 × 1 mL). 50 *µ*L of the combined eluates were dried for total proteome analysis. The dried pellet was dissolved in 20 *µ*L A* buffer [2% (v/v) ACN, 0.5% (v/v) FA] to determine peptide concentration by Nanodrop measurement. Next, an aliquot was diluted 1:100 with A* and submitted to MS analysis using BoxCar methodology (Meier et al. 2018). For library measurement, 23 *µ*L of diluted peptides from each condition (grouped by replicate) were combined and 169 *µ*L of this mixture was submitted to Strong CationExchange (SCX) fractionation.

In brief, StageTips were prepared using 6 layers of SPE disk (Empore Cation 2,251 material) activated with acetonitrile and 1% (v/v) TFA (100 *µ*L each) and washed with buffer A [water, 0.2% (v/v) TFA] (100 *µ*L) by spinning 5 min (1.5k × *g*), samples were acidified to 1% (v/v) TFA, loaded by centrifugation (10 min, 800*×g*) and washed with buffer A (5 min, 1.5k × *g*) (100 *µ*L). Fractionation was carried out using an ammonium acetate gradient [20% (v/v) ACN, 0.5% (v/v) FA] starting from 50 mm to 300 mm for 5 fractions and a final elution step using 5% (v/v) ammonium acetate, 80% (v/v) ACN. All fractions were eluted by centrifugation (5 min, 500*×g*) using 2 × 30 *µ*L eluent. The fractions were dried and taken up in 10 *µ*L A* buffer. Peptide concentration was determined by Nanodrop and samples were diluted to 0.2 *µ*g/µL for measurement.

### Immunoaffinity enrichment of ubiquitinated peptides

The combined eluates of tryptic peptides from the aforementioned SepPak purification were evaporated and submitted to ubiquitin-IP. Ubiquitinated peptides were enriched by using the PTMScan Ubiquitin Remnant Motif (K-ε-GG) Kit (Cell Signaling). Briefly, lyophilized peptides were resuspended in PTMScan IAP buffer (50 mm MOPS/NaOH, pH 7.2, 10 mm Na_2_HPO4, and 50 mm NaCl) and incubated with K-ε-GG antibody beads at 4 °C for 2 h. Then, the beads were washed twice with IAP buffer and repeated the washing step three times with chilled HPLC-grade water. The Kub (lysine (K) ubiquitinated) modified peptides were eluted from the beads with 0.15% (v/v) TFA. The resulting eluates were desalted using StageTips with C18 Empore disk membranes (3 m) (Rappsilber et al. 2003), final elution was done with buffer B* (40% acetonitrile, 0.5% FA) (2 × 25 *µ*L). After drying samples were dissolved in 10 *µ*L A* (2% ACN, 0.1% TFA), for measurement samples were diluted with 5 *µ*L A*.

### LC-MS/MS data acquisition

To detect the protein and ubiquitination dynamics during xylem vessel formation, proteins extracted from the seedlings mock- or DEX-treated for 6 h, ubiquitinated tryptic peptides with remnant diGly tag were subjected to LC-MS/MS analysis. Ubiquitin-IP and library samples were analyzed using an EASY-nLC 1200 (Thermo Fisher) coupled to a Q Exactive Plus mass spectrometer (Thermo Fisher). Peptides were separated on 16 cm frit-less silica emitters (New Objective, 75 *µ*m inner diameter), packed in-house with reversed-phase ReproSil-Pur C18 AQ 1.9 *µ*m resin (Dr. Maisch). Peptides were loaded on the column and eluted for 115 min using a segmented linear gradient of 5% to 95% solvent B (0 min: 5%; 0 to 5 min: 5%; 5 to 65 min: 20%; 65 to 90 min: 35%; 90 to 100 min: 55%; 100 to 105 min: 95%, 105 to 115 min: 95%) (solvent A 0% ACN, 0.1% FA; solvent B 80% ACN, 0.1%FA) at a flow rate of 300 nL/min. Mass spectra were acquired in data-dependent acquisition mode with a TOP15 method. MS spectra were acquired in the Orbitrap analyzer with a mass range of 300 to 1750 m/z at a resolution of 70,000 FWHM and a target value of 3 × 10^6^ ions. Precursors were selected with an isolation window of 1.3 m/z. HCD fragmentation was performed at a normalized collision energy of 25. MS/MS spectra were acquired with a target value of 10^5^ ions at a resolution of 17,500 FWHM, a maximum injection time (max.) of 55 ms and a fixed first mass of m/z 100. Peptides with a charge of +1, greater than 6, or with unassigned charge state were excluded from fragmentation for MS^2^, dynamic exclusion for 30 s prevented repeated selection of precursors.

BoxCar samples were analyzed using an EASY-nLC 1200 (Thermo Fisher) coupled to a Q Exactive Plus mass spectrometer (Thermo Fisher) using the same gradient settings as described above for ubiquitin-IP and library samples. Mass spectra were acquired in a data-independent manner using the MaxQuant.Live application (Wichmann et al. 2019). The acquisition was initiated using the “magic scan” protocol and consisted of one full MS scan with a a mass range of 300 to 1,650 m/z at a resolution of 140,000 FWHM, a target value of 3 × 10^6^ ions and a maximum injection time of 20 ms. This was followed by two BoxCar scans, each consisting of 10 boxes with 1 Da overlap and a scan range from 400 to 1,200 m/z. The maximum injection time for a BxCar scan was set to 250 ms, with a resolution of 140,000 FWHM, a target value of 5 × 10^5^ ions. The 5 most abundant ions from each BoxCar scan were selected for HCD fragmentation at a normalized collision energy of 27. Precursors were selected with an isolation window of 1.4 m/z. MS/MS spectra were acquired with a target value of 10^5^ ions at a resolution of 17,500 FWHM, a maximum injection time (max.) of 28 ms and a fixed first mass of m/z 50. Peptides with a charge of +1, greater than 5 or with unassigned charge state were excluded from fragmentation for MS^2^, dynamic exclusion for 30 s prevented repeated selection of precursors.

### Data analysis for BoxCar analysis of total proteome data

Raw data were processed using MaxQuant software (version 1.6.3.4, http://www.maxquant.org/) (Cox and Mann 2008) with label-free quantification (LFQ) and iBAQ enabled (Tyanova et al. 2016). Library samples and BoxCar samples were grouped into separate parameter groups. In the group specific parameters, library samples were set to “Standard” type and BoxCar samples to “BoxCar” type, in the Misc. setting the Match type for library samples was set to “match from” and for BoxCar to “match from and to”.

MS/MS spectra were searched by the Andromeda search engine against a combined database containing the sequences from Arabidopsis (TAIR10_pep_20101214; https://www.arabidopsis.org/download/list?dir=Proteins%2FTAIR10_protein_lists) and sequences of 248 common contaminant proteins and decoy sequences. Trypsin specificity was required and a maximum of two missed cleavages allowed. Minimal peptide length was set to seven amino acids. Carbamidomethylation of cysteine residues was set as fixed, oxidation of methionine and protein N-terminal acetylation were set as variable modifications. The match between runs option was enabled. Peptide-spectrum-matches and proteins were retained if they were below a false discovery rate of 1% in both cases.

Statistical analysis of the MaxLFQ values was carried out using Perseus (version 1.5.8.5, http://www.maxquant.org/). Quantified proteins were filtered for reverse hits and hits “only identified by site” and MaxLFQ values were log2 transformed. After grouping samples by condition (control mock, control DEX, VND7 mock, VND7 DEX, *seiv6* mock, *seiv6* DEX, *seiv9* mock, *seiv9* DEX), only those proteins were retained for the subsequent analysis that had four valid values in one of the conditions. Missing values were imputed from a normal distribution, using the default settings in Perseus (1.8 downshift, separately for each column). Volcano plots were generated in Perseus using an FDR of 5% and an *S0* = 1.

### Data analysis for ubiquitome data

Raw data were processed using MaxQuant software (version 1.5.7.4, http://www.maxquant.org/) (Cox and Mann 2008) with label-free quantification (LFQ) and iBAQ enabled (Tyanova et al. 2016). MS/MS spectra were searched by the Andromeda search engine against a combined database containing the sequences from Arabidopsis (TAIR10_pep_20101214; https://www.arabidopsis.org/download/list?dir=Proteins%2FTAIR10_protein_lists) and sequences of 248 common contaminant proteins and decoy sequences. Trypsin specificity was required and a maximum of two missed cleavages allowed. Minimal peptide length was set to seven amino acids. Carbamidomethylation of cysteine residues was set as fixed, GlyGly modification of the lysine sidechain, oxidation of methionine and protein N-terminal acetylation were set as variable modifications. The match between runs option was enabled. Peptide-spectrum-matches and proteins were retained if they were below a false discovery rate of 1% in both cases.

Statistical analysis of the intensity values obtained for the GlyGly modified sites (“GlyGly(K)Sites.txt” output file) was carried out using Perseus (version 1.5.8.5, http://www.maxquant.org/). Quantified sites were filtered for reverse hits, the site table was expanded and intensity values were log2 transformed. After grouping samples by condition (control mock, control DEX, VND7 mock, VND7 DEX, *seiv6* mock, *seiv6* DEX, *seiv9* mock, *seiv9* DEX), only those sites were retained for the subsequent analysis that had three valid values in one of the conditions. Next, GlyGly sites were filtered for a localization probability >0.75 and missing values were imputed from a normal distribution with a 2.0 downshift, separately for each column. Volcano plots were generated in Perseus using an FDR of 5% and *S0* = 1.

### Gene Ontology (GO) enrichment analysis

Gene Ontology (GO) enrichment analysis of DEGs was performed using the clusterProfiler package (v4.4.4) in R. *P*-value was adjusted by the Benjamini and Hochberg multi-test method, and GO terms with FDR < 0.05 were selected as overrepresented terms.

### Transient expression assay in Arabidopsis protoplasts

Arabidopsis protoplasts were isolated from Arabidopsis T87 culture cells according to Kawabe et al. (2018), with slight modifications. T87 culture cells in exponential phase (3-d-old suspension cultures) were resuspended in an enzyme solution (pH 5.6)containing 1% (w/v) Cellulase Onozuka RS (Yakult Pharmaceutical Ind. Co. Ltd.), 0.05% (w/v) Pectolyase Y-23 (Kyowa Chemical Ind. Co., Ltd.), 5 mm EGTA, and 0.4 m mannitol, and incubated at room temperature with gentle agitation (25 rpm) for 90 min. The protoplasts were filtered sequentially through 70-µm and 40-µm nylon meshes, and the pellets were washed in a solution containing 1 vol. 500 mm mannitol and 2 vol. 200 mm CaCl_2_. The pellets containing isolated protoplasts were washed in a solution containing 2 vol. of 0.5 m mannitol and 1 vol. of 200 mm CaCl_2_, resuspended in W5 buffer (154 mm NaCl; 125 mm CaCl_2_; 5 mm KCl; 1.5 mm MES, pH 5.7; 5 mm glucose), and incubated on ice for 30 min. Protoplast transfection was performed by the polyethylene glycol (PEG)-mediated method as described by Kawabe et al. (2018) using approximately 4 × 10^5^ cells in MMg solution (0.4 m mannitol; 15 mm MgCl_2_; 5 mm MES, pH 5.7) and 1 *µ*g of the effector and reporter constructs. The activity of the reporter gene was normalized using 0.4 *μ*g of Renilla luciferase expression plasmid (Ohta et al. 2000). Transfected protoplasts were incubated in W5 buffer in the dark at 22 °C for 16 h. Luciferase activity was measured using the Dual Luciferase Reporter Assay System (Promega) following the manufacturer's instructions. We repeated the experiments 3 times independently, and performed Student's t-test. (Supplementary Data Set 26).

### Yeast two hybrid analysis

The plasmid constructed on pAD-GAL4-GWRFC or pBD-GAL4-GWRFC (Yamaguchi et al. 2008) was introduced into *S. cerevisiae* strain AH109 (Clontech, http://www.clontech.com/) by S.c. EasyComp^TM^ Transformation Kit (Invitrogen). The transformants were incubated at 30 °C on minimal Sd medium (Clontech) either lacking tryptophan and leucine, or tryptophan, leucine and histidine. pAD-WT and pBD-WT (Stratagene) were used as the positive controls, pAD-MCS and pBD-MCS (Yamaguchi et al. 2008) were used as the negative controls. We repeated the experiments 3 times independently.

### Accession numbers

Sequence data from this article can be found in The Arabidopsis Information Resource (https://www.arabidopsis.org/index.jsp) under the following accession numbers: VND7 (AT1G71930), PUB36 (AT3G61390), PUB46 (AT5G18320), FBX (AT5G39250), UBP1 (AT2G32780).

## Supplementary Material

koae221_Supplementary_Data

## Data Availability

Raw RNA-seq data are available from the DDBJ database (https://www.ddbj.nig.ac.jp/index-e.html) under accession number PSUB021919. The mass spectrometry proteomics data have been deposited to the ProteomeXchange Consortium via the PRIDE (Perez-Riverol et al. 2022) partner repository with the dataset identifier PXD048830.

## Supplementary data

The following materials are available in the online version of this article.

**Supplementary Figure S1.** Ubiquitination-associated genes are responsible for the suppressed DEX-induced ectopic xylem vessel cell differentiation of the *seiv* mutants. (Supports Fig. 1)

**Supplementary Figure S2.** Expression patterns of the *SEIV* genes. (Supports Fig. 1)

**Supplementary Figure S3.** Effects of the *seiv* mutations on ectopic xylem vessel formation in primary roots. (Supports Fig. 2)

**Supplementary Figure S4.** Effect of the *seiv* mutations on endogenous xylem vessel formation in cotyledons. (Supports Fig. 2)

**Supplementary Figure S5.** Effect of the *seiv* mutations on endogenous xylem vessel formation in hypocotyls. (Supports Fig. 2)

**Supplementary Figure S6.** Effect of the *seiv* mutations on endogenous xylem vessel formation in leaf veins. (Supports Fig. 2)

**Supplementary Figure S7.** Xylem transport ability is impaired in the *seiv* mutants. (Supports Fig. 2)

**Supplementary Figure S8.** Gene ontology (GO) term analysis for downregulated genes in *seiv* mutants compared with wild-type *VND7-VP16-GR* under DEX treatment. (Supports Fig. 3)

**Supplementary Figure S9.** Gene ontology (GO) term analysis for upregulated genes in *seiv* mutants compared with wild-type *VND7-VP16-GR* under DEX treatment. (Supports Fig. 3)

**Supplementary Figure S10.** Gene ontology (GO) term analysis for downregulated genes in *seiv* mutants compared with wild-type *VND7-VP16-GR* under mock treatment. (Supports Figs. 2 and 3)

**Supplementary Figure S11.** Expression levels of VND7-downstream genes in the vector control, wild-type *VND7-VP16-GR*, and *seiv* seedlings. (Supports Figs. 2 and 3)

**Supplementary Figure S12.** PCA of RNA-seq data of the wild-type *VND7-VP16-GR*, vector control, and *seiv* mutants. (Supports Fig. 3)

**Supplementary Figure S13.** Multiple Venn diagrams of upregulated and downregulated ubiquitination events in response to VND7 induction in wild-type *VND7-VP16-GR* and *seiv6*. (Supports Fig. 5)

**Supplementary Figure S14.** Ubiquitination level on VND7 during xylem vessel formation. (Supports Fig. 6)

**Supplementary Figure S15.** Reduced transcriptional activity of VND7 when K94 was substituted with Arg. (Supports Fig. 6)

**Supplementary Figure S16.** VND7 does not directly interact with SEIV proteins. (Supports Fig. 6)

**Supplementary Figure S17.** Ubiquitination events detected in CESA1, CESA3, CESA6-like (CESA2, CESA5), and CESA10 upon VND7 induction. (Supports Fig. 4)

**Supplementary Table S1.** Indicators of light quality.

**Supplementary Data Set 1.** Average fluorescence intensity (arbitrary units) in Col-0, wide-type *VND7-VP16-GR*, and *seiv* tissues (LSD test, *P* < 0.01).

**Supplementary Data Set 2.** Over-represented complete GO terms (Biological process) for genes contributing to Dimension 1 (Top 3,000 genes).

**Supplementary Data Set 3.** Over-represented complete GO terms (Biological process) for significantly upregulated genes after VND7 induction (6-h DEX treatment) of wild-type *VND7-VP16-GR.*

**Supplementary Data Set 4.** Over-represented complete GO terms (Biological process) for significantly downregulated genes after VND7 induction (6-h DEX treatment) of wild-type *VND7-VP16-GR.*

**Supplementary Data Set 5.** Over-represented complete GO terms (Biological process) for significantly upregulated genes in *seiv3* to the wild-type *VND7-VP16-GR* with DEX treatment.

**Supplementary Data Set 6.** Over-represented complete GO terms (Biological process) for significantly downregulated genes in *seiv3* to the wild-type *VND7-VP16-GR* with DEX treatment.

**Supplementary Data Set 7.** Over-represented complete GO terms (Biological process) for significantly upregulated genes in *seiv4* to the wild-type *VND7-VP16-GR* with DEX treatment.

**Supplementary Data Set 8.** Over-represented complete GO terms (Biological process) for significantly downregulated genes in *seiv4* to the wild-type *VND7-VP16-GR* with DEX treatment.

**Supplementary Data Set 9.** Over-represented complete GO terms (Biological process) for significantly upregulated genes in *seiv6* to the wild-type *VND7-VP16-GR* with DEX treatment.

**Supplementary Data Set 10.** Over-represented complete GO terms (Biological process) for significantly downregulated genes in *seiv6* to the wild-type *VND7-VP16-GR* with DEX treatment.

**Supplementary Data Set 11.** Over-represented complete GO terms (Biological process) for significantly upregulated genes in seiv9 to the wild-type *VND7-VP16-GR* with DEX treatment.

**Supplementary Data Set 12.** Over-represented complete GO terms (Biological process) for significantly downregulated genes in *seiv9* to the wild-type *VND7-VP16-GR* with DEX treatment.

**Supplementary Data Set 13.** Over-represented complete GO terms (Biological process) for genes contributing to Dimension 2 (Top 3,000 genes).

**Supplementary Data Set 14.** Over-represented complete GO terms (Biological process) for genes contributing to Dimension 3 (Top 3,000 genes).

**Supplementary Data Set 15.** Over-represented complete GO terms (Biological process) for genes contributing to Dimension 4 (Top 3,000 genes).

**Supplementary Data Set 16.** Over-represented complete GO terms (Biological process) for genes contributing to Dimension 5 (Top 3,000 genes).

**Supplementary Data Set 17.** List of significantly upregulated proteins after DEX treatment in wild-type *VND7-VP16-GR.*

**Supplementary Data Set 18.** List of significantly downregulated proteins after DEX treatment in wild-type *VND7-VP16-GR.*

**Supplementary Data Set 19.** Over-represented complete GO terms (Biological process) for significantly upregulated proteins after VND7 induction (6-h DEX treatment) of wild-type *VND7-VP16-GR.*

**Supplementary Data Set 20.** List of proteins increased in both transcriptome and ubiquitinome upon VND7 induction (Class 1).

**Supplementary Data Set 21.** List of proteins increased in only ubiquitinome upon VND7 induction (Class 2).

**Supplementary Data Set 22.** List of proteins decreased in both transcriptome and ubiquitinome upon VND7 induction (Class 3).

**Supplementary Data Set 23.** List of proteins decreased in both transcriptome and ubiquitinome upon VND7 induction (Class 4).

**Supplementary Data Set 24.** Over-represented complete GO terms (Biological process) for significantly downregulated ubiquitination events in *VND7-VP16-GR* upon DEX treatment.

**Supplementary Data Set 25.** Over-represented complete GO terms (Biological process) for significantly downregulated ubiquitination events in *seiv6* upon DEX treatment.

**Supplementary Data Set 26.** T-test results of LUC activity detected in transient report assay.

**Supplementary Data Set 27.** List of primers used in the study.
