# Supplementary material for: Active protein ubiquitination regulates xylem vessel functionality
Source: Plant Cell. 2024 Aug 2;36(9):3298–317. doi: 10.1093/plcell/koae221 (PMC11371170; doi:10.1093/plcell/koae221)
Supplement: koae221_Supplementary_Data [file koae221_supplementary_data.zip › Supplementary figures_revised MA.pdf]

Supplementary Data. Phookaew et al. (2024). Active protein ubiquitination regulates xylem vessel functionality. Plant Cell.

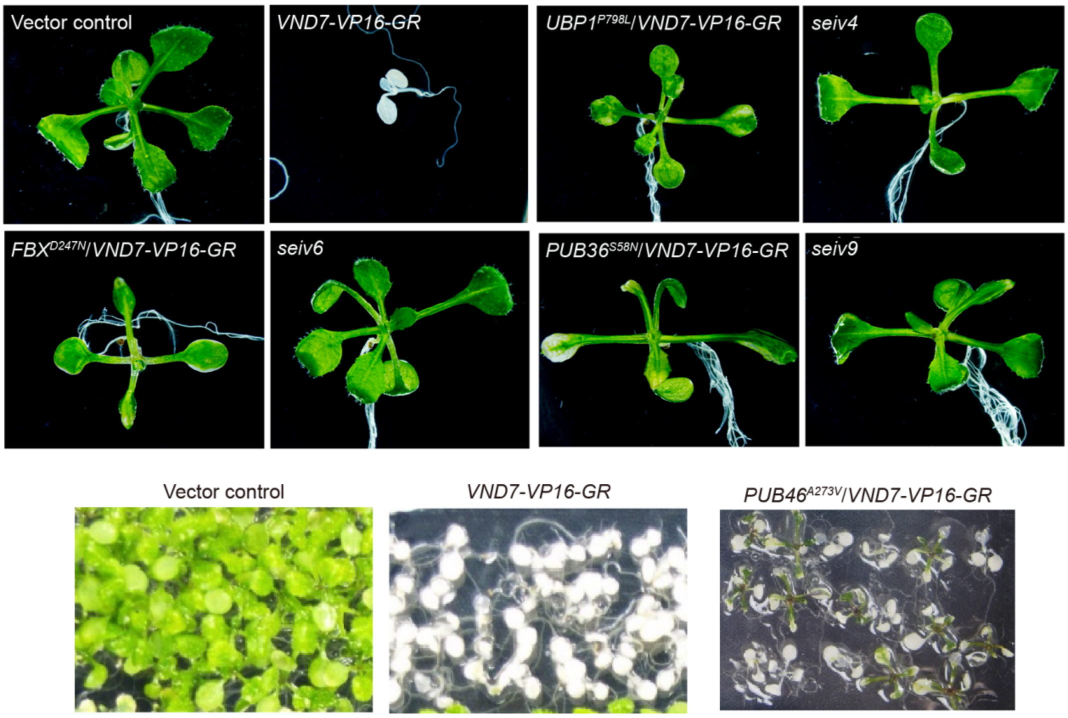

Supplementary Figure S1. Ubiquitination-associated genes are responsible for the suppressed DEX-induced ectopic xylem vessel cell differentiation of the *seiv* mutants. (Supports Figure 1) Arabidopsis wild-type *VND7-VP16-GR* plants transformed with the genomic fragment of the PUB46^{A273V}, UBP1^{P798L}, FBX^{D247N}, and PUB36^{S58N} mimicked the *seiv3*, *seiv4*, *seiv6*, and *seiv9* mutant phenotype. Seedling were grown for 7 days and subsequently treated with DEX for 4 days.

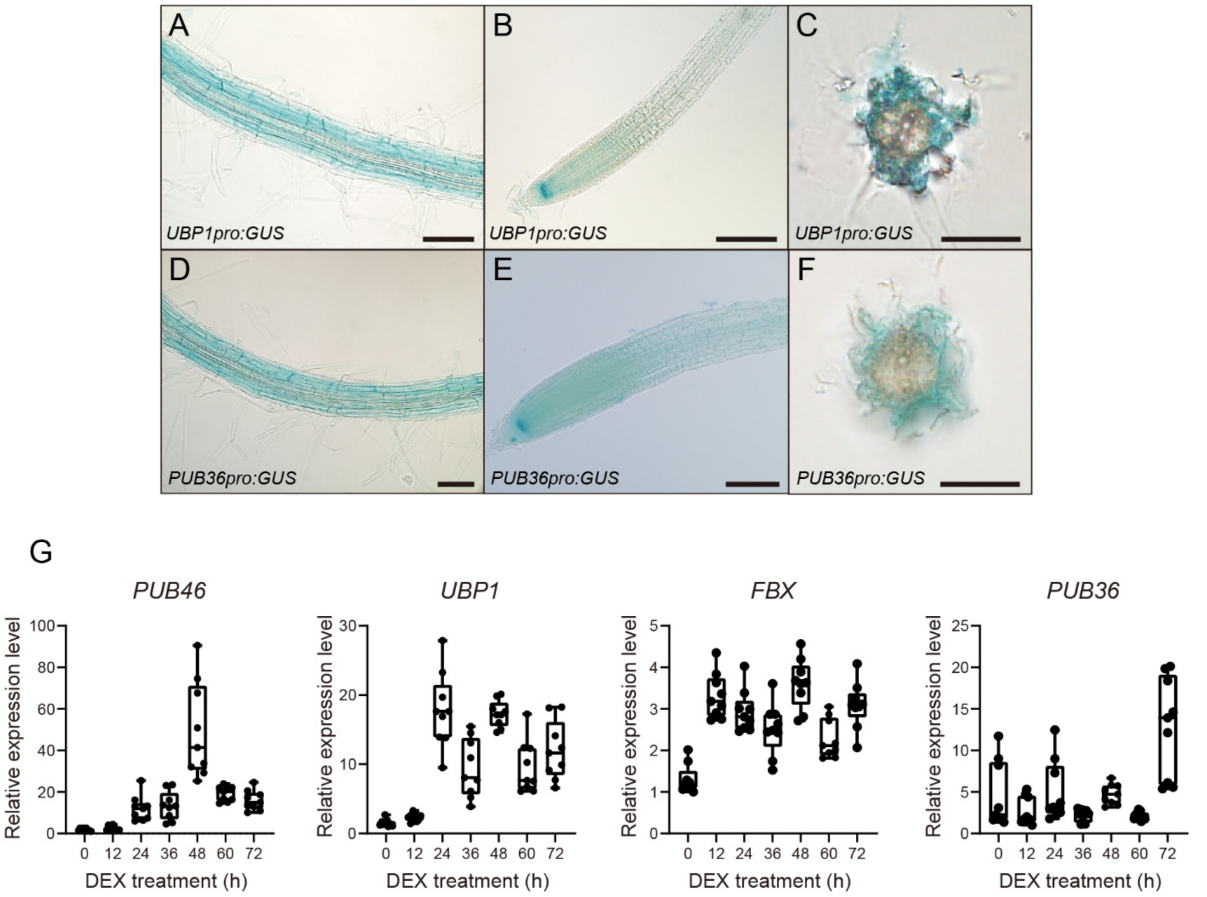

Supplementary Figure S2. Expression patterns of the *SEIV genes*. (Supports Figure 1) (A-F): Expression patterns of the *UBP1* and *PUB36* promoters. Seven-day-old Arabidopsis transgenic seedlings expressing *GUS* reporter gene driven by *UBP1* or *PUB36* promoter were subjected to GUS signal detection. Root stele (A, D), root tip (B, E), and transverse section of root regions (C, F). Bars = 100 µm. At least 3 independent lines were assayed for each construct. (G) Expression analysis of *SEIV* genes upon dexamethasone (DEX) treatment. *VND7-VP16-GR* seedlings were treated with 10 µM DEX and sampled every 12 h for RT-qPCR analysis. The expression levels of *PUB46*, *UBP1*, *FBX,* and *PUB36* were normalized to the internal control gene *ACTIN2* (n=3). Data are presented in the box and whiskers with all individual data points shown. The box represents the interquartile range (IQR), spanning from the 25th percentile (Q1) to the 75th percentile (Q3) of the data, with the median (50 percentile) marked by a line inside the box. Whiskers extend to 1.5 times the IQR from the quartiles.

Supplementary Data. Phookaew et al. (2024). Active protein ubiquitination regulates xylem vessel functionality. Plant Cell.

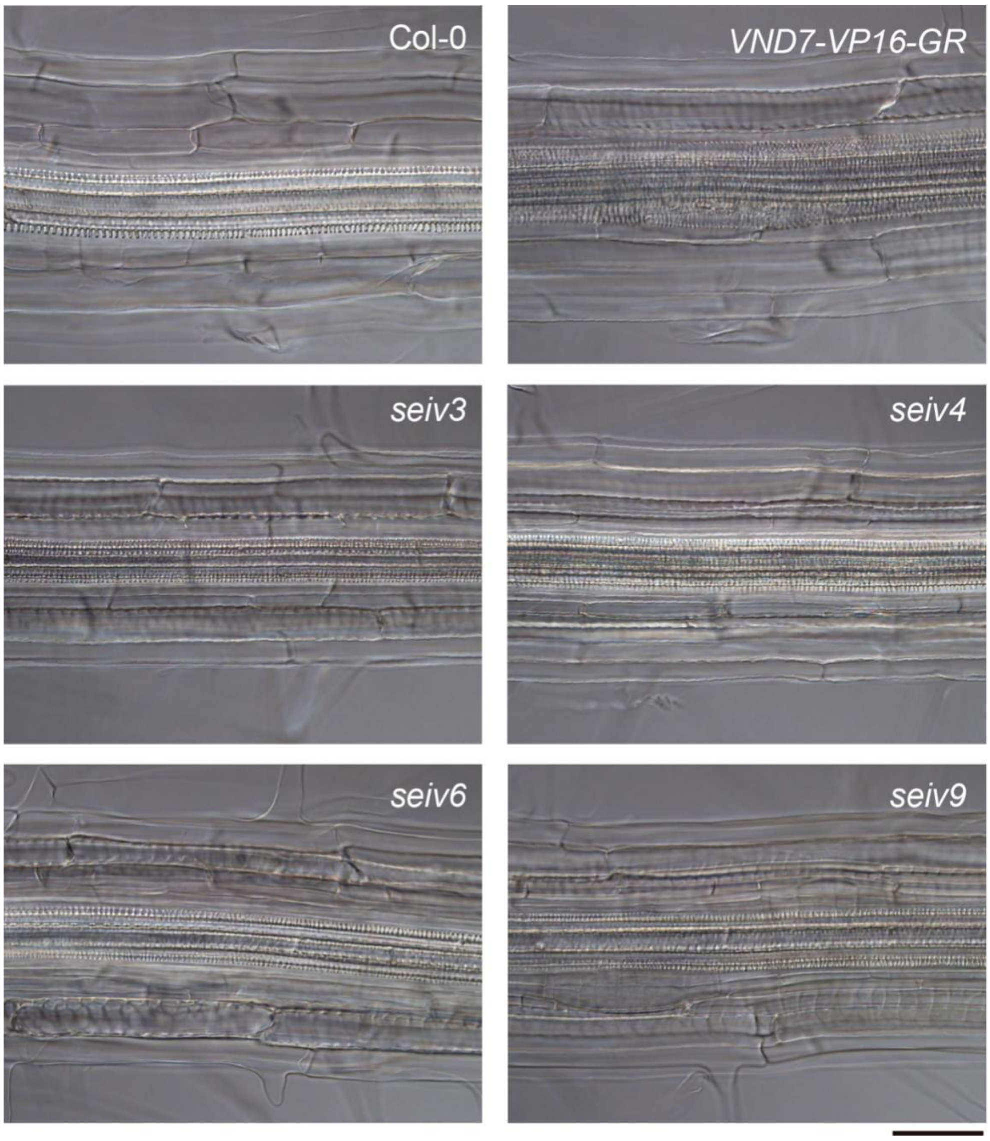

Supplementary Figure S3. Effects of the *seiv* mutations on ectopic xylem vessel formation in primary roots. (Supports Figure 2) Seven-day-old seedlings of Col-0, *VND7-VP16-GR*, *seiv3*, *seiv4*, *seiv6*, and *seiv9* were treated with DEX for 3 d and subsequently subjected to microscopy observation of root xylem tissues. Bar = 50 μm.

Supplementary Data. Phookaew et al. (2024). Active protein ubiquitination regulates xylem vessel functionality. Plant Cell.

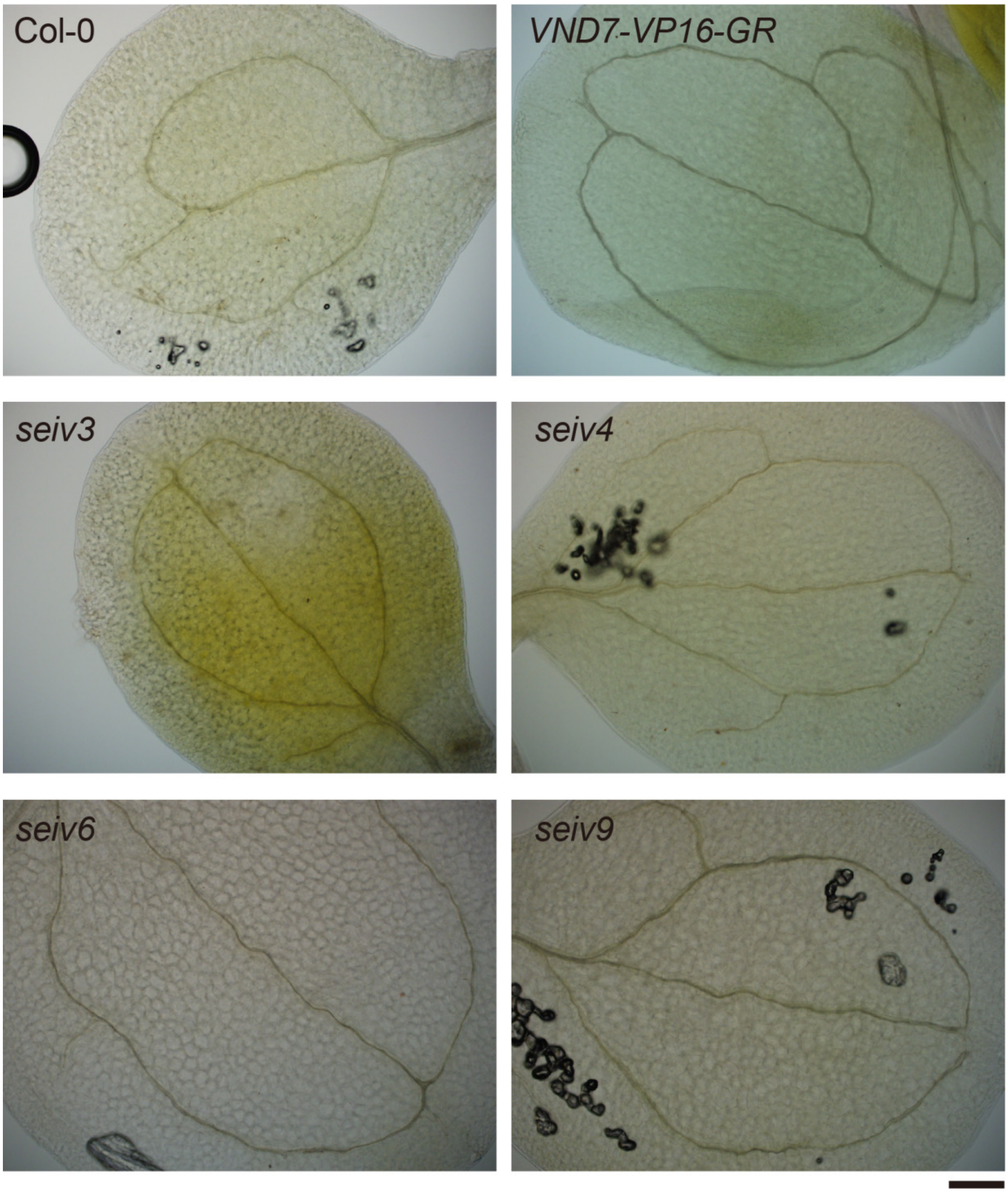

Supplementary Figure S4. Effect of the *seiv* mutations on endogenous xylem vessel formation in cotyledons. (Supports Figure 2) Seven-day-old seedlings of Col-0, *VND7-VP16-GR*, *seiv3, seiv4*, *seiv6*, and *seiv9* were subjected to microscopy observation of cotyledons. Bar = 200 μm.

Supplementary Data. Phookaew et al. (2024). Active protein ubiquitination regulates xylem vessel functionality. Plant Cell.

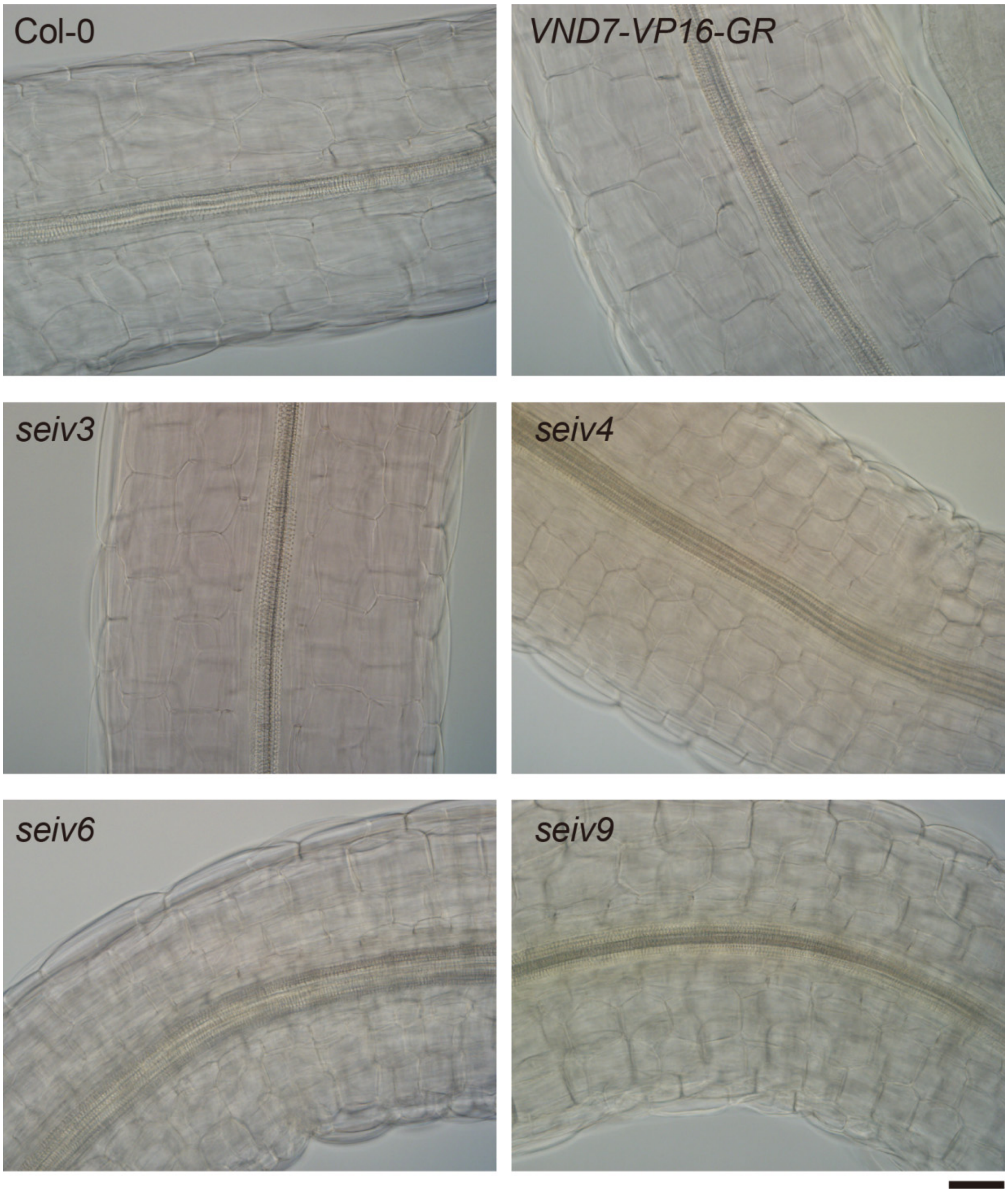

Supplementary Figure S5. Effect of the *seiv* mutations on endogenous xylem vessel formation in hypocotyls. (Supports Figure 2) Seven-day-old seedlings of Col-0, *VND7-VP16-GR*, *seiv3, seiv4*, *seiv6*, and *seiv9* were subjected to microscopy observation of hypocotyls. Bar = 50 μm.

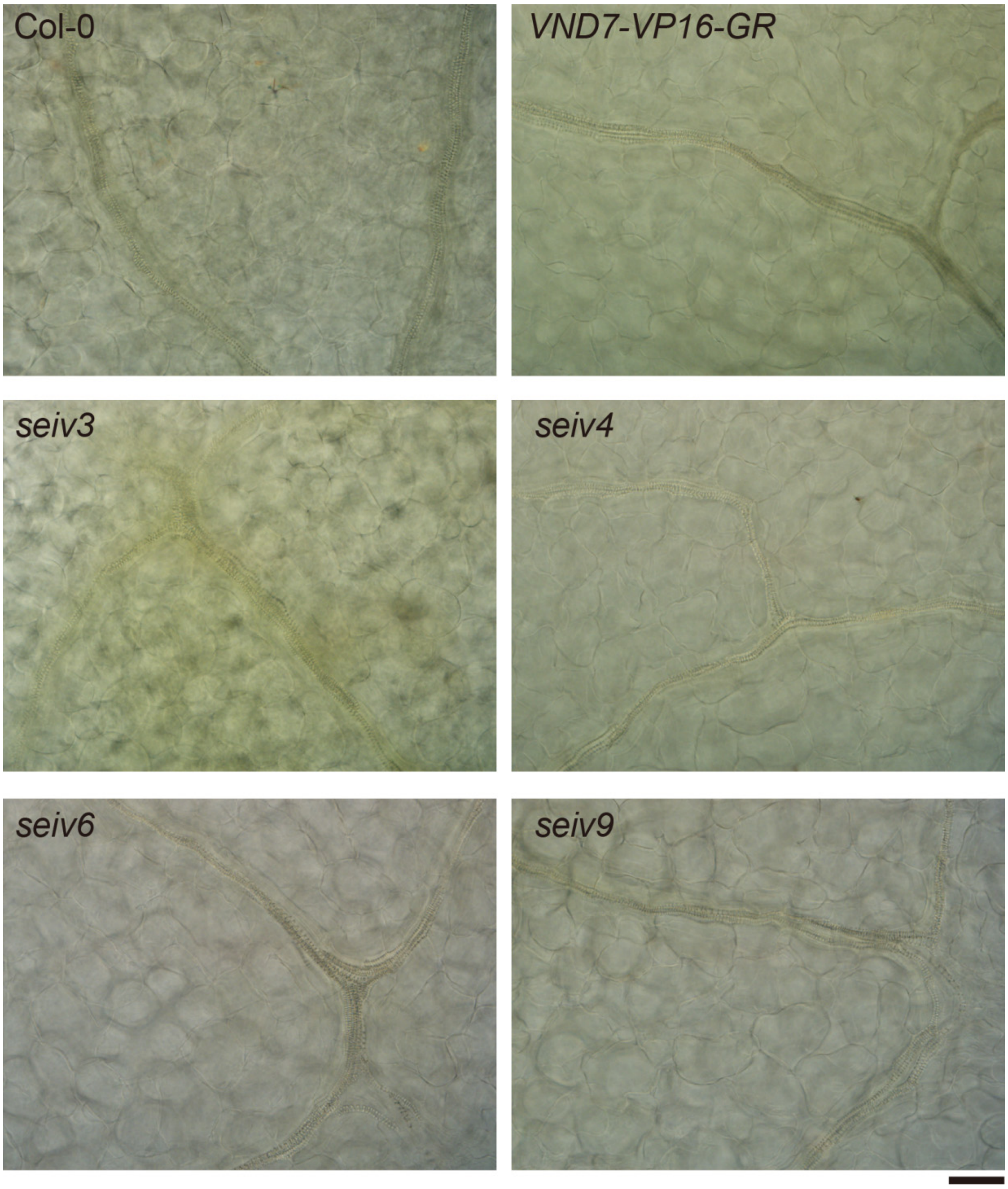

Supplementary Figure S6. Effect of the *seiv* mutations on endogenous xylem vessel formation in leaf veins. (Supports Figure 2) Seven-day-old seedlings of Col-0, *VND7-VP16-GR*, *seiv3, seiv4*, *seiv6*, and *seiv9* were subjected to microscopy observation of leaf veins. Bar = 50 µm.

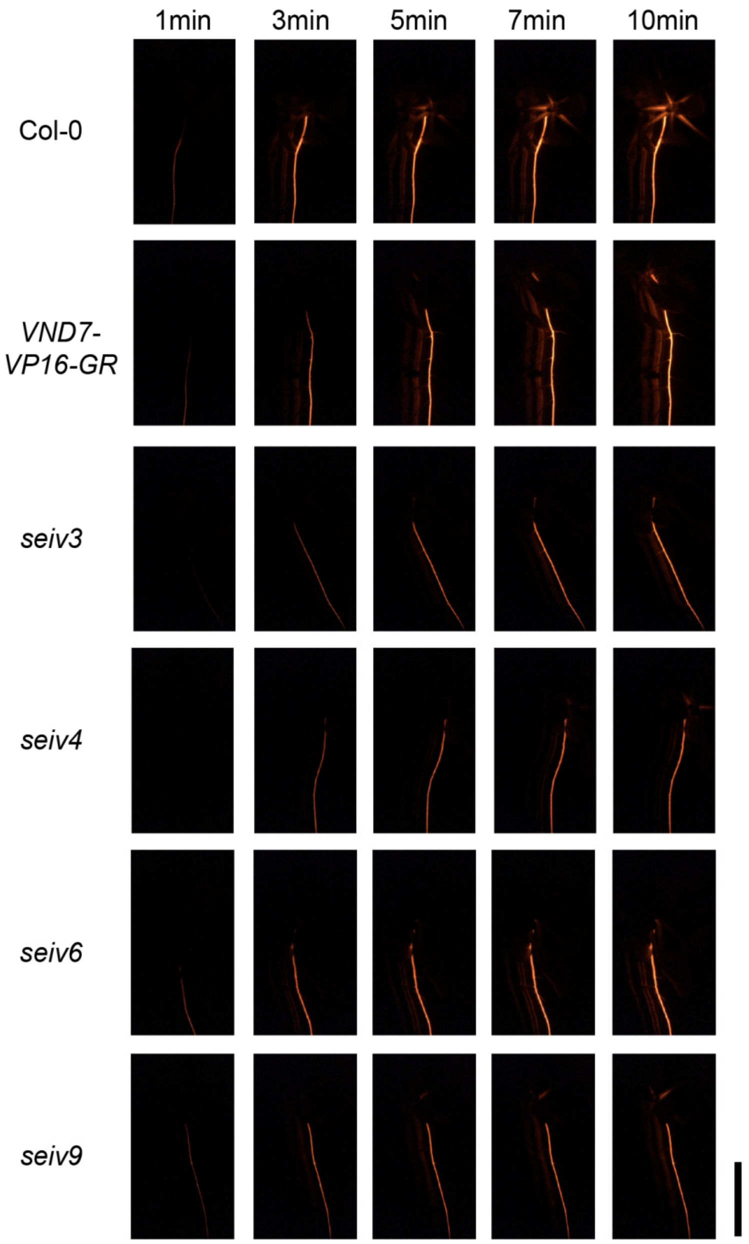

Supplementary Figure S7. Xylem transport ability is impaired in the *seiv* mutants. (Supports Figure 2) Fluorescence dye rhodamine was added at the excised tip of primary roots of 14-day-old Col-0, wild-type *VND7-VP16-GR*, and *seiv* seedlings. The fluorescent signal was observed every minute and recorded. Bars = 1 cm.

Supplementary Data. Phookaew et al. (2024). Active protein ubiquitination regulates xylem vessel functionality. Plant Cell.

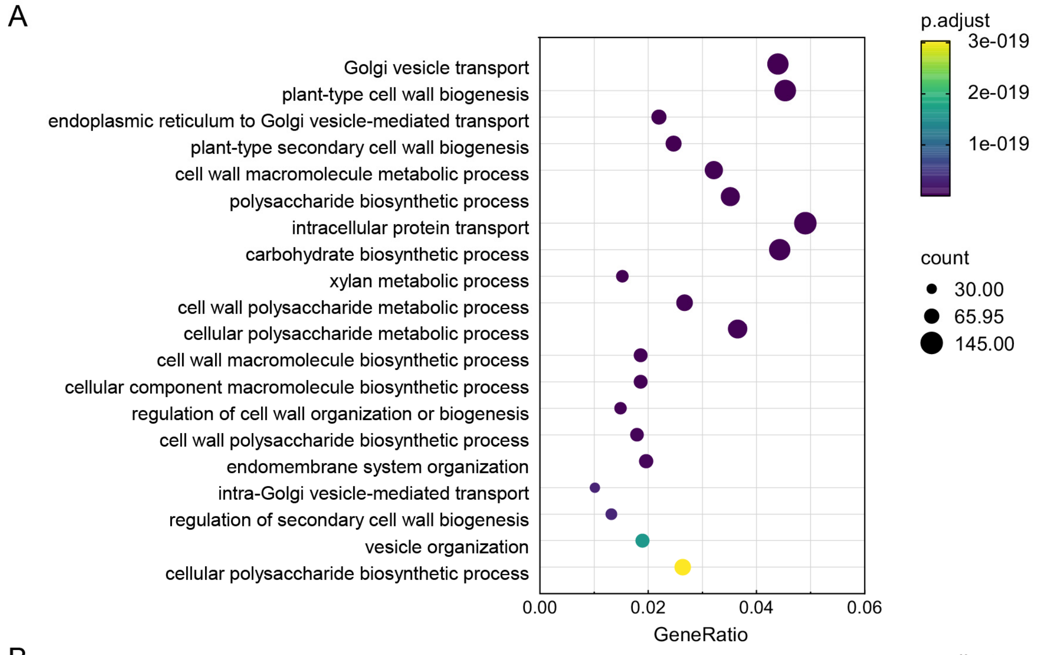

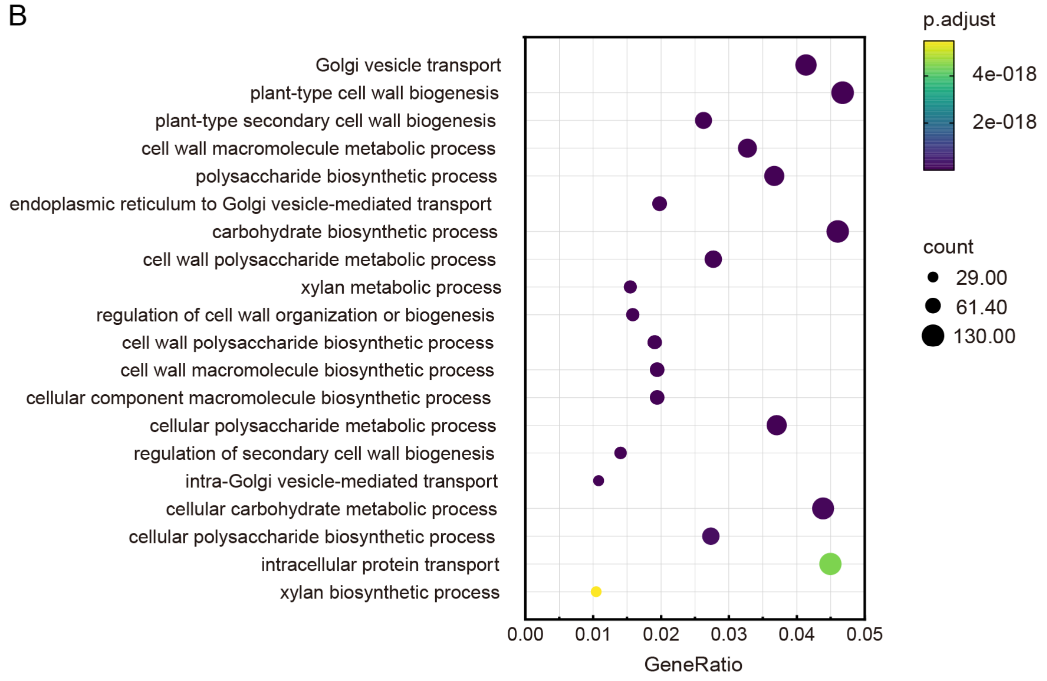

Supplementary Figure S8. Gene ontology (GO) term analysis for down-regulated genes in *seiv* mutants compared with wild-type *VND7-VP16-GR* under DEX treatment. (Supports Figure 3) Significantly-enriched top 20 GO terms are displayed with p. adjust values, containing gene number, and gene ratio of each GO term. The size of the circles indicates the fold enrichment of the GO terms. Results of *seiv3* and *seiv4* were shown in (A) and (B), respectively.

Supplementary Data. Phookaew et al. (2024). Active protein ubiquitination regulates xylem vessel functionality. Plant Cell.

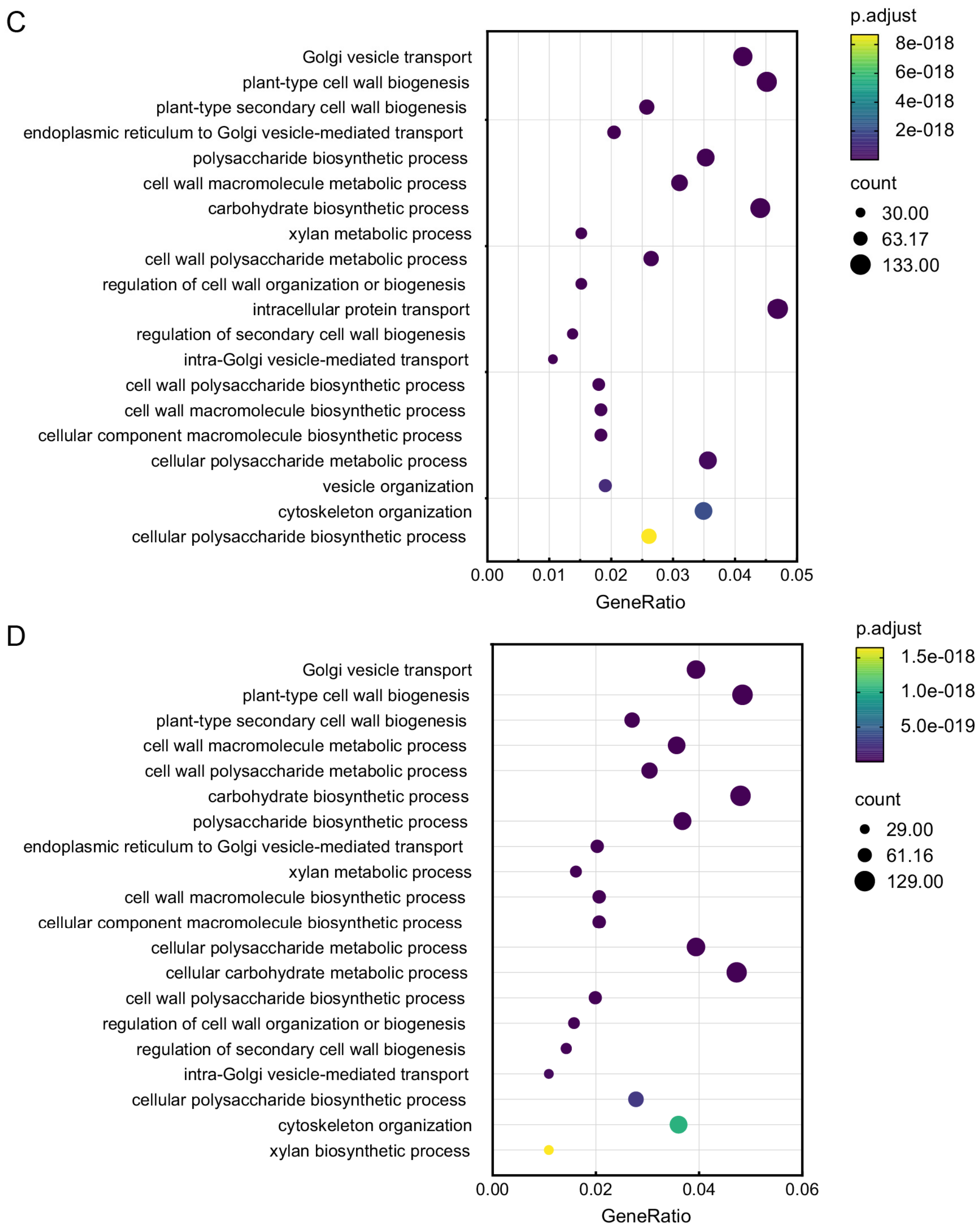

(continued from the previous page.) Results of *seiv6* and *seiv9* were shown in (C) and (D), respectively.

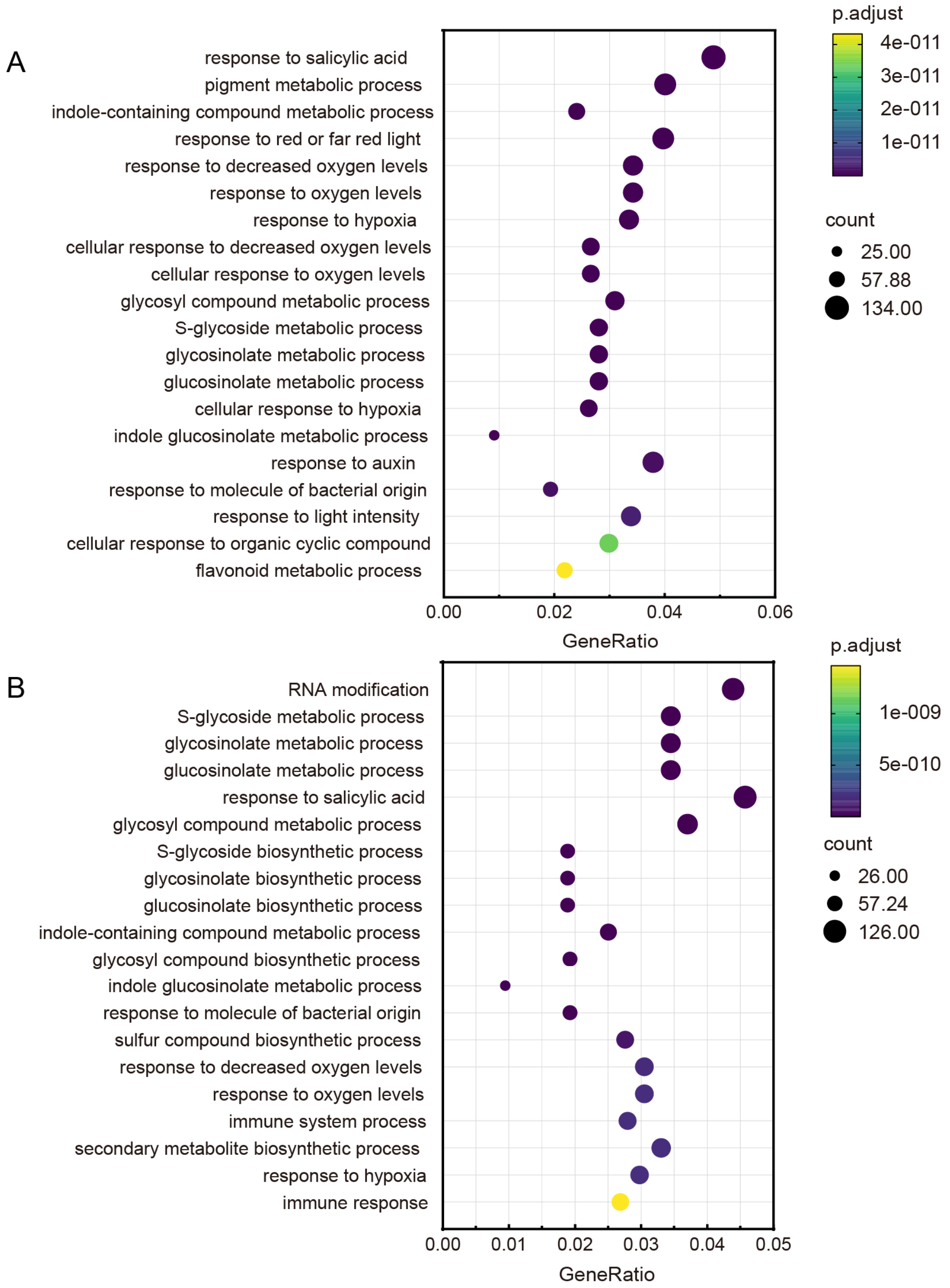

Supplementary Figure S9. Gene ontology (GO) term analysis for up-regulated genes in *seiv* mutants compared with wild-type *VND7-VP16-GR* under DEX treatment. (Supports Figure 3) Significantly-enriched top 20 GO terms are displayed with p. adjust values, containing gene number, and gene ratio of each GO term. The size of the circles indicates the fold enrichment of the GO terms. Results of *seiv3* and *seiv4* were shown in (A) and (B), respectively.

Supplementary Data. Phookaew et al. (2024). Active protein ubiquitination regulates xylem vessel functionality. Plant Cell.

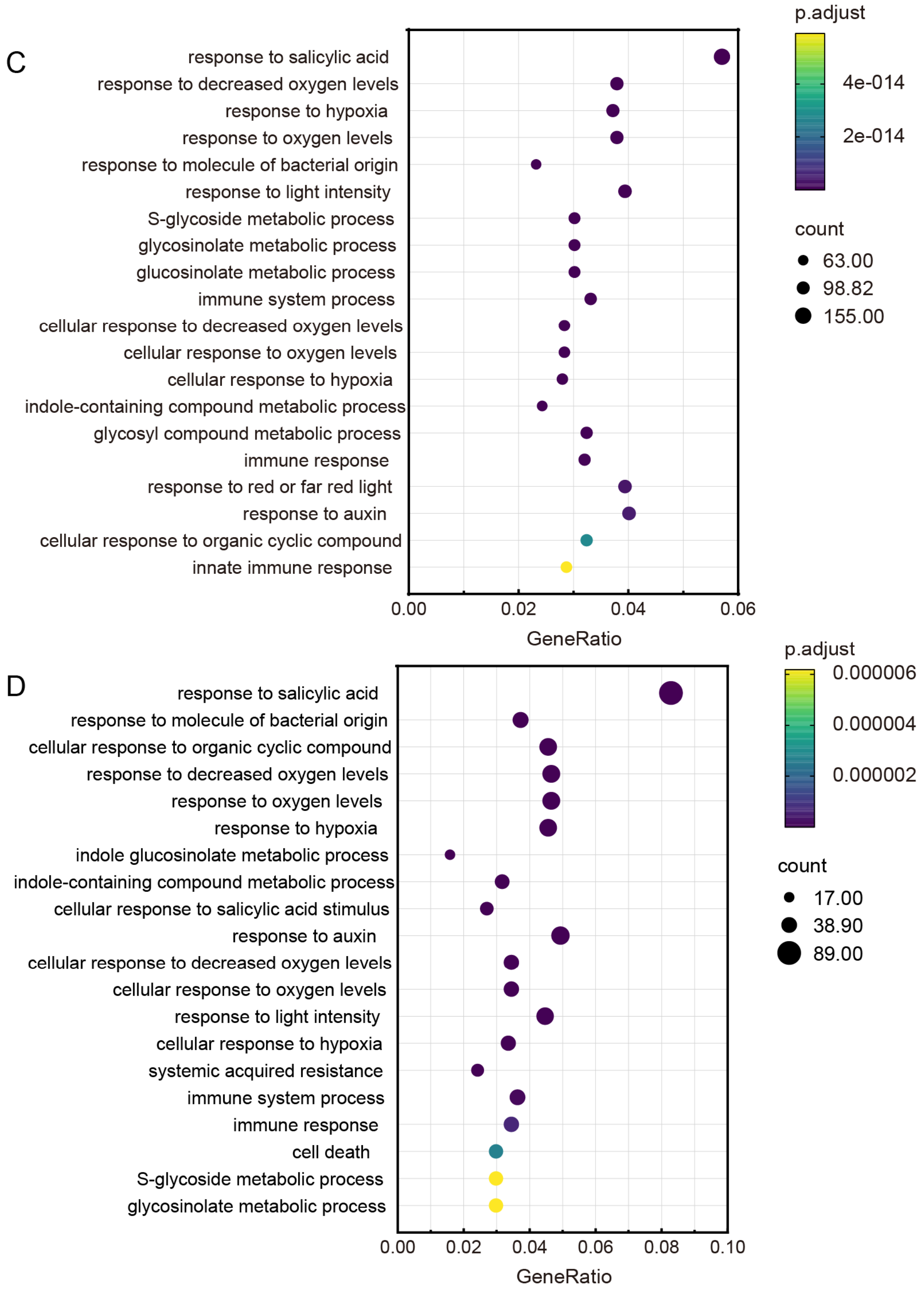

(continued from the previous page.) Results of *seiv6* and *seiv9* were shown in (C) and (D), respectively.

Supplementary Data. Phookaew et al. (2024). Active protein ubiquitination regulates xylem vessel functionality. Plant Cell.

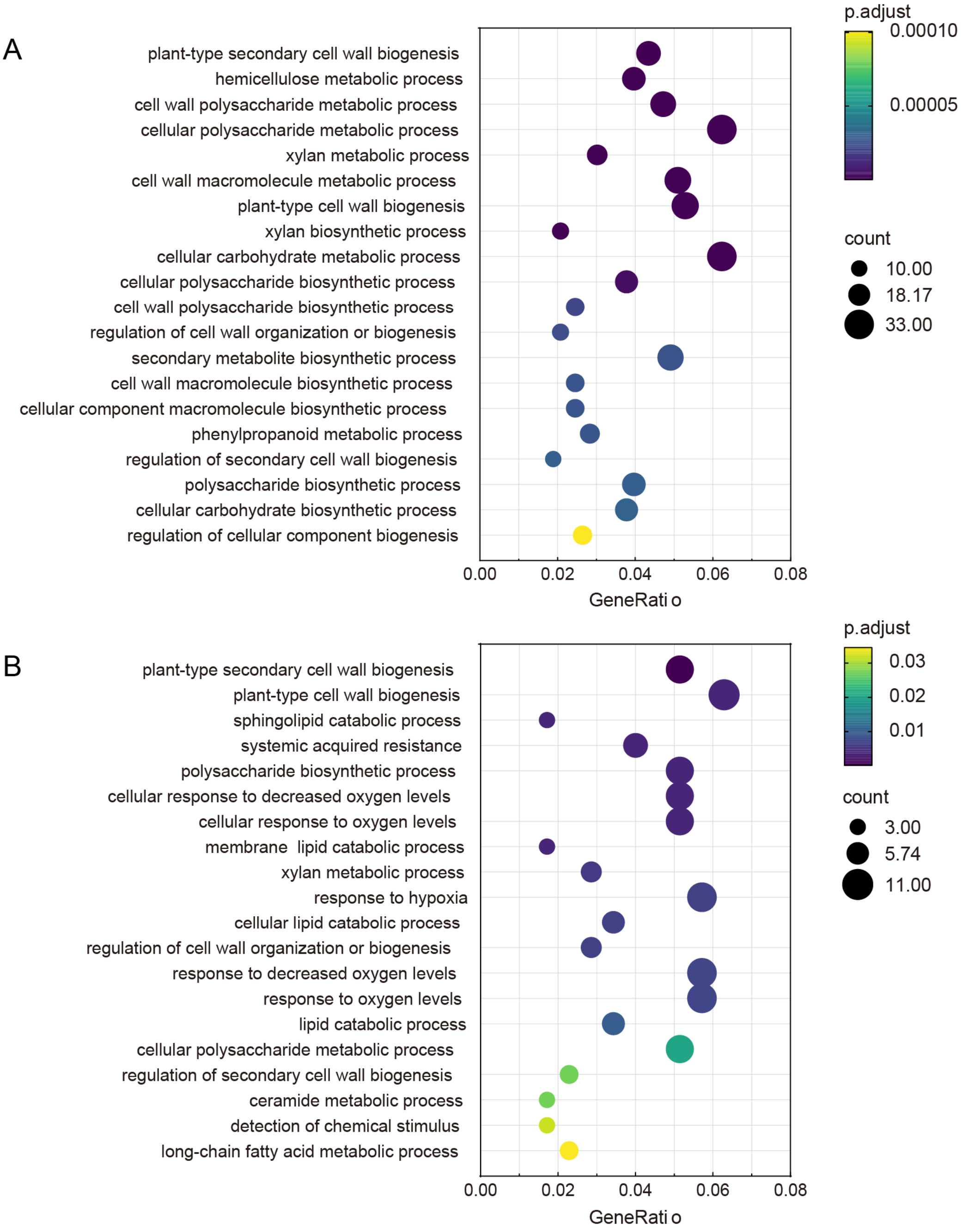

Supplementary Figure S10. Gene ontology (GO) term analysis for down-regulated genes in *seiv* mutants compared with wild-type *VND7-VP16-GR* under mock treatment. (Supports Figure 2 and 3) Significantly-enriched top 20 GO terms are displayed with p. adjust values, containing gene number, and gene ratio of each GO term. The size of the circles indicates the fold enrichment of the GO terms. Results of *seiv3* and *seiv4* were shown in (A) and (B), respectively.

Supplementary Data. Phookaew et al. (2024). Active protein ubiquitination regulates xylem vessel functionality. Plant Cell.

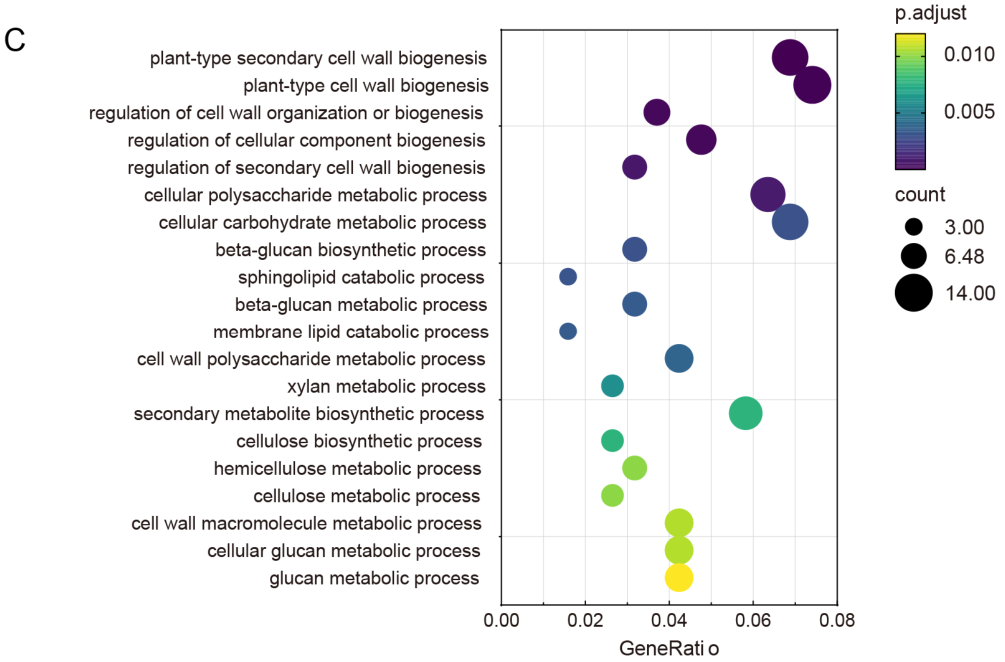

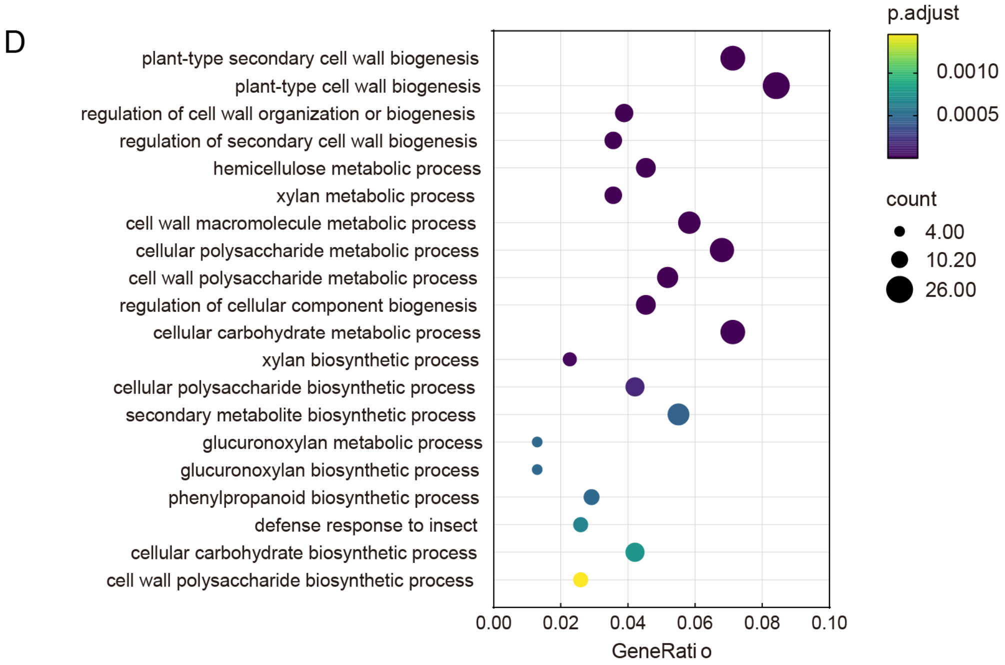

(Continued from the previous page.) Results of *seiv6* and *seiv9* were shown in (C) and (D), respectively.

Supplementary Data. Phookaew et al. (2024). Active protein ubiquitination regulates xylem vessel functionality. Plant Cell.

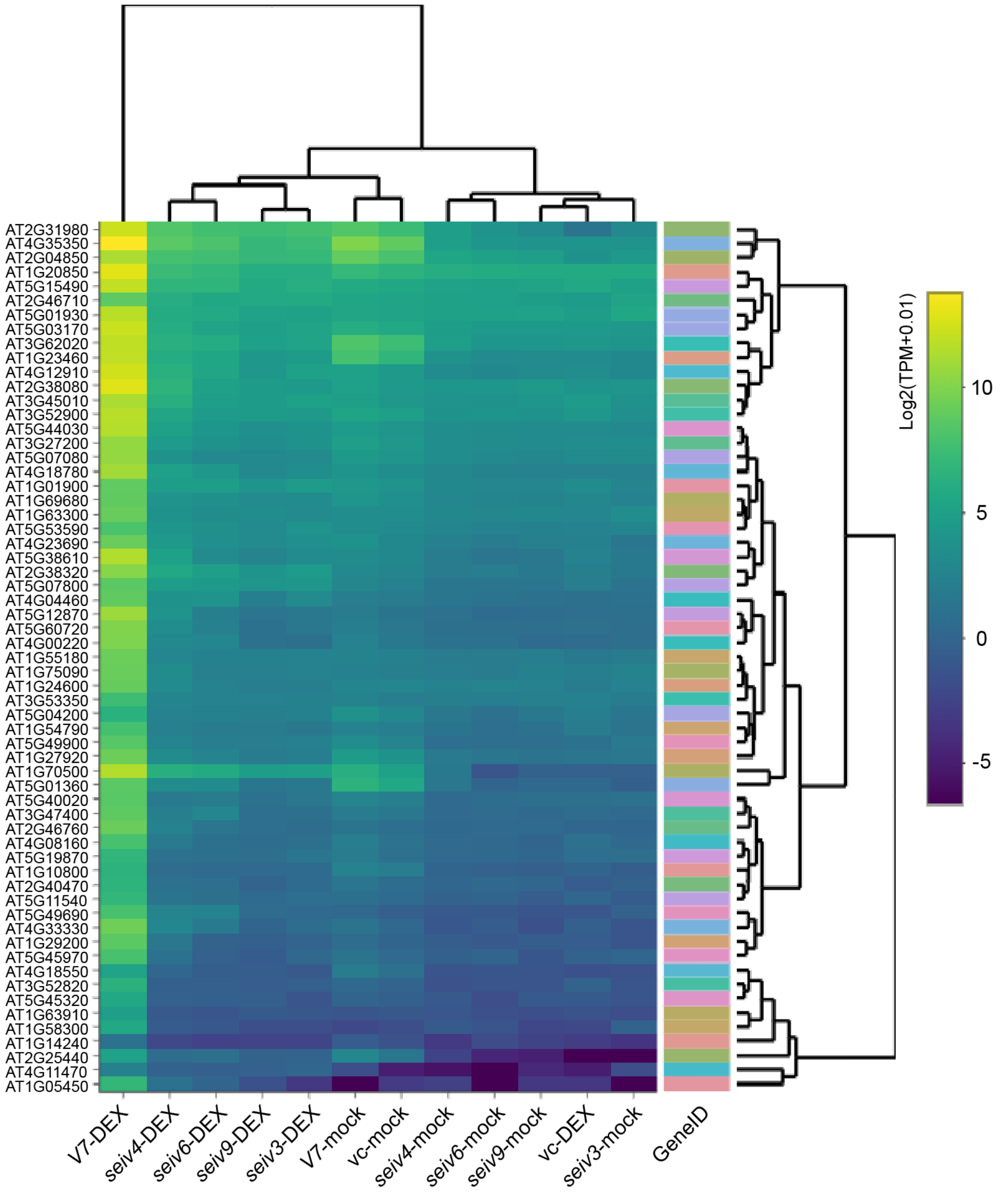

Supplementary Figure S11. Expression levels of VND7-downstream genes in the vector control, wild-type *VND7-VP16-GR*, and *seiv* seedlings. (Supports Figure 2 and 3) Heatmap showing the significance of expression levels of VND7-downstream genes in the wild-type *VND7-VP16-GR* (*V7*), vector control (vc), and *seiv3*, *seiv4*, *seiv6*, and *seiv9* mutants with and without DEX treatment.

Supplementary Data. Phookaew et al. (2024). Active protein ubiquitination regulates xylem vessel functionality. Plant Cell.

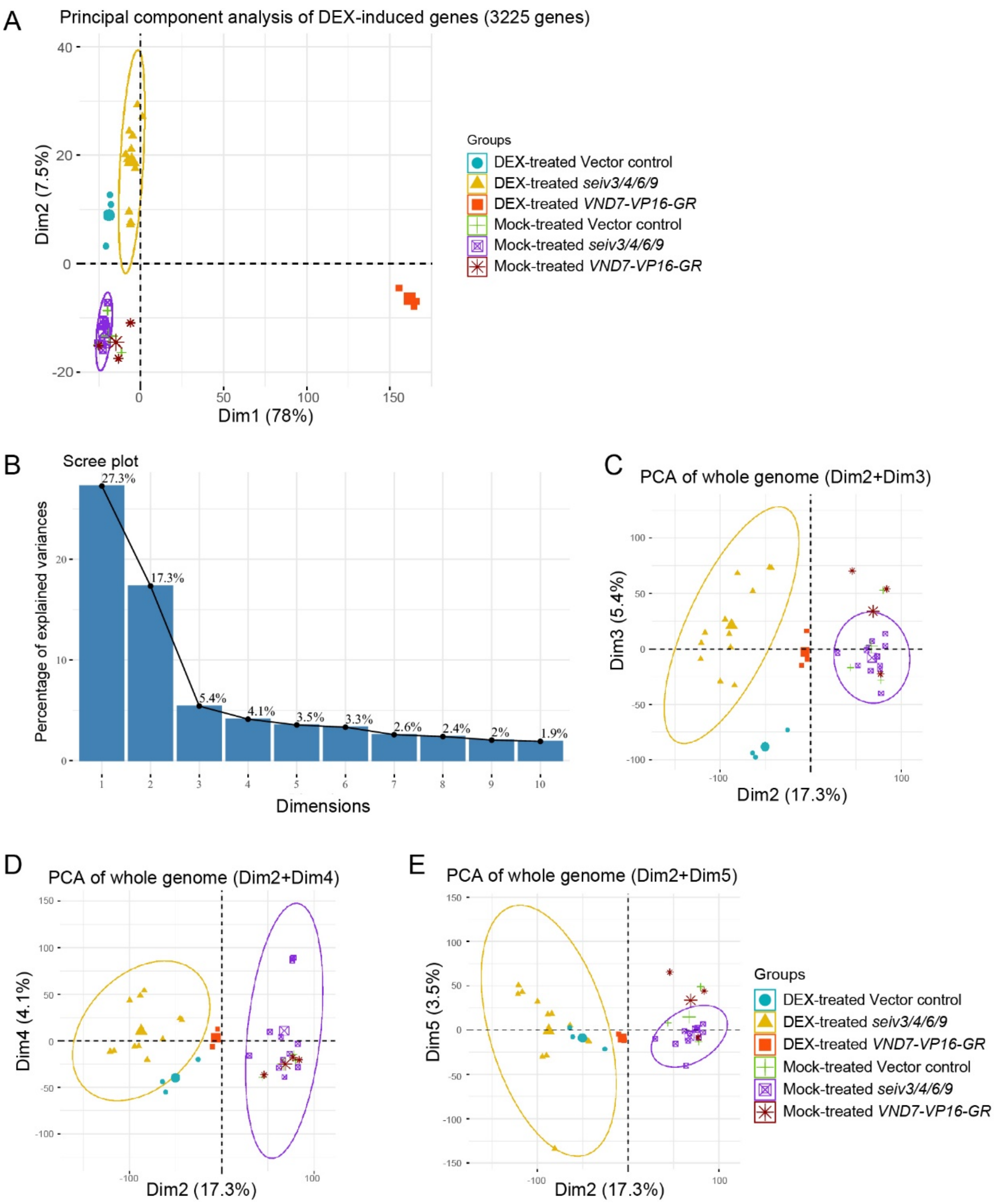

Supplementary Figure S12. Principal component analysis (PCA) of RNA-seq data of the wild-type *VND7-VP16-GR*, vector control, and *seiv* mutants. (Supports Figure 3) Seven-day-old seedlings were treated with DEX, and then collected after 6 h of treatment. Extracted total RNAs were subjected to RNA-seq analysis. Three replicates were analyzed for each treatment condition. (A) PCA for the genes upregulated by the

DEX treatment. Horizontal and vertical axes indicate the PC1 and PC2 values, respectively. (B) Scree plot in the PCA of the whole transcriptome. The scree plot displays the eigenvalues of each principal component (PC) against the number of components. (C-E) PCA for the whole transcriptome with the axes of Dim2 and Dim 3/4/5, respectively.

Supplementary Data. Phookaew et al. (2024). Active protein ubiquitination regulates xylem vessel functionality. Plant Cell.

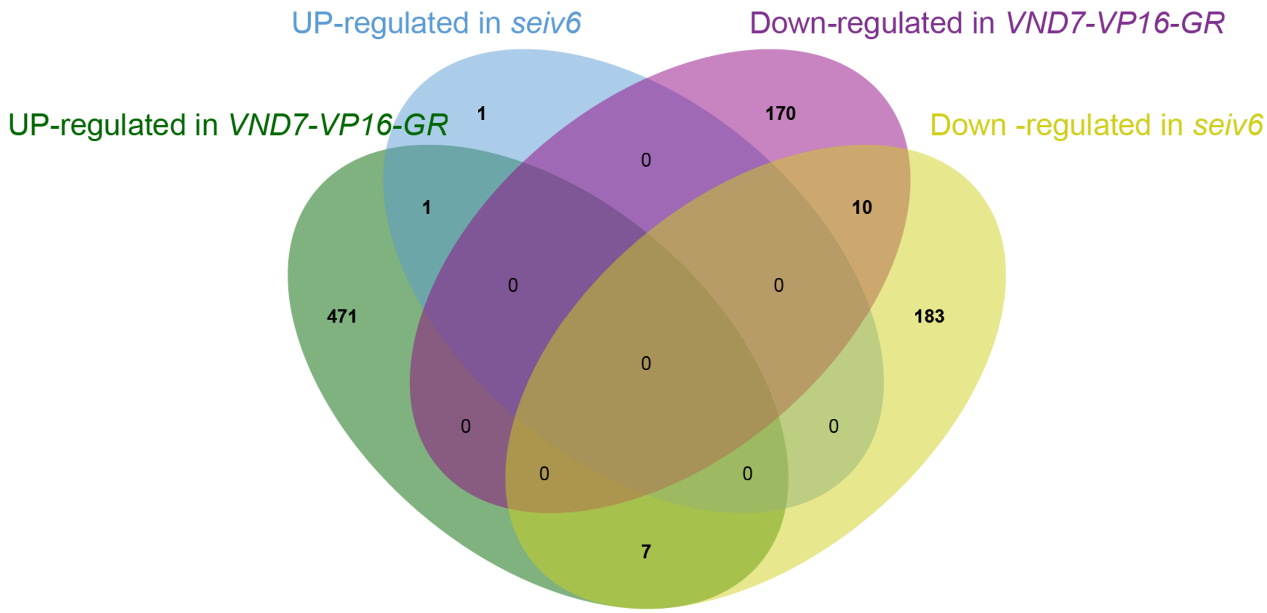

Supplementary Figure S13. Multiple Venn diagrams of up- and down-regulated ubiquitination events in response to VND7 induction in wild-type *VND7-VP16-GR* and *seiv6*. (Supports Figure 5). Multiple Venn diagrams showing the common and specific up- and downregulated ubiquitination events in response to VND7 induction in *VND7-VP16-GR* and *seiv6*, respectively.

Supplementary Data. Phookaew et al. (2024). Active protein ubiquitination regulates xylem vessel functionality. Plant Cell.

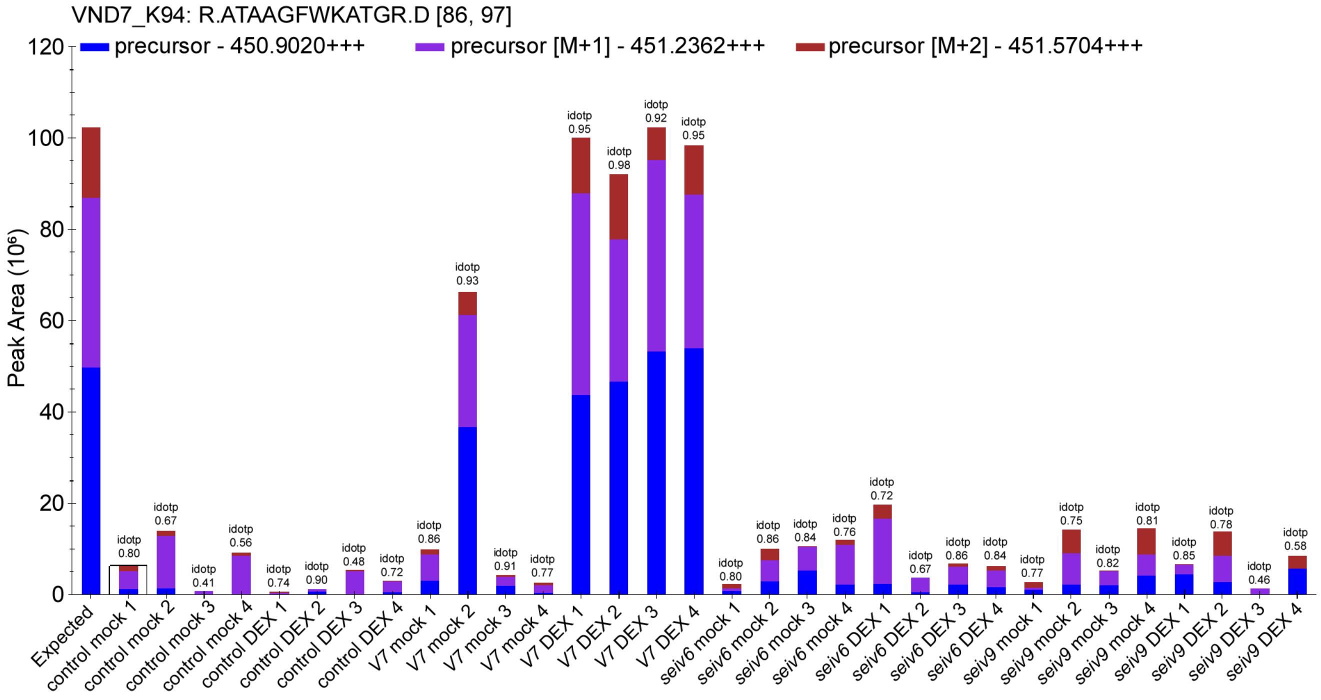

Supplementary Figure S14. Ubiquitination level on VND7 during xylem vessel formation. (Supports Figure 6) Replicates comparing the ubiquitination levels of three lysine residues (K94/K105/K260) in VND7 during xylem vessel cell differentiation.

Supplementary Data. Phookaew et al. (2024). Active protein ubiquitination regulates xylem vessel functionality. Plant Cell.

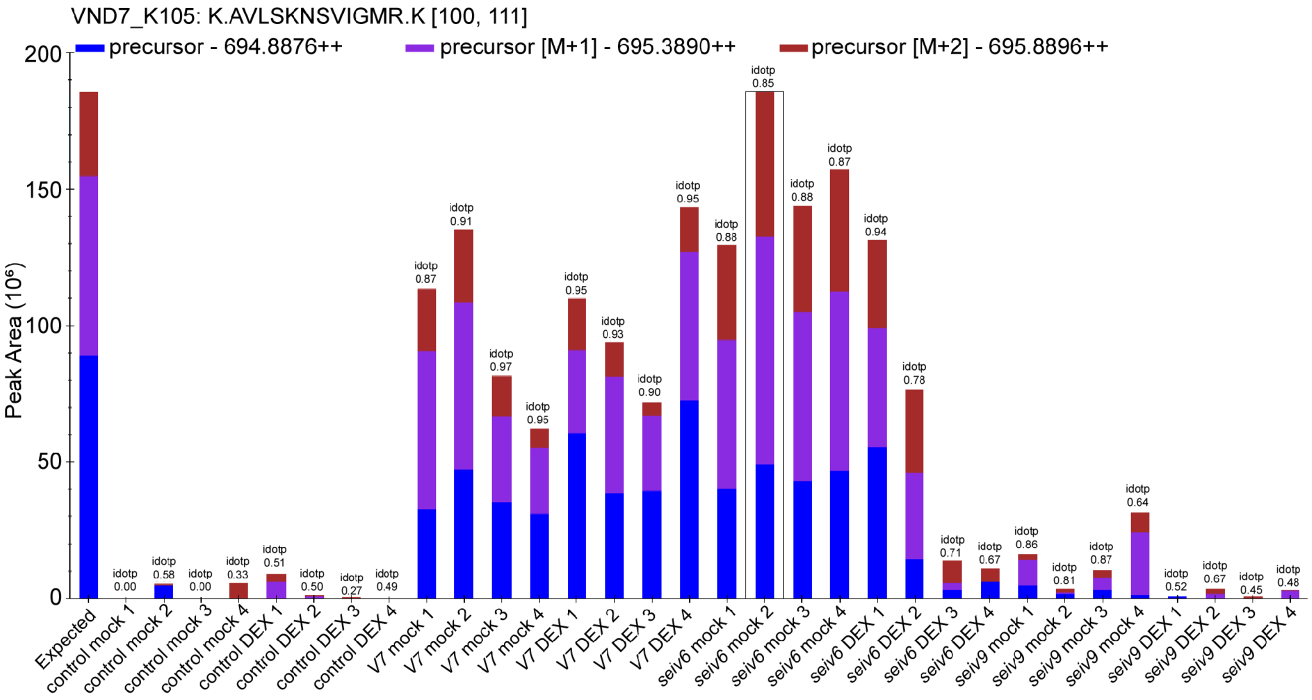

(Continued from the previous page.) Replicates comparing the ubiquitination levels of three lysine residues (K94/K105/K260) in VND7 during xylem vessel cell differentiation

Supplementary Data. Phookaew et al. (2024). Active protein ubiquitination regulates xylem vessel functionality. Plant Cell.

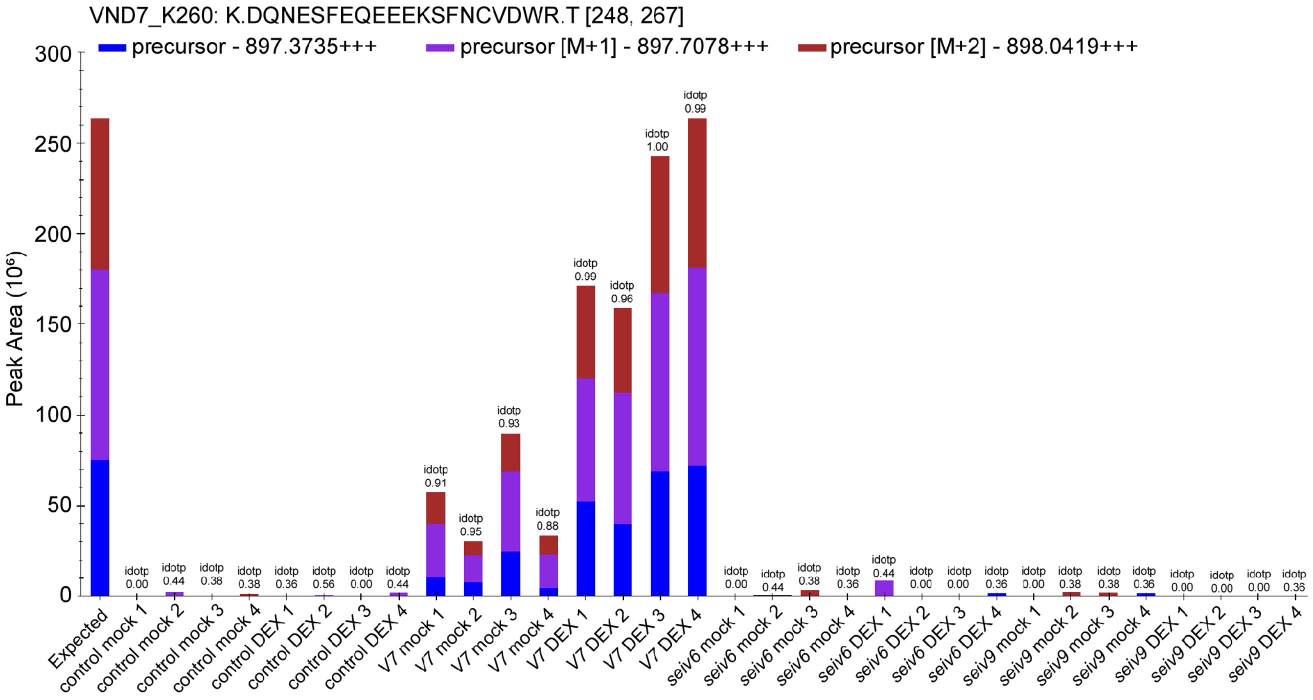

(Continued from the previous page.) Replicates comparing the ubiquitination levels of three lysine residues (K94/K105/K260) in VND7 during xylem vessel cell differentiation

Supplementary Data. Phookaew et al. (2024). Active protein ubiquitination regulates xylem vessel functionality. Plant Cell.

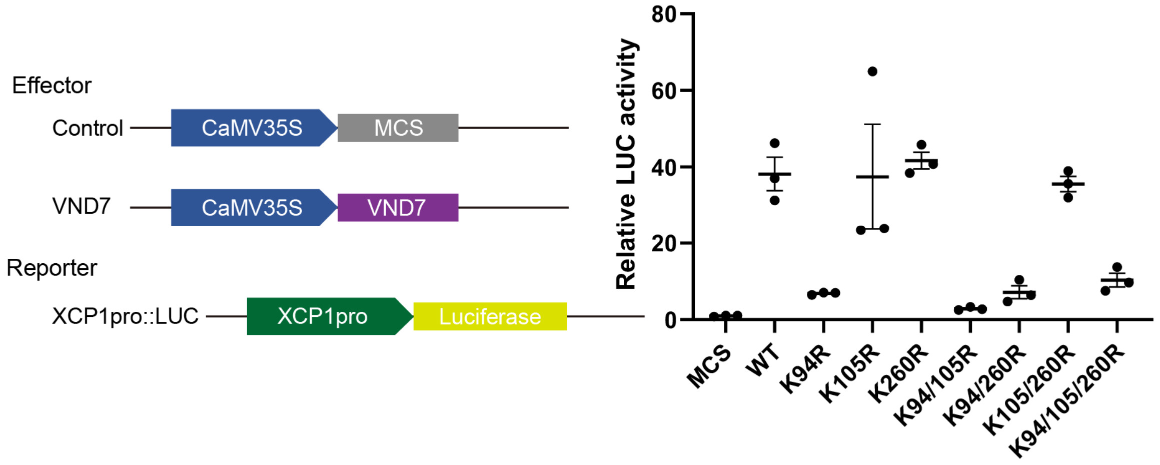

Supplementary Figure S15. Reduced transcriptional activity of VND7 when K94 was substituted with Arg. (Supports Figure 6) The effector construct consists of DNA sequences encoding the full-length wild-type VND7 (WT) or mutated VND7 (K94R, K105R, K260R, K94R/K105R, K94R/K260R, K105R/K260R, K94R/K105R/K260R) under the control of the CaMV 35S promoter. The multi cloning site (MCS) sequence fused with the CaMV 35S promoter was used as a transfection control. The reporter construct consists of a luciferase (LUC) gene driven by the *XCP1* promoter fragment X1E1 (the promoter region at −148 to −96 bp; Yamaguchi et al., 2011). Transient reporter assays to elucidate the effects of mutations at Lys94, Lys105, and Lys260 of VND7 (right). All LUC activities were normalized to Rluc activity. Data are means ± SE (n = 3). The results are shown relative to the control effector (MCS = 1.0). Asterisks indicate significant differences (Student's t-test; p-value<0.001) from wild-type VND7 (WT).

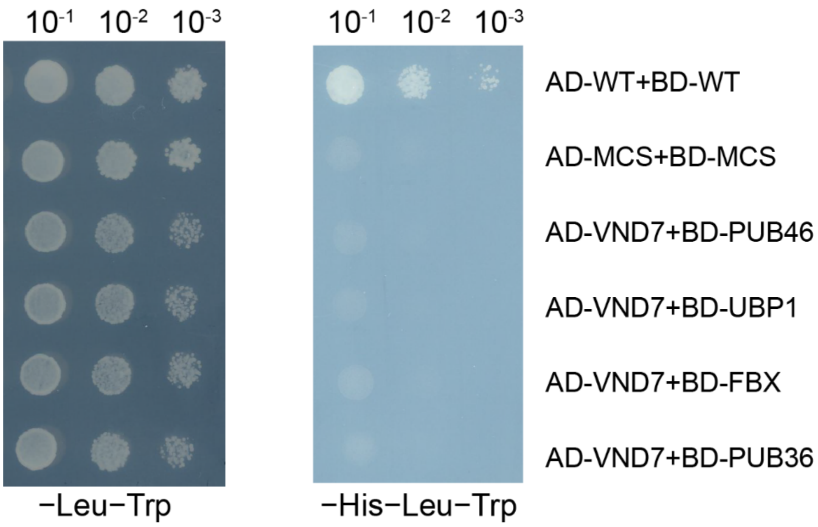

Supplementary Figure S16. VND7 does not directly interact with SEIV proteins. (Supports Figure 6) VND7 did not exhibit interaction with SEIV proteins in Y2H assays. Serial dilutions from relevant yeast colonies were spotted onto synthetic defined (SD) medium (–Leu–Trp or –His–Leu–Trp). The vectors pAD-GAL4 (AD-WT) and pBD-GAL4 (BD-WT) were used as positive controls while pAD-GAL4 (AD-MCS) and pBD-GAL4 (BD-MCS) were used as negative controls. Similar results were confirmed by 3 independent experiments.

Supplementary Data. Phookaew et al. (2024). Active protein ubiquitination regulates xylem vessel functionality. Plant Cell.

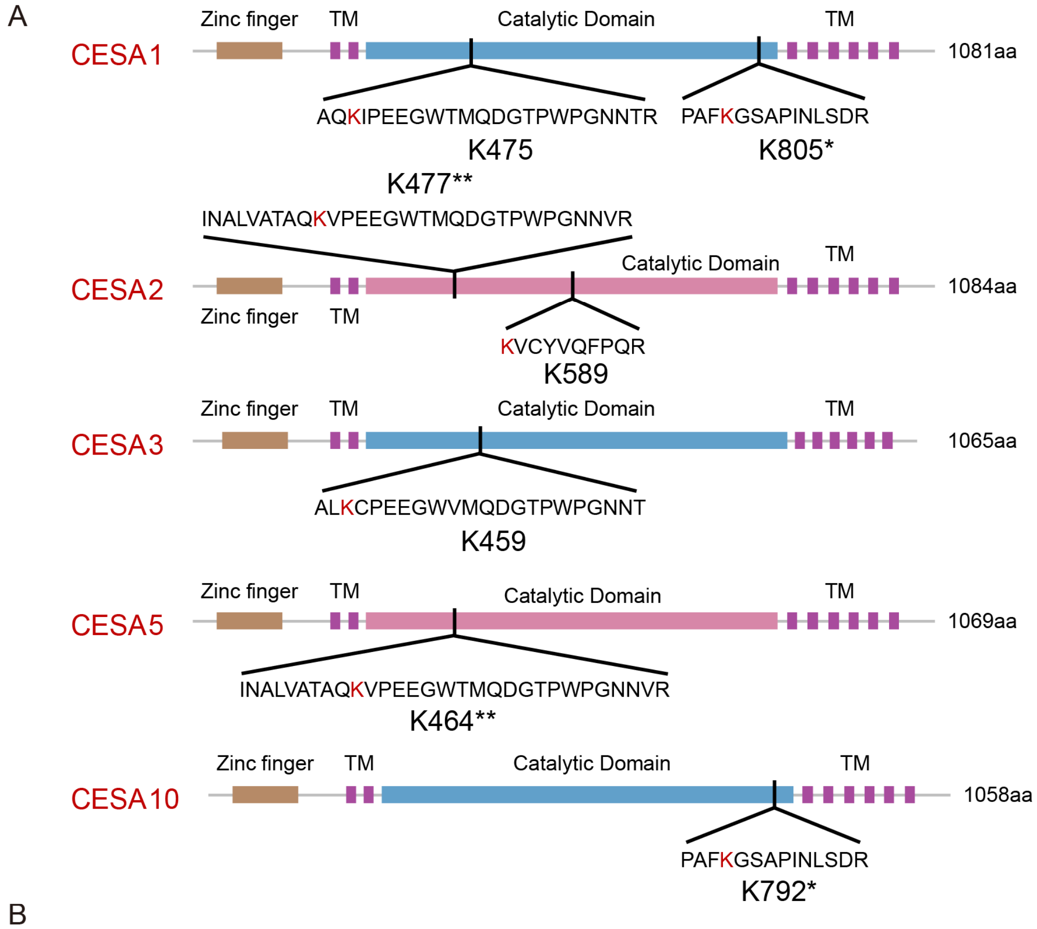

B

CESAs	Lysine position	*VND7-VP16-GR*		vector control		*seiv6*		*seiv9*	
		Mock	DEX	Mock	DEX	Mock	DEX	Mock	DEX
CESA1	K475	+	++	+	+	+	+	ND	ND
CESA2	K589	ND	++	ND	ND	ND	ND	ND	ND
CESA3	K459	+	++	ND	ND	+	+	+	+
CESA5; CESA2	K464; K477	+	++	+	+	+	ND	ND	ND
CESA10; CESA1	K792; K805	ND	++	ND	ND	ND	ND	ND	ND

Supplementary Figure S17. Ubiquitination events detected in CESA1, CESA3, and CESA6-like (CESA2, CESA5), and CESA10 upon VND7 induction. (Supports Figure 4) (A) Diagram of the PCW CESAs locating the identified ubiquitination sites relative to catalytic domain. The MS-identified peptide sequence bearing each Ub footprint is shown with the modified lysine colored in red. *Overlapping ubiquitinated peptide: CESA2 (K477) and CESA5 (K464); **Overlapping ubiquitinated peptide: CESA1 (K805) and CESA10

(K792). (B) Detection and comparison of ubiquitinated lysines within PCW-specific CESAs in vector control, wild-type *VND7-VP16-GR*, *seiv6*, and *seiv9* under DEX- and mock-treated conditions. “ND” indicates no detection of ubiquitination events, and “+” indicates the detection of ubiquitinated events. "++" indicates a significantly higher level of ubiquitination detected compared to the same peptides across other lines and conditions.

Supplemental Table S1. Indicators of light quality

plant growth chamber (seedlings grown on medium)

Indicator	
LUX	1979.0±422.77 lm/m^2
PPFD (400-700nm)	25.17±5.47 $\mu mol/m^2/s$
PFD (380-780nm)	25.91±5.64 $\mu mol/m^2/s$
PFD-UV (380-400nm)	0.04±0.01 $\mu mol/m^2/s$
PFD-B (400-500nm)	6.47±1.45 $\mu mol/m^2/s$
PFD-G (500-600nm)	11.25±2.35 $\mu mol/m^2/s$
PFD-R (600-700nm)	7.44±1.67 $\mu mol/m^2/s$
PFD-FR (700-780nm)	0.71±0.17 $\mu mol/m^2/s$

Plant growth room (seedlings grown in soil)

Indicator	
LUX	914.34±435.82 lm/m^2
PPFD (400-700nm)	12.85±6.10 $\mu mol/m^2/s$
PFD (380-780nm)	13.43±6.40 $\mu mol/m^2/s$
PFD-UV (380-400nm)	0.07±0.05 $\mu mol/m^2/s$
PFD-B (400-500nm)	3.90±1.91 $\mu mol/m^2/s$
PFD-G (500-600nm)	6.08±2.94 $\mu mol/m^2/s$
PFD-R (600-700nm)	2.87±1.25 $\mu mol/m^2/s$
PFD-FR (700-780nm)	0.51±0.25 $\mu mol/m^2/s$
