# Supplementary material for: Active protein ubiquitination regulates xylem vessel functionality
Source: Plant Cell. 2024 Aug 2;36(9):3298–317. doi: 10.1093/plcell/koae221 (PMC11371170; doi:10.1093/plcell/koae221)
Supplement: koae221_Supplementary_Data [file tpc.00076.2024-s07.pdf › tpc.00076.2024-s07.pdf]

THE PLANT CELL

Active protein ubiquitination regulates xylem vessel functionality

Pawittra Phookaew, Ya Ma, Takaomi Suzuki, Sara Stolze, Anne Harzen, Ryosuke Sano, Hirofumi Nakagami, Taku Demura, and Misato Ohtani

Review Timeline:

Submission Date:	26-Jan-2024
Editorial Decision:	09-Mar-2024
Revision Received:	30-Apr-2024
Editorial Decision:	31-May-2024
Revision Received:	18-Jun-2024
Accepted:	25-Jun-2024

Dr. Misato Ohtani
Tokyo Daigaku - Kashiwa Campus
Kashiwa, Chiba 277-8562
Japan

Dear Dr. Ohtani:

We have received reviews of your manuscript entitled "Active protein ubiquitination regulates xylem vessel functionality." Thank you for submitting your best work to The Plant Cell. The editorial board agrees that the work you describe is substantive, falls within the scope of the journal, and may become acceptable for publication, pending revision and potential re-review.

Please respond to each of the referees comments in a revised version of the manuscript.

Please contact us if there are ambiguous comments or if you wish to discuss the revision.

Given the nature of the comments, we are offering you 60 days from when we have issued this decision to complete the revision. If a revision is not returned within this time frame, and if you have not been granted an extension, we will withdraw the manuscript, which will leave you free to submit the work elsewhere. If you need an extension, we encourage you to contact us at any point before the 60 days have passed.

When you are ready to submit the revised version, please upload a highlighted copy that indicates all changes made in response to the editor and reviewer recommendations. Include an itemized list of all changes made in response to each of the reviewer's suggestions in the "Response to Reviewers" section; please note that reviewers do not have access to your cover letter, nor was this decision letter shared with them.

Thank you for the privilege of reviewing your work. We look forward to receiving your revised manuscript.

Sincerely,

The Plant Cell Board of Editors

Please note the following:

-The Plant Cell now requires authors to complete and submit an author revisions checklist upon submission of a revised manuscript. The aim of the checklist is to aid authors in preparing a high-quality manuscript, facilitate the review and assessment of revised manuscripts, and help to ensure that journal standards are maintained across the board. If your manuscript is accepted, the completed checklist will be published as supplemental material attached to the article online. Please download a copy of the checklist (pdf fillable form) at this link, for submission with your revised manuscript: https://tpc.msubmit.net/html/Author_Revisions_Checklist.pdf.

-Supplemental materials should be restricted to large datasets and tables, presentation of replicates, and validation of reagents, methods, or genotypes. Any data that are used to support the major claims must be in the main manuscript. Supplemental figure legends must indicate what figure in the main manuscript is supported by the supplemental data presented. Please justify how each of the supplemental figures meet the criteria.

-Sampling methods and nature of "biological replicates" should be described precisely (i.e. different plants, parts of plants, pooled tissue, independent pools of tissue, sampled at different times, etc), along with a clear description of and rationale for any statistical analyses conducted. The reader should know exactly what was sampled; what forms the basis of the calculation of any means and statistical parameters reported. This is also necessary to ensure that proper statistical analysis was conducted.

-Want to add this revision deadline to your calendar? Click below!

-- Reviewer comments:

Reviewer #1 (Comments for the Author):

This manuscript reports several suppressor mutants of VND7-inducible xylem vessel cell differentiation. Thanks to the authors' contribution, VND7 is a relatively well characterized master regulator of xylem vessel differentiation. Proper function of xylem vessel is critical for the plant's growth and survival. Identification and characterization of the mutants lead to multiple testable hypotheses. While the findings described in this report are significant contribution to expanding our knowledge in plant science, the overall impression of this report is that the results are mostly descriptive, rather than the outcome from hypothesis-driven research. I have no doubt that these findings are useful in further study of the role of posttranslational regulation of xylem vessel differentiation and function. Therefore, additional studies (e.g. genetic complementation) to test the hypotheses that the authors propose will strengthen this manuscript.

Additional points to improve this manuscript:

The authors suggested that "the dominant seiv mutations preferentially affect VND7-induced xylem vessel cell differentiation in aboveground tissues." Yet, they used primary roots to examine the impacts of the seiv mutations on endogenous xylem vessel cell differentiation. They need to provide rationale for their choice.

The authors did not detect any differences between Col-0, wild-type VND7-VP16-GR, and the seiv mutants in terms of xylem vessel element formation as well as in the number or size of xylem vessels or SCW deposition. Then, they performed xylem performance assay. Again, providing their rationale for the assay will be helpful for readers to understand why they carried out the xylem performance assay when no xylem vessel element formation nor SCW deposition between the control and mutants. Furthermore, the xylem performance assay indicated that xylem vessel function is impaired in the seiv mutants despite no difference in the xylem vessel morphologically. I would like to see the authors' take on this finding in the Discussion.

The RNA-seq data identified many differentially expressed genes, including 61 VND7 target genes, indicating the unique effects of the seiv mutations on transcriptomic changes in response to DEX-induced xylem vessel formation. How would the authors reconcile this with the earlier observations of no differences in xylem element formation between the control and the mutants?

Reviewer #3 (Comments for the Author):

The article is well-written, with detailed analyses and a clear presentation of results, although rearranging them to maintain flow would enhance readability. It delineates the pivotal role of the NAC transcription factor VASCULAR-RELATED NAC-DOMAIN7 (VND7) as a master regulator of xylem vessel cell differentiation in Arabidopsis (Arabidopsis thaliana). Additionally, it characterises seiv mutants (suppressors of ectopic xylem vessel cell differentiation induced by VND7), revealing decreased expression of downstream genes and abnormal xylem transport activity. Upon VND7 induction, ubiquitination levels are modulated, impacting xylem vessel activity. The manuscript further elucidates the role of ubiquitination in regulating VND7 and its effect on xylem vessel function.
The study is exciting, and it is in an area that attracts a lot of interest. The findings presented in this manuscript suggest the potential involvement of one of the SEIV proteins in the ubiquitination of VND7. Despite the significant potential to elucidate the mechanisms underlying VND7 regulation, the authors did not directly test this hypothesis. As a suggestion to strengthen the manuscript, it would be intriguing to investigate whether VND7 is ubiquitinated or deubiquitinated by one of the SEIV proteins. This exploration could provide valuable insights into the regulatory mechanisms governing VND7 activity and contribute to a deeper understanding of xylem vessel cell differentiation.
Additional comments:
Line 120: To validate the point mutation in AT5G18320 (seiv3), AT2G32780 (seiv4), AT5G39250 (seiv6), and AT3G61390 (seiv9), the authors introduced genomic fragments of PUB46, UBP1, FBX, and PUB36 containing sequences of the coding sequence with the corresponding point mutations into the wild-type VND7-VP16-GR background. Can the authors explain why they did not complement Arabidopsis mutants for the selected genes with the coding sequence with and without the corresponding point mutations? This would validate that only single mutations in these genes are responsible for the suppressed xylem vessel cell differentiation. Have the Arabidopsis mutants for these genes suppressed xylem vessel cell differentiation?
Line 164: The authors should specify the background used for SEIVpro::GUS lines.
Line 182: I recommend the authors reorder the figure panels in Figure 1 to align with the results' sequence.
Line 192: The authors should include data for Figure 2A (Microscopy of the root xylem tissues) upon treatment with dexamethasone.
Line 231: The authors presented Principal Component Analysis (PCA) of whole transcriptome data, where the two components explain only 27.3% and 17.3% of the variance. The lack of difference between the different seiv mutants is unusual. Does another component of the analysis explain high variance?
Line 242: I suggest the authors include a GO term figure to compare the differences between the different mutants, as

ubiquitination-related SEIV genes play specific roles at certain stages of xylem vessel cell differentiation. Additionally, the authors should explain the rationale behind using a 6-hour post-DEX treatment. The endogenous expression of the SEIV genes is picked at 12 hours for UBP1 and Fbox, 48 hours for PUB46, and 72 hours for PUB36.
Line 258: Can the authors explain why they selected a 6-hour post-DEX treatment?
Line 280: For seiv6 and seiv9 mutants, the ubiquitination levels are significantly reduced in control and DEX treatment, suggesting the contribution of PUB36 and F-box to the overall ubiquitome. Panels C-G suggest that some targets could be present upon VND7 induction. However, the data presentation makes it difficult to draw conclusions. I suggest moving this information to supplementary data panels C to G and adding a multiple Venn diagram with DEX/Mock for VND7-VP16-GR, seiv6, and seiv9.Line 347: The authors demonstrated that VND7 is ubiquitinated at K105 and K260 in both mock- and DEX-treated VND7-VP16-GR but not in the vector control. This raises the question of whether VND7 protein and transcript levels are similar in vector control and VND7-VP16-GR Mock conditions. Additionally, it is important to include the number of diGly peptides found for each ubiquitination site and determine if the difference is significant. Otherwise, consider removing the statement.
Line 347 to 413: it is noted that the discussion section appears overly extensive. The authors are encouraged to condense the description of the SEIV proteins, as it does not directly contribute to the interpretation, explanation, or hypothesis generation based on the data presented in the manuscript. Streamlining this portion of the discussion will help maintain focus on the key findings and their implications.

Minor comments:
Line 261: I suggest substituting "expressed" with "accumulated".
Line 286: The authors should reorder panels 4B to 4I to improve the flow of the results. Additionally, clearer labelling of the comparisons behind each graph would enhance readability and help the reader follow the conclusions.
Lines 298-326: This section of the results only considers DEX/Mock for VND7-VP16-GR and should be moved before line 257 for better coherence.
Line 327: The conclusions of Paragraphs 327-338 overlap with those of lines 278-285. It is advisable to merge both paragraphs for clarity and conciseness
Line 771 and 793: the sentence "four valid values in one of the conditions" is ambiguous, I suggest using "present with at least two peptides in four biological repeats." Clarifying this statement would enhance the understanding of the data analysis methodology.

Reviewer #1 (Comments for the Author):

This manuscript reports several suppressor mutants of VND7-inducible xylem vessel cell differentiation. Thanks to the authors' contribution, VND7 is a relatively well characterized master regulator of xylem vessel differentiation. Proper function of xylem vessel is critical for the plant's growth and survival. Identification and characterization of the mutants lead to multiple testable hypotheses. While the findings described in this report are significant contribution to expanding our knowledge in plant science, the overall impression of this report is that the results are mostly descriptive, rather than the outcome from hypothesis-driven research. I have no doubt that these findings are useful in further study of the role of posttranslational regulation of xylem vessel differentiation and function.

Answer: Thank you for your critical reading of our manuscript. We are pleased to know that you have recognized the value of our research.

Q1. Therefore, additional studies (e.g. genetic complementation) to test the hypotheses that the authors propose will strengthen this manuscript.

Answer to Q1: Thank you for pointing this out. We might have not fully grasped the meaning of 'the hypothesis' you are referring to, but the point of this paper is, as you pointed out, that we showed the importance of protein ubiquitination in xylem vessel cell differentiation, found many candidates of ubiquitination regulatory targets, and furthermore, demonstrated the possibility that the significance of protein ubiquitination lies in the regulation of VND7 activity by VND7 ubiquitination.

If your point is to show the role of ubiquitination of VND7 genetically (e.g. introduce the VND7 genes with mutated ubiquitination sites into the *vnd7* mutant), unfortunately, we have to report that *vnd7* single mutants do not show any clear phenotype due to gene function redundancy in the VND family (Kubo et al., 2005). For this reason, complementation experiments with *vnd7* are currently impossible. We would like to continue to work on this point, including the creation of *vnd* multiple mutants, as it may be possible to confirm our hypothesis critically by using *vnd* multiple mutants. For the SEIV genes, please refer to our answers to Q6 from Reviewer 3.
Thank you again for your valuable comment.

Additional points to improve this manuscript:
Q2. The authors suggested that "the dominant seiv mutations preferentially affect VND7-induced xylem vessel cell differentiation in aboveground tissues." Yet, they used primary roots to examine the impacts of the seiv mutations on endogenous xylem vessel cell differentiation. They need to provide rationale for their choice.

Answer to Q2: We examined the xylem vessel cell differentiation in both aboveground tissues and roots in the *seiv* mutants in the absence of DEX treatment by microscopy observation. However, we did not detect clear visible differences between Col-0, wild-type *VND7-VP16-GR*,

and the *seiv* mutants in the cotyledons, hypocotyls, leave veins, (new Supplemental Fig. S4-5) and primary roots (Fig. 2A). In such cases, we thought it would not make a difference whether to choose roots or aboveground parts for checking the impacts of *seiv* mutations in endogenous xylem vessel element formation.

In addition, while *seiv* mutations predominantly affect VND7-induced xylem vessel cell differentiation in aboveground tissues, RT-qPCR uncovered a reduction in the upregulation of SCW-related genes suggesting that xylem vessel element formation would be affected in root tissues (Pawittra et al., 2020). If we can confirm the negative impact of the *seiv* mutation on endogenous xylem vessel formation in primary roots, it is reasonable to suspect more pronounced effects on endogenous xylem vessel formation in aboveground tissues.

Furthermore, considering the variation growth in aboveground tissues, accessing and observing xylem vessels in primary roots is much more operable and comparable. Hence, we chose primary roots to check the impacts of *seiv* mutations on xylem vessel element formation via fluorescence transport assay.

Q3. The authors did not detect any differences between Col-0, wild-type VND7-VP16-GR, and the seiv mutants in terms of xylem vessel element formation as well as in the number or size of xylem vessels or SCW deposition. Then, they performed xylem performance assay. Again, providing their rationale for the assay will be helpful for readers to understand why they carried out the xylem performance assay when no xylem vessel element formation nor SCW deposition between the control and mutants. Furthermore, the xylem performance assay indicated that xylem vessel function is impaired in the seiv mutants despite no difference in the xylem vessel morphologically. I would like to see the authors' take on this finding in the Discussion.

Answer to Q3: While no significant morphological difference was observed in xylem vessel formation and SCW thickenings between *seiv* mutants and control groups in the absence of DEX treatment, we cannot deny the possibility that the physicochemical properties of xylem cell walls in *seiv* mutant were changed. Particularly, some alternations in cell wall composition and structure are hard to figure out via microscopy observation. In addition, the reduced expression levels of VND7 target genes and GO term analysis in the absence of DEX treatment strongly suggest that *seiv* mutation would impair VND7-dependent endogenous xylem vessel formation (Supplemental Fig. S10 and S11). In the previous study (Endo et al., 2019), several transgenic lines with distinct modifications of xylem cell walls but no visible defects in vascular tissues presented reduced or accumulated transport efficiency, which demonstrated a tight association between the structures of xylem cell walls and xylem transport patterns. Thus, we also performed this xylem transport assay to check whether the cell wall properties in *seiv* mutants are altered or not.

For the conclusive response to Q2&Q3, we put the sentences in line 202-214 as:
"While no significant morphological difference was observed in xylem vessel formation and SCW thickenings between *seiv* mutants and control groups in the absence of DEX treatment, we cannot deny the possibility that the physicochemical properties of xylem cell walls in *seiv*

mutant were changed. Particularly, some alternations in cell wall composition and structure are hard to figure out via microscopy observation. In the previous study (Endo et al., 2019), several transgenic lines with distinct modifications of xylem cell walls but no visible defects in vascular tissues presented reduced or accumulated transport efficiency, which demonstrated a tight association between the structures of xylem cell walls and xylem transport patterns. Thus, we also performed this xylem transport assay to check whether the cell wall properties in *seiv* mutants are altered or not. Considering the growth variations in the aboveground tissues, and observing xylem vessels in primary roots is more operable and comparable, we checked the fluorescence transport ability in primary roots in each line."
Please check them, thank you.

Q4. The RNA-seq data identified many differentially expressed genes, including 61 VND7 target genes, indicating the unique effects of the seiv mutations on transcriptomic changes in response to DEX-induced xylem vessel formation. How would the authors reconcile this with the earlier observations of no differences in xylem element formation between the control and the mutants?

Answer to Q4: The transcriptomic data showed repressed expression levels of VND7 target genes in both the presence and absence of DEX treatment, indicating negative effects of *seiv* mutations on ectopic and endogenous xylem vessel formation, respectively (in current Supplemental Fig. S11). GO enrichment analysis revealed that under the mock treatment, GO terms associated with xylem vessel element formation, i.e., "plant-type secondary cell wall biogenesis", "cell wall polysaccharide metabolic process", and "regulation of secondary cell wall biogenesis", were significantly enriched among downregulated genes in *seiv* mutants compared to wild-type *VND7-VP16-GR* (new Supplemental Fig. S10). These findings strongly suggest that *seiv* mutation would impair VND7-dependent endogenous xylem vessel formation.

Although we did not observed differences in xylem element formation between the control and *seiv* mutants without DEX treatment, we cannot dismiss the impacts of *seiv* mutations on the physicochemical properties of xylem cell walls under normal conditions, which are hard to figure via microscopy observation. Since a previous study (Endo et al., 2019) uncovered an association between the structures of xylem cell walls and xylem transport patterns, we conducted a xylem transport assay and discovered altered transport dynamics in *seiv* mutants. The abnormalities of transport patterns in *seiv* mutants might have implied that the composition or structure of xylem cell walls can be changed due to *seiv* mutations, which is also suggested by the RNA-seq data.

These points were newly mentioned in Discussion in line 475-481 as:
Although we detected no obvious defects in morphology or growth in the *seiv* mutants under normal growth conditions, the xylem transport assay revealed abnormal transport dynamics in the *seiv* mutants (Fig. 2, B and C). Together with transcriptome data analysis indicating that genes for xylem vessel formation were abnormally expressed in *seiv* seedlings (Supplemental Fig. S10 and S11), it can be suggested that alternations in cell wall composition and structure would be caused by *seiv* mutations.

We would like to further make clear these points by future works. Thank you.

Reviewer #3 (Comments for the Author):

The article is well-written, with detailed analyses and a clear presentation of results, although rearranging them to maintain flow would enhance readability. It delineates the pivotal role of the NAC transcription factor VASCULAR-RELATED NAC-DOMAIN7 (VND7) as a master regulator of xylem vessel cell differentiation in Arabidopsis (Arabidopsis thaliana). Additionally, it characterizes seiv mutants (suppressors of ectopic xylem vessel cell differentiation induced by VND7), revealing decreased expression of downstream genes and abnormal xylem transport activity. Upon VND7 induction, ubiquitination levels are modulated, impacting xylem vessel activity. The manuscript further elucidates the role of ubiquitination in regulating VND7 and its effect on xylem vessel function.
The study is exciting, and it is in an area that attracts a lot of interest. The findings presented in this manuscript suggest the potential involvement of one of the SEIV proteins in the ubiquitination of VND7.

Answer: Thank you for your critical reading of our manuscript. Your comment encouraged us to extend our work on post-translational regulation for xylem vessel cell differentiation.

Q5. Despite the significant potential to elucidate the mechanisms underlying VND7 regulation, the authors did not directly test this hypothesis. As a suggestion to strengthen the manuscript, it would be intriguing to investigate whether VND7 is ubiquitinated or deubiquitinated by one of the SEIV proteins. This exploration could provide valuable insights into the regulatory mechanisms governing VND7 activity and contribute to a deeper understanding of xylem vessel cell differentiation.

Answer to Q5: Thank you for this important suggestion. As for the possibility of VND7 being the substrate of SEIV proteins, we conducted a yeast 2-hybrid (Y2H) assay to assess the interaction between VND7 and SEIV proteins. However, our results revealed no direct interaction between them (Supplemental Fig. S16), suggesting that SEIV proteins do not directly target the VND7 protein for its ubiquitination or deubiquitylation.

We put this result at line 426-430 as:
Furthermore, the above findings raise the question of whether VND7 serves as a substrate for SEIV proteins. To investigate this possibility, we conducted a yeast 2-hybrid (Y2H) assay to assess the interaction between VND7 and SEIV proteins. However, our results revealed no direct interaction between them (Supplemental Fig. S16), suggesting that SEIV proteins do not regulate VND7 via ubiquitination or deubiquitylation.

Additional comments:
Q6. Line 120: To validate the point mutation in AT5G18320 (seiv3), AT2G32780 (seiv4), AT5G39250 (seiv6), and AT3G61390 (seiv9), the authors introduced genomic fragments of PUB46,

UBP1, FBX, and PUB36 containing sequences of the coding sequence with the corresponding point mutations into the wild-type VND7-VP16-GR background. Can the authors explain why they did not complement Arabidopsis mutants for the selected genes with the coding sequence with and without the corresponding point mutations? This would validate that only single mutations in these genes are responsible for the suppressed xylem vessel cell differentiation. Have the Arabidopsis mutants for these genes suppressed xylem vessel cell differentiation?

Answer to Q6: Sorry that we did not complement Arabidopsis mutants for the selected genes with the coding sequence with and without the corresponding point mutations, because we have confirmed that these point mutations are dominant, typically resulting in a gain-of-function phenotype. In such cases, we believe that the single mutation alone is sufficient to produce the observed phenotype. In addition, the *pub46* T-DNA insertion mutant lines, such as SALK_015500, SALK_028531, SALK_096071C, and SALK_109233C, have been checked and we did not find any abnormality in seedling growth and xylem vessels. Besides, PUBs, UBP1, and FBX belong to large protein families, so the single mutants are expected not to show growth/developmental defects attributed to gene redundancy within these families.

We guess that we can assess this point using multiple mutants generated hereafter, by crossing single mutants and/or genome editing-based mutagenesis. Thank you for your important suggestion.

Q7. Line 164: The authors should specify the background used for SEIVpro::GUS lines.

Answer to Q7: Thank you for this comment. The background used for *SEIVpro::GUS* lines was the wild-type (Col strain). The detailed information on the *SEIVpro::GUS* reporter driven by the upstream regions of *PUB46, UBP1, FBX, and PUB36* were put in line 166-168 as well as in Materials & methods (line 571-573).

Q8. Line 182: I recommend the authors reorder the figure panels in Figure 1 to align with the results' sequence.

Answer to Q8: Sorry for that, we think these panels are in an appropriate alignment. Fig. 1A was appeared in line 113-114, where is a first place in all panels. Moreover, the order of appearance of panels in Figure 1 is correct. Thus, we didn't change the order of panels. Thank you for your understanding.

Q9. Line 192: The authors should include data for Figure 2A (Microscopy of the root xylem tissues) upon treatment with dexamethasone.

Answer to Q9: We have shown the requested data, i.e. ectopic xylem vessel cell differentiation in primary roots by DEX treatment, in Supplementary Figure 3. Please check it, thank you.

Q10. Line 231: The authors presented Principal Component Analysis (PCA) of whole transcriptome data, where the two components explain only 27.3% and 17.3% of the variance.

The lack of difference between the different seiv mutants is unusual. Does another component of the analysis explain high variance?

Q11. Line 242: I suggest the authors include a GO term figure to compare the differences between the different mutants, as ubiquitination-related SEIV genes play specific roles at certain stages of xylem vessel cell differentiation.

Answer to Q10 & Q11: The scree plot illustrates several principal components derived from the Principal Component Analysis (PCA) of the whole transcriptome data. The first two principal components explain 27.3% and 17.3% of the variance, respectively, with others exhibiting lower eigenvalues and explaining less (panel B in new Supplemental Figure. S12). The transcriptome data comprises six lines and two treatment conditions, reflecting numerous factors contributing to gene expression variability. The modest percentage of variance explained by the first two components suggests that additional influential factors remain unaccounted for. To address this, we generated various PCA scatter plots using different pairs of dimensions and elucidated these dimensions through GO term analysis.

First, GO term analysis revealed significant enrichment of cell wall biosynthesis-related events in the first principal component (panel A in new Supplemental Figure. S12 and new Supplemental Table. S2), indicating a tight association between the first component and VND7-dependent xylem vessel element formation; when the up-regulated genes in response to DEX treatment in the wild-type *VND7-VP16-GR* line were isolated and subjected to PCA, the plot patterns resembled those of the PCA of the whole transcriptome data (please compare Fig. 3A and new Supplemental Fig. S12A). It is clearly consistent with the suppression phenomenon of DEX-induced ectopic xylem vessel cell differentiation in *seiv* mutants, suggesting that the transcriptional changes in the *seiv* mutants under DEX treatment are mainly attributed to disruptions in the DEX-induced program, such as secondary wall formation.

Subsequently, when the second component and other components (Dim3/4/5) were employed as the x- and y-axes, the *seiv* mutants clearly showed different distributions (new Supplemental Fig. S12C-D), which indicated potential differential impacts of *seiv* mutations on various physiological activities in plants. The results of GO enrichment analysis for the second to fifth components are available in new Supplementary Tables S13-16. These impacts of *seiv* mutations may not be triggered by DEX treatment or have been overlooked in such complex data analysis.

In conclusion, it is reasonable that *seiv* mutants exhibited similar plot patterns and clear separation from the wild-type *VND7-VP16-GR* line under DEX treatment with the first and second components as axes. In future studies, we will conduct individual comparative analyses to delve deeper into the effects of *seiv* mutations.

For the response to Q10 and Q11, we put the descriptions in line 252-254, and in line 271-293, and the GO term analysis comparing *seiv* mutants with wild-type *VND7-VP16-GR* line was shown in Supplemental Fig. S8 and S9. Please check the revised parts, thank you.

Q12. RT-qPCR analysis showed that all SEIV genes were upregulated after DEX treatment, with peak expression at different time points, suggesting their specific roles at certain stages of xylem vessel cell differentiation.

Additionally, the authors should explain the rationale behind using a 6-hour post-DEX treatment. The endogenous expression of the SEIV genes is picked at 12 hours for UBP1 and Fbox, 48 hours for PUB46, and 72 hours for PUB36.

Q13. Line 258: Can the authors explain why they selected a 6-hour post-DEX treatment?

Answer to Q12 & Q13: The RNA-seq analysis results have exhibited strong suppression on DEX-induced xylem vessel cell differentiation program at 6-h post-DEX treatment, indicating that the impacts of *seiv* mutations could be detected at the early stages of xylem vessel cell differentiation. Since this is a first report of ubiquitome during xylem vessel cell differentiation, we would like to focus on the first impacts of *seiv* mutations on xylem vessel cell differentiation, and to decrease the complexity of analysis that can be expected when we increase the time points of sampling.

We should also mention that the *seiv* mutations were dominant and expected to be "gain-of-function" type effects on the regulation of protein ubiquitination. As we discussed in the manuscript, we guess the mis-targeting of ubiquitination by the mutated SEIV proteins can be one of reasons for the inhibition of xylem vessel cell differentiation. Thus, the appearance of abnormality on ubiquitome by *seiv* mutations can be possible at the early stages of differentiation, which is earlier than the endogenous SEIV genes are upregulated.

Thank you for your kind understanding.

Q14. Line 280: For seiv6 and seiv9 mutants, the ubiquitination levels are significantly reduced in control and DEX treatment, suggesting the contribution of PUB36 and F-box to the overall ubiquitome. Panels C-G suggest that some targets could be present upon VND7 induction. However, the data presentation makes it difficult to draw conclusions. I suggest moving this information to supplementary data panels C to G and adding a multiple Venn diagram with DEX/Mock for VND7-VP16-GR, seiv6, and seiv9.

Answer to Q14: Thank you for the suggestion. After deep consideration, we would like to keep the current arrangement, since we believe that 1) the dynamics of ubiquitome in the wild type itself is an important basic information to improve our understanding of xylem vessel cell formation, and 2) "how abnormal in ubiquitome in *seiv* mutants" should be presented as the main statement for the context of our discussion.

We agree to the value of multiple Venn diagram, thus we added the data as new Supplementary Fig. S13. Please check it. Thank you for your valuable comment, and kind understanding.

Q15. Line 347: The authors demonstrated that VND7 is ubiquitinated at K105 and K260 in both mock- and DEX-treated VND7-VP16-GR but not in the vector control. This raises the question of whether VND7 protein and transcript levels are similar in vector control and VND7-VP16-GR Mock conditions. Additionally, it is important to include the number of diGly peptides found for each ubiquitination site and determine if the difference is significant. Otherwise, consider

removing the statement.

Answer to Q15: Thank you for the question. We checked our RNA-seq and proteome data, and fund that there is no significant difference in VND7 protein and transcript levels in vector control and *VND7-VP16-GR* under mock treatment. For the details of diGly peptide detection can be referred to new Supplemental Fig. S14. As for the number of diGly peptides in lysine sites, we found it hard to elucidate the significant difference within the table. Therefore, we employed "ND" and "+" to characterize the ubiquitination conditions at each lysine site; however, we found that we did not mention the meaning of "ND" and "+" clearly in the previous manuscript. To make clear the meaning of these labels, we added the caption in the legend of Figure 6, as specifically, "ND" means "not detected", "+" means "detected".

Q16. Line 347 to 413: it is noted that the discussion section appears overly extensive. The authors are encouraged to condense the description of the SEIV proteins, as it does not directly contribute to the interpretation, explanation, or hypothesis generation based on the data presented in the manuscript. Streamlining this portion of the discussion will help maintain focus on the key findings and their implications.

Answer to Q16: Thank you for your suggestion! We have reduced several sentences in Discussion, according to your comment.
(When describing the potential biological roles of the SEIV protein and speculating on the potential effects of mutation sites, we mainly emphasize its potential in environmental responses. This is because xylem vessels play a crucial role not only in plant growth and development but also in plant environmental response. Therefore, please allow us to provide slightly more detailed information about SEIV protein.)

Minor comments:
Q17. Line 261: I suggest substituting "expressed" with "accumulated". xx accumulated, xx reduced respectively.

Answer to Q17: Thank you for this suggestion, it has been revised.

Q18. Line 286: The authors should reorder panels 4B to 4I to improve the flow of the results. Additionally, clearer labelling of the comparisons behind each graph would enhance readability and help the reader follow the conclusions.
Q19. Lines 298-326: This section of the results only considers DEX/Mock for VND7-VP16-GR and should be moved before line 257 for better coherence.
Q20. Line 327: The conclusions of Paragraphs 327-338 overlap with those of lines 278-285. It is advisable to merge both paragraphs for clarity and conciseness

Answer to Q18-20: Thank you for your suggestion. We agree with your comments and rearranged these parts. The results of protein and ubiquitination dynamics in response to VND7 induction (DEX/Mock for *VND7-VP16-GR*) were elucidated in one separate section.

Q21. Line 771 and 793: the sentence "four valid values in one of the conditions" is ambiguous, I suggest using "present with at least two peptides in four biological repeats." Clarifying this statement would enhance the understanding of the data analysis methodology.

Answer to Q21: After this comment, we revised the description as "After grouping samples by condition (control mock, control DEX, VND7 mock, VND7 DEX, *seiv6* mock, *seiv6* DEX, *seiv9* mock, *seiv9* DEX), only those proteins were retained for the subsequent analysis that had four valid values in one of the conditions" in line 831-834. Similarly, we put same description in line 857-858 for better understanding of readers. These revisions now make clear that the data was not grouped by biological replicates but by conditions. Please check them at Line. Thank you.

Dear Dr. Misato Ohtani:

We have received reviews of your manuscript entitled "Active protein ubiquitination regulates xylem vessel functionality." On the basis of the advice received, the board of reviewing editors would like to accept your manuscript for publication in The Plant Cell. This acceptance is contingent on revision based on the comments of our reviewers. In particular, please consider the following:

Respond to the minor comment of the reviewer, and describe the caveats of the lack of additional mutants to support your conclusions, as per this reviewer.

Please highlight all changes and include a detailed annotation of changes of the text, with line numbers, and noting your responses to the comments.

To submit your revised manuscript, click:

Link Not Available

If you have any questions about the revision submission procedures, please contact the Editorial Office Staff (tpc-submissions@aspb.org). If you cannot return the revised manuscript within 30 days, please let us know. Otherwise, we will assume that you have elected not to revise the manuscript and withdraw it.

Thank you very much for the privilege of reviewing this work. I look forward to receiving the next version.

Sincerely,

The Plant Cell Board of Editors

Reviewer #3 (Comments for the Author):

Thank you for addressing my comments and suggestions.

While most of my comments have been fully addressed, I still believe that additional experiments -specifically, genetic complementation of knock-out lines for selected genes with the coding sequence both with and without the corresponding point mutations-would further strengthen this manuscript.

Regarding lines 33-34, please clarify which protein groups you are referring to, as this is the first time they have been mentioned in the text.

Please also note the following:

-The Plant Cell now requires authors to complete and submit an author revisions checklist upon submission of a revised manuscript. The aim of the checklist is to aid authors in preparing a high-quality manuscript, facilitate the review and assessment of revised manuscripts, and help to ensure that journal standards are maintained across the board. If your manuscript is accepted, the completed checklist will be published as supplemental material attached to the article online. Please download a copy of the checklist (pdf fillable form) at this link, for submission with your revised manuscript: https://tpc.msubmit.net/html/Author_Revisions_Checklist.pdf.

-Supplemental materials should be restricted to large datasets and tables, presentation of replicates, and validation of reagents, methods, or genotypes. Any data that are used to support the major claims must be in the main manuscript. Supplemental figure legends must indicate what figure in the main manuscript is supported by the supplemental data presented. Please justify how each of the supplemental figures meet the criteria.

-Sampling methods and the nature of "biological replicates" should be described precisely (i.e. different plants, parts of plants, pooled tissue, independent pools of tissue, sampled at different times, etc.), along with a clear description of and rationale for any statistical analyses conducted. The reader should know exactly what was sampled; what forms the basis of the calculation

of any means and statistical parameters reported. This is also necessary to ensure that proper statistical analysis was conducted.

Response to Reviewer

1. (Reviewer #3) I still believe that additional experiments-specifically, genetic complementation of knock-out lines for selected genes with the coding sequence both with and without the corresponding point mutations-would further strengthen this manuscript.

Answer: Thank you for your further suggestions. As you suggested, the additional complementation test will possibly strengthen our message. However, unfortunately, we could not easily perform effective complementation tests with knock-out lines, given the fact that the *seiv* mutant alleles identified here are dominant. It is expected that the genetic complementation of the mutated *SEIV* genes into knock-out lines would show similar results as the insertion of the mutated *SEIV* genes into the wild-type. In addition, at the moment, the T-DNA insertion mutations in *SEIV* genes do not exhibit significant morphological abnormalities in xylem vessels, leading to the possibility of gene redundancy effects. Thus the complementation test with the wild-type *SEIV* genes into T-DNA mutants would be also not fruitful to obtain additional information on the ubiquitination regulation for xylem vessel formation.
However, we highly appreciate your suggestion on the complementation test, since you have us realize the importance of genetics approach for further analysis. Our next steps of SEIV protein functional analysis should contain such genetics materials with multiple mutations.

After your suggestion and Editor's instruction, we stated that additional genetic materials are not currently available for *SEIV* genes and it would be a future challenge.
Please see line 500-505, we said now "In addition, we could not obtain additional genetic materials on any SEIV gene loci. As the *seiv* mutants obtained in our screening are dominant, thus the genetic complementation experiments are impossible to be performed for the seiv phenotypes defective for xylem transport activity (Fig. 2, B and C). These points should be tested with new techniques and/or new mutant materials, for further analysis of protein ubiquitination in xylem vessel cell differentiation."

Thank you again for your comments.

2.>review#3 Regarding lines 33-34, please clarify which protein groups you are referring to, as this is the first time they have been mentioned in the text.

Answer: Thank you for your suggestion.
The sentence you pointed out is describing the number of proteins with increased and decreased ubiquitination levels as an overall picture of ubiquitome analysis. The following text introduces the function of protein groups upregulated for the ubiquitination by the VND7 induction, as a significant case of ubiquitination dynamics. Line 33-34 is within Abstract, thus we believe that the current description is enough to summarize our results and decided to leave it after deep consideration. Thank you for your kind understanding.

3.>editor Please also reiterate in the discussion that there are likely changes in

physicochemical aspects of xylem secondary cell wall composition that cannot be identified given the paucity of tools available to do so at cellular resolution.

Answer: According to your comment, we added such mention in line 498-500. Thank you for your valuable suggestion.

Dear Dr. Misato Ohtani:

We are pleased to inform you that your paper entitled "Active protein ubiquitination regulates xylem vessel functionality" has been accepted for publication in The Plant Cell, pending a final editorial review by a science editor. At this stage, your manuscript will be evaluated by a science editor with respect to its presentation of scientific content, compliance with journal policies, and presentation for a broad readership. The Plant Cell has contracted with Plant Editors (planteditors.com) to provide this service to our authors, and you will soon receive additional information on this process.

Please note that each author needs to link their ORCID identifier to their account in the system before your manuscript can be published. If any authors do not have an ORCID linked to their account, they will receive a message with a link to complete this task. Please ensure that ALL of your coauthors have completed this task as soon as possible.

ASPB offers an OPEN option that allows authors to have their online articles available for free to all users immediately upon publication. For more information about the ASPB OPEN option, refer to the Final Submission Checklist Form.

The Plant Cell and The Arabidopsis Information Resource (TAIR) are collaborating to collect functional annotation data about Arabidopsis genes from authors. This includes information about the gene's molecular function (e.g., kinase activity, ATP synthetase activity), the biological process/es it is involved in (e.g., endosperm development, threonine biosynthesis), its subcellular location (e.g., nucleus, ER), anatomical or developmental expression pattern (e.g., leaf, ovule, flower stage 10, seedling stage), or its partner in a protein-protein interaction (e.g., AT1G01010 interacts with AT1G01020).

If your paper contains results falling into one or more of these categories for Arabidopsis genes, we request that you now submit these data for inclusion in TAIR by filling in the form provided at the following URL: https://www.arabidopsis.org/doc/submit/functional_annotation/123. If you need further clarification on what types of data can be submitted please contact curator@arabidopsis.org.

Finally, we encourage your submission of artwork for the journal cover. Monthly issues will have an online cover image and selected covers will be used for posters, other promotional items, and "wallpaper" for mobile devices. For more information, refer to Cover Submission in the Instructions for Authors https://tpc.msubmit.net/cgi-bin/main.plex?form_type=display_auth_instructions.

We look forward to seeing your paper published.

Sincerely,

The Plant Cell Board of Editors

=====
IMPORTANT REMINDER: PEER REVIEW REPORTS
=====

If you opted to publish a peer review report along with your article during the original submission process, it will be prepared by the editorial staff and publicly posted with your manuscript, inside the zip file that contains any other supplemental material. As a reminder, the peer review report is a public record of all comments from editors and reviewers, as well as your prior responses, as you received them in the decision letters for each draft of your manuscript. If you agreed to publish this report and have changed your mind, or are not sure if you selected this option, please contact the editorial office as soon as possible before signing the license agreement from our publisher.

=====

----FOR ASPB OFFICE USE ONLY (DO NOT EDIT)----
MSID: 38065
Corresponding Author: Dr. Ohtani
